# Data on generation of Kekulé structures for graphenes, graphynes, nanotubes and fullerenes and their aza-analogs

**DOI:** 10.1016/j.dib.2018.10.128

**Published:** 2018-11-01

**Authors:** Sergey Trepalin, Sasha Gurke, Mikhail Akhukov, Andrey Knizhnik, Boris Potapkin

**Affiliations:** a Institute of Physiologically Active Compounds, Russian Academy of Sciences, Chernogolovka, Moscow Region 142432, Russia; b Elsevier Inc., 230 Park Ave., 8th Floor, New York, NY 10169, USA; c Kintech Lab, 12 3rd Khoroshevskaya Str., Moscow 123298, Russia

## Abstract

Two new features are added to existing algorithms for kekulization of chemical structures, i.e., handling of triple and cumulene bonds in cycles and use of random atom sorting to remove unmatched atoms. Handling of triple and cumulene bonds enables kekulization of graphynes and graphdiynes. Random sorting speeds up the calculation time, i.e., kekulization of large chemical structures containing about 10^7^ atoms takes ≤1 min on a typical PC. Source codes (Pascal, GNU GPL license) are included as a compiled application (Windows 64). Calculation times and unmatched atom statistics are provided for graphenes, graphynes, nanotubes, graphyne nanotubes and fullerenes. Benchmark comparisons are made for some data.

**Specifications table**

**Table:** 

**Subject area**	*Chemistry (General)*
**More specific subject area**	*Kekulization of large chemical structures*
**Type of data**	*Tables, text file, figures, computer-readable chemical structures*
**How data was acquired**	*Calculation using Notebook MSI GE 60 2PG Apache*
**Data format**	*.mol, *.xyz and *.cc1 files for chemical structures
**Experimental factors**	*2.8MHz i7 processor, 16G RAM, Windows 2012 server*
**Experimental features**	*Single-thread application; Win API method GetTickCount() used for precise time measurements*
**Data source location**	*Article text for source code and calculation statistics*, Attachment1.zip*for compiled application*, Attachment2.zip*for chemical structures*
**Data accessibility**	*Downloadable .zip*
**Related research article**	*Trepalin S., Gurke S., Akhukov M., Knizhnik A., Potapkin B., A fast approximate algorithm for determining bond orders in large polycyclic structures, Journal of Molecular Graphics and Modelling, vol. 86 (2019), pp. 52-65*

**Value of the data**

• *This data proves the viability of a fast algorithm for the modeling of state-of-the art materials such as graphenes in scientific and commercial applications*
• *The data is provided for very large chemical structures (10*^*7*^*-10*^*8*^ *atoms) and is obtained using ordinary hardware with short calculation times*
• *This data has been benchmarked against existing algorithms and can be used for benchmarking for the future improvements to the algorithm*

## 1 Data

Detailed description of an algorithm for fast generation of Kekulé structures from a list of atomic valences and connectivity matrices is given in [1]. The source code that was based on this algorithm and used for calculations is provided in this publication.

Model calculations were performed for graphenes, nanotubes, fullerenes and their aza-analogs, polycyclopentadienes and porphine, using a Windows 2012 server with 2.8 GHz i7 processor and 16 GB RAM. The procedures were not run in parallel but rather in a single thread. The Win API method GetTickCount() was used for precise time measurements. This method allows the measurement of the elapsed time with a millisecond precision. For the compounds containing less than 1,000,000 atoms, the computing time was determined as an average of 1000 calculations, less than 10,000,000 atoms – as an average of 100 calculations and for more than 10,000,000 atoms – as the time of one calculation.

For repeated calculations, the sorting order of the sequence of chemical bonds was random. For an algorithm of aromatic bond alternation, this is equivalent to the random selection of a double bond in the node. For a large number of calculations, this procedure allows estimation of the statistical distribution of unmatched atoms after alternation of aromatic bonds.

Compiled application (Windows 64) used for calculations is in the Attachment1.zip. Computer-readable chemical structures are in the Attachment2.zip.

## 2 Experimental design, materials and methods

### 2.1 Nanotubes

**Fig. 1:**
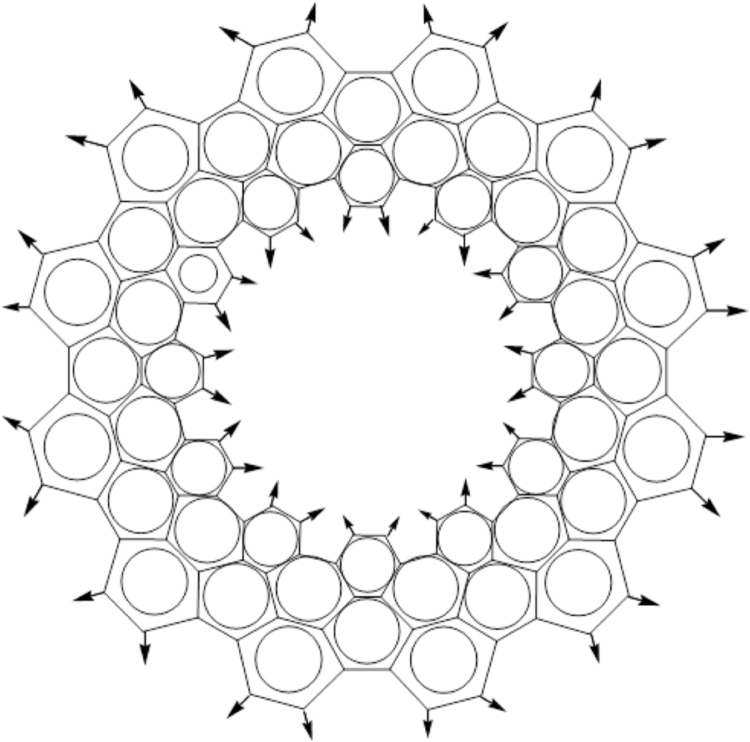
The pattern for generation of C[12,12] nanotubes.

Carbon nanotubes of various lengths were generated using the pattern shown in Fig. 1. Generated nanotubes contain two acyclic methylene groups at the beginning and at the end of a nanotube.

In Fig. 1, the arrows show the points of attachment for generation of a polymer molecule. The points of attachment for the outer rings connect with the points of attachment for the inner rings. The order of an aromatic bond resulting from connecting a pair of attachment points is unknown. For the end moieties of the polymer, the points of attachment were replaced with single bonds to hydrogen atoms. The results of calculation are given in Table 1 and discussed below.

**Table 1:** Time required for generation of Kekulé structures and unmatched atom statistics for nanotubes and graphenes.

Chain length	Formula	Time (ms)	Unmatched atoms statistics																				
			2	4	6	8	10	12	14	16	18	20	22	24	26	28	30	32	34	36	38	40	42
Nanotubes																							
1	C_144_ H_48_	0.33		32	351	510	107																
10	C_1440_ H_48_	2.42						1	28	93	111	146	232	215	113	9	3						
100	C_14400_ H_48_	28.1									5	12	15	36	119	270							
1000	C_144000_ H_48_	407.7										1	1	1	12	91	281	332	227	51	3		
10000	C_1440000_ H_48_	8123.3															6	30	34	23	7		
100000	C_14400000_ H_48_	108437																1					
Graphenes																							
1	C_144_H_38_	0.16	31	340	442	182	5																
10	C_1440_ H_146_	2.09						9	42	97	140	166	199	167	107	58	15						
100	C_14400_ H_1226_	28.5									6	14	12	20	24	77	165	255	248	138	37	4	
1000	C_144000_ H_12026_	451.8										2	2	2	1	5	7	36	168	421	288	65	3
10000	C_1440000_ H_120026_	8497.8																1	11	37	42	9	
100000	C_14400000_ H_1200026_	118297																		1			

Data for some compounds from [2] obtained with our algorithm																							
tube-980	C_980_H_26_	1.36	17	75	164	312	246	143	39	4													
sheet-1800	C_1800_H_178_	2.19	0	0	0	0	0	0	0	0													
sheet-19602	C_19602_H_592_	28.9	0	0	0	0	0	0	0	0													

Where:

Chain length – number of monomeric units in a polymer,

Formula – molecular formula,

Time (ms) – time required for kekulization of a single chemical structure in milliseconds,

Unmatched atoms statistic - number of calculations for a given number of unmatched atoms (2-42).

### 2.2 Graphene

**Fig. 2:**
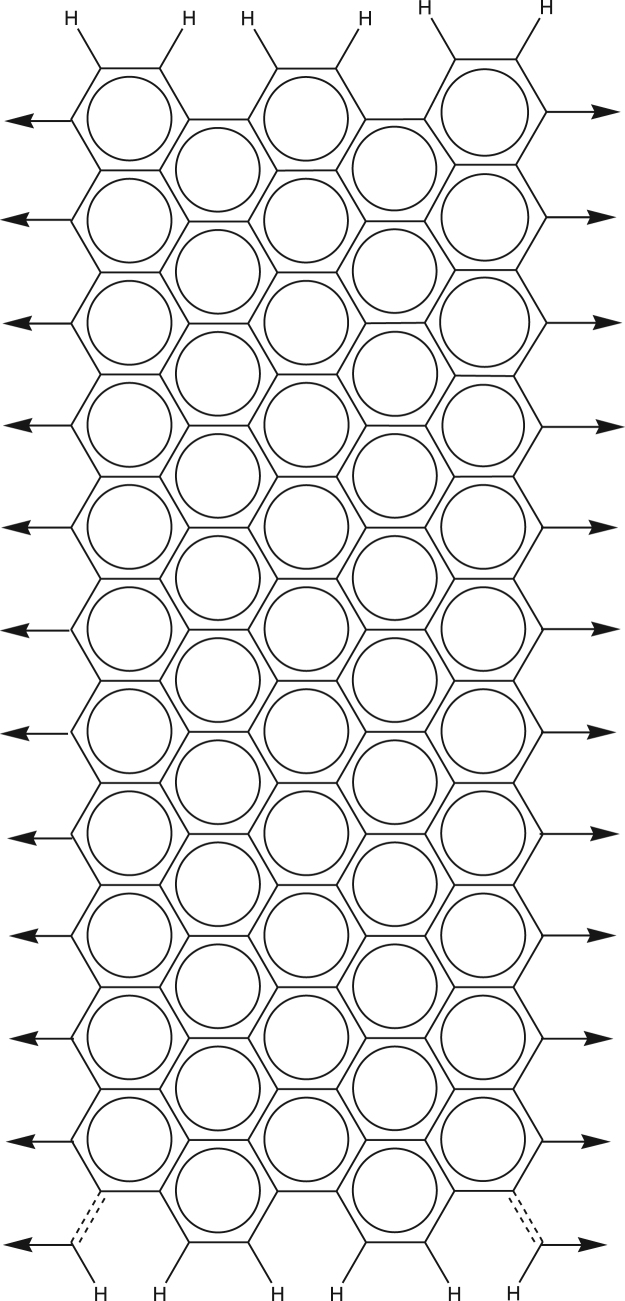
The pattern for generation of graphenes.

The pattern for generation of graphene is shown in Fig. 2.

To generate polymers, the points of attachment on the left were connected with the points of attachment on the right of another graphene block in the way similar to that used for nanotubes. The points of attachment of the first and the last blocks were capped with hydrogen atoms via single bonds. For acyclic carbon atoms, required for the generation of repeating aromatic cycles, two hydrogen atoms were added to the point of attachment, resulting in a methylene group. The results of calculations are given in Table 1.

Kekulization of various compounds, including nanotubes and graphenes, is described [2], [3] and computing times are provided. For comparison, we performed calculations for some of the same compounds. The results are in Table 1. Computing times were dramatically shorter than those reported in [2].

Detailed discussion of this data can be found in [1].

### 2.3 Fullerenes and porphine

**Fig. 3:**
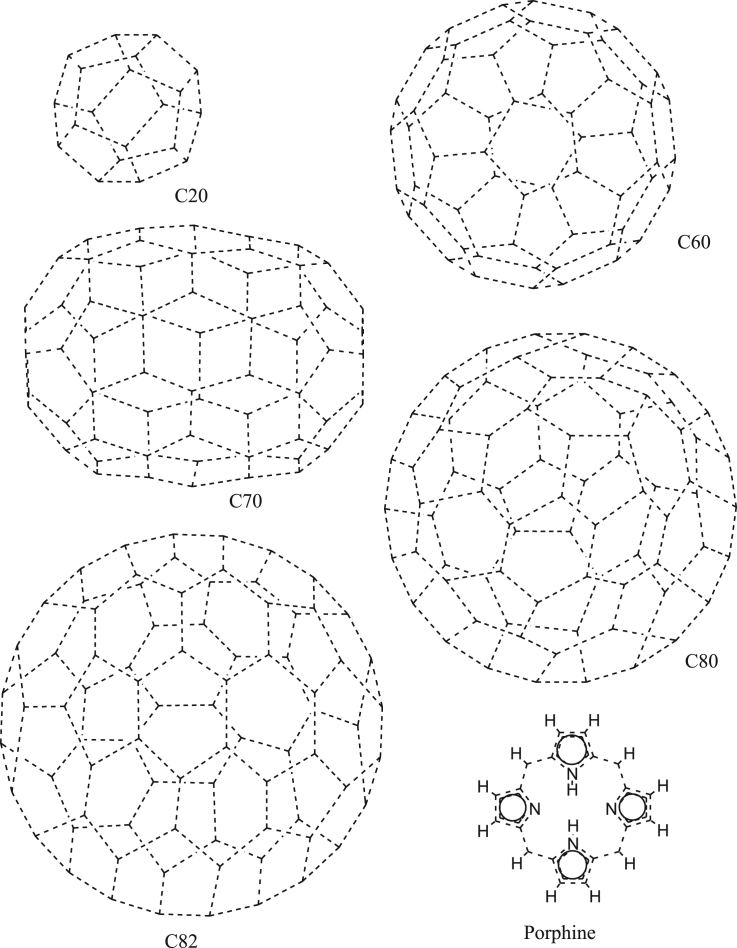
Fullerenes and porphine used for model calculations.

Alternation of aromatic bonds was studied for fullerenes C20, C60, C70, C80, C82, their random aza-analogs, as well as for porphine (Fig. 3).

Polymeric analogs of these compounds do not exist. Consequently, all the calculations were performed for monomers. To validate the efficiency of the algorithm for five-member cycles, model calculations were performed for azafullerenes by randomly replacing 2, 4 or 8 carbon atoms with nitrogen. Nitrogen has a valence of 3 and three single converging bonds in each node. This substitution can be done for the even number of atoms only. Otherwise, bonds cannot be alternated, and the number of unmatched atoms is odd. The results of calculations are given in Table 2.

**Table 2:** Time required for generation of Kekulé structures and unmatched atoms statistics before their removal for fullerenes, their aza-analogs and porphine. The data is for 1000 transactions.

Compound	Formula	Time (ms)	No. non-existent	Avg. no. iterations	Max. no. iterations	Unmatched atoms statistic					
						0	2	4	6	8	>8
С20	С_20_	0.078	0	1.00	1	699	301				
Diaza-C20	C_18_N_2_	0.062	0	1.03	3	409	583	8			
Tetraaza-C20	C_16_N_4_	0.438	33 + 59	14.2	2	499	493	8			
Octaaza-C20	C_12_N_8_	0.953	541 + 218	70.6	1	769	222	9			
C60	C_60_	0.156	0	1.00	1	329	600	68	3		
Diaza-C60	C_58_N_2_	0.171	0	1.01	2	124	681	182	11	1	1
Tetraaza-C60	C_56_N_4_	0.234	4 + 6	3.72	3	78	556	337	28	1	
Octaaza-C60	C_52_N_8_	1.250	63 + 83	24.4	3	146	492	306	54	1	1
C70	C_70_	0.187	0	1.003	2	264	656	76	4		
Diaza-C70	C_68_N_2_	0.188	0	1.01	3	102	269	24			
Tetraaza-C70	C_66_N_4_	0.407	2 + 3	2.54	3	61	498	382	57	2	
Octaaza-C70	C_62_N_8_	0.890	49 + 52	14.86	3	79	419	390	103	9	
C80	C_80_	0.187	0	1.093	3	291	615	94			
Diaza-C80	C_78_N_2_	0.203	0	1.065	3	90	577	312	21		
Tetraaza-C80	C_76_N_4_	0.344	1 + 5	1.69	4	50	421	440	84	5	
Octaaza-C80	C_72_N_8_	0.860	31 + 42	12.8	4	55	303	461	162	18	1
C82	C_82_	0.203	0	1.014	2	223	622	149	5	1	
Diaza-C82	C_80_N_2_	0.203	0	1.020	2	74	547	347	30	2	
Tetraaza-C82	C_78_N_4_	0.328	2 + 4	2.84	2	42	450	425	78	5	
Octaaza-C82	C_74_N_8_	0.984	28 + 37	13.97	3	46	288	468	175	21	2
Porphine	C_20_H_14_N_4_	0.093	0	1.00	1	441	559				

Where:

Compound – chemical structure in Fig. 2,

Formula – molecular formula,

Time (ms) – time required for kekulization of a single chemical structure in milliseconds,

No. non-existent – number of compounds from a 1000 set for which Kekulé structure was not generated,

Avg.no. iterations – average number of shuffles. This number includes the maximum number of shuffles (300) after which a decision is made that a Kekulé structure does not exist,

Max. no. iterations – maximum number of shuffles required for successful kekulization,

Unmatched atom statistic - number of calculations for a given number of unmatched atoms (0->8).

Detailed discussion of this data can be found in [1].

In addition, model calculations were performed for 2488 fullerenes from a library by Yoshida [4]. This data is provided in Table S3.

The legend for the column headers in this table is the same as for Table 2, except the column No. non-existent is not provided because every compound from the set of 1000 had a Kekulé structure.

### 2.4 Graphynes and graphyne nanotubes

**Fig. 4:**
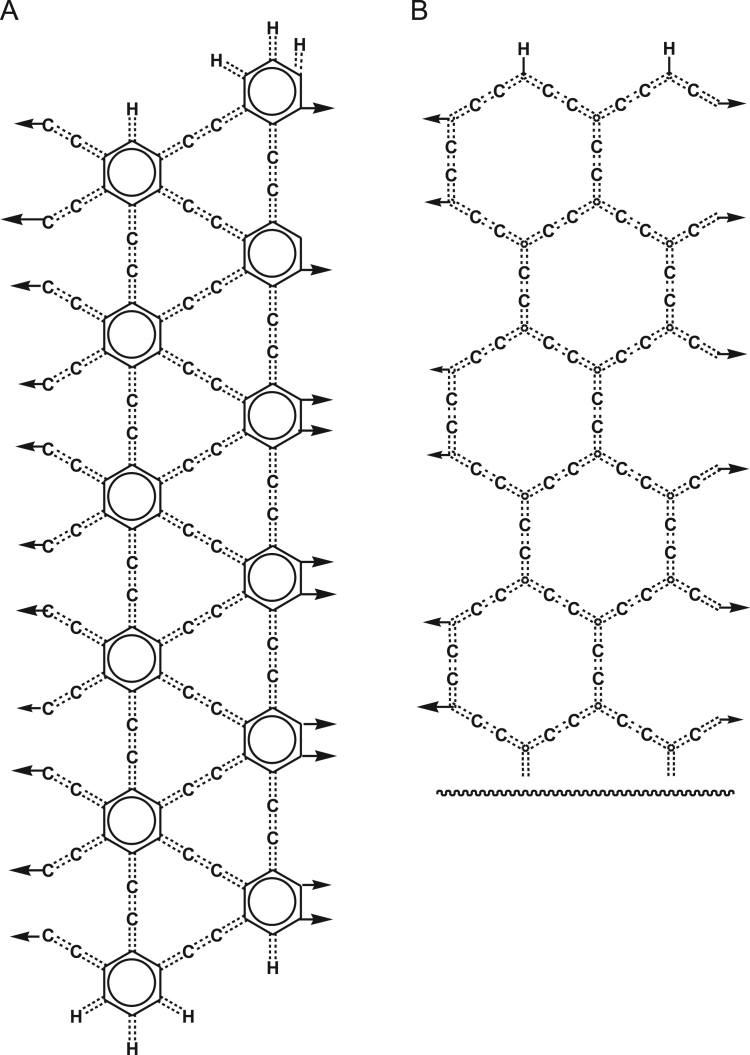
Monomer units of graphynes GY1 (A) and G7 (B). Upper half of GY7 monomer unit is shown only. The entire monomer could be visualized by connecting the upper part with its reflection relative to the zig-zag line, using two aromatic bonds.

We studied graphynes GY1 and GY7 (Fig. 4) and graphyne nanotubes of various degrees of polymerization. Graphyne nanotubes were generated by replacing hydrogen atoms in GY7 with carbon atoms and adding a bond between these atoms in a vertical position.

The times required for kekulization of graphynes and graphyne nanotubes of various degrees of polymerization are shown in Table 3.

**Table 3:** Kekulization of graphynes and graphyne nanotubes of various degrees of polymerization.

Chain length	1		10		100		1000		10,000		100,000	
	Formula	Time (ms)	Formula	Time (ms)	Formula	Time (ms)	Formula	Time (ms)	Formula	Time (ms)	Formula	Time (ms)
Tube	C_192_ H_72_	0.5	C_1920_ H_72_	2.06	C_19200_ H_72_	29.8	C_192000_H_72_	332	C_1920000_ H_72_	7201	C_19200000_ H_72_	59,515
GY1	C_136_ H_74_	0.45	C_1360_ H_146_	1.36	C_13600_ H_866_	13.7	C_136000_ H_8066_	176	C_1360000_ H_80066_	4811	C_13600000_ H_800066_	75,938
GY7	C_188_ H_86_	0.39	C_1880_ H_122_	2.44	C_18800_ H_482_	25.3	C_188000_ H_4082_	358	C_1880000_ H_40082_	6414	C_18800000_ H_400082_	82,032

### 2.5 Polycyclopentadienes

**Fig. 5:**
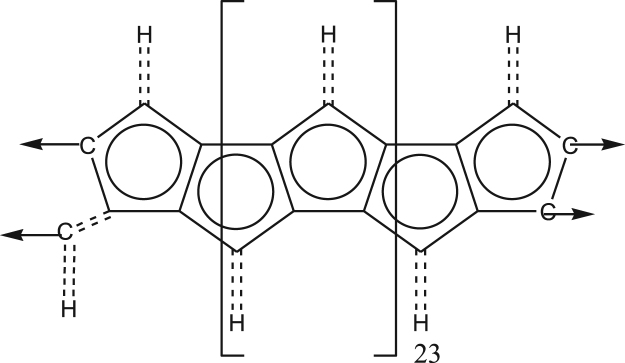
Structural repeating unit of polycyclopentadiene.

Polycyclopentadienes (Fig. 5) are remarkable because they contain odd-sized cycles and can be easily generated as long-chain polymers.

We studied three types of polycyclopentadienes. In the first type, after generation of the structure, free valences of the end-group carbon atoms with arrows were replaced with hydrogens. That resulted in a methylene end group. In the second type, free valences in the left-hand end group were combined with these in the right-hand end group to form a cycle. In the third type, end groups were combined in a crisscross fashion for form a Moebius loop. The results of calculations are shown in Table 4.

**Table 4:** Results of generation of Kekulé structures for polycyclopentadienes.

Chain length	Formula	Time (ms)	No. non-existent	Avg. no. iterations	Max. no. iterations	Unmatched atoms statistic			
						0	2	4	6
Linear									
1	C_150_ H_54_	0.53	0 + 0	1.93	13	372	607	21	0
10	C_1500_H_504_	4.08	0 + 0	4.63	25	747	250	3	0
10	C_1498_H_504_N_2_	35.2	0 + 10(1)	22.9	264	477	431	81	1
10	C_1496_H_504_N_4_	65.75	4 + 91(71)	68.9	300	262	393	210	31
10	C_1492_H_504_N_8_	189	87 + 348(261)	163	299	50	183	269	247
100	C_15000_H_5004_	67.7	0 + 0	5.60	36	852	146	2	0
1000	C_150000_H_50004_	1192	0 + 0	5.61	47	850	150	0	0
10000	C_1500000_ H_500004_	47354	0 + 0	7.57	33	89	11	0	0
100000	C_15000000_ H_5000004_	505401	0 + 0	6.5	22	7	3	0	0
Cyclic									
1	C_150_H_50_	0.34	0 + 0	1,23	5	289	681	30	0
10	C_1500_H_500_	2.77	0 + 0	2.89	17	624	332	44	0
100	C_15000_H_5000_	33.6	0 + 0	2.90	28	622	331	47	0
1000	C_15000_H_50000_	585	0 + 0	2.80	14	634	335	31	0
10000	C_1500000_ H_500000_	16368	0 + 0	3.09	12	69	29	2	0
100000	C_15000000_ H_5000000_	193859	0 + 0	2.2	6	7	2	1	0
Möbius									
1	C_150_H_50_	0.36	0 + 0	1.26	4	189	687	121	3
10	C_1500_H_500_	3.67	0 + 0	3.66	29	544	382	73	1
100	C_15000_H_5000_	52.2	0 + 0	4.41	27	616	332	51	1
1000	C_150000_H_50000_	1381	0 + 0	4.42	29	602	350	48	0
10000	C_1500000_ H_500000_	24147	0 + 0	3.75	26	52	42	6	0
100000	C_15000000_ H_5000000_	604359	0 + 0	6.9	28	8	0	2	0

The legend for the column headers is the same as for Table 2. Parenthetical values in the column No. non-existent are the counts of structures for which Kekulé representations were found using a backtrack algorithm [5].

The calculation statistics for polycyclopentadienes differ from those for the rest of studied compounds. Specifically, 100 calculations were performed for the number of atoms in the 10^6^-10^7^ range and 10 calculations – for the number of atoms >10^7^. The increase in the number of calculations was due to the probabilistic nature of the algorithm for polycyclopentadienes, requiring multiple initial approximations for the generation of Kekulé structures.

## Footnotes

Appendix A Supplementary data associated with this article can be found in the online version at https://doi.org/10.1016/j.dib.2018.10.128.
