# Supplementary material for: Data on generation of Kekulé structures for graphenes, graphynes, nanotubes and fullerenes and their aza-analogs
Source: Data Brief. 2018 Nov 1;21:1166–77. doi: 10.1016/j.dib.2018.10.128 (PMC6258249; doi:10.1016/j.dib.2018.10.128)
Supplement: Supplementary file 10 — Supplementary material [file mmc10.docx]

- 1. **Source code**

**unit** Kekulize;

**interface**

*{*

*Module Kekulize - restore chemical bond orders*

*Copyright (c), Kinetic Technologies Ltd. 2003-2018 All Rights Reserved.*

*Author : Sergey Trepalin, trep@kintechlab.com*

*Project : MolStructure*

*This library is free software; you can redistribute it and/or*

*modify it under the terms of the GNU Lesser General Public*

*License as published by the Free Software Foundation; either*

*version 2.1 of the License, or (at your option) any later version.*

*This library is distributed in the hope that it will be useful,*

*but WITHOUT ANY WARRANTY; without even the implied warranty of*

*MERCHANTABILITY or FITNESS FOR A PARTICULAR PURPOSE. See the GNU*

*Lesser General Public License for more details.*

*You should have received a copy of the GNU Lesser General Public*

*License along with this library; if not, write to the Free Software*

*Foundation, Inc., 51 Franklin Street, Fifth Floor, Boston, MA 02110-1301 USA*

*}*

**var**

TRY_MAX:integer=300;

**function** restoreBondOrders(**const** valencyList:**array** **of** integer; **const** an1List,an2List:**array** **of** integer;

**var** orderList:**array** **of** integer; **var** nHoles,nPass:integer):integer;

*{*

*Input parameters:*

*valencyList - valency of atom - field V in atom lists (A, Asmall, newAsmall, etc)*

*Array does not changed*

*an1List - first atom forming bond - field AN1 in bond list (B, Bsmall, newBsmall, etc)*

*an2List - secind atom forming bond - field AN2 in bond list (B, Bsmall, newBsmall, etc)*

*Arrays do not changed*

*orderList - bond order: 0-unknown, 1-single, 2-doble, 3-triple. Field BV in bond list (B, Bsmall, newBsmall, etc)*

*On input the array has usually all 0 values*

*nHoles, nPass - have no meaning on input*

*Output parameters:*

*orderList - restored bond order values. If kekulization was successfull, this array does not contains zeroes*

*nHoles - number of 'holes' - for research purposes only-see article*

*nPass - how many times kekulization took place before success (or max number exausted)*

*Return value*

*Zero if kekulization was performed successfully, positive - number of 'holes' (bad kekulization)*

*}*

**implementation**

**uses** variants, SysUtils, DynaList;

*//DynaList is non-standard Delphi library. It is included in sources*

**resourcestring**

BAD_VALENCY='Bad valency %d or number of bonds for atom %d';

BAD_NUMBER_OF_BONDS='Number of bonds %d, attached to atom %d is greater, than specified valency: %d';

BAD_VALENCY_NUMBER_BOND='Atom number %d must have valency 4 and 2 attached bonds. Really it has valency %d and %d attached bonds.';

LOGIC_ERROR='Logic error';

**const**

CONN_MAX=8;

MAX_CHAIN=10000;

**type**

EAlgorithmError=**class**(Exception)

**end**;

TConnBonds=**packed** **record**

nb:integer;

bn:**array**[0..CONN_MAX-1] **of** integer;

**end**;

**function** findYnChain(atomNo:integer; **const** valencyList,atom1List,atom2List:**array** **of** integer; **const** connection:**array** **of** TConnBonds; **var** chain,bonds:**array** **of** integer; **var** atom1,atom2:integer):integer;

**var**

currentAtom,currentBond,prevBond,prevAtom:integer;

bondCount:integer;

test:boolean;

**begin**

test:=((connection[atomNo].nb=2) **and** (valencyList[atomNo]=4));

**if** **not** test **then** **begin**

**raise** EAlgorithmError.create(format(BAD_VALENCY_NUMBER_BOND,[atomNo,valencyList[atomNo],connection[atomNo].nb]));

**end**;

atom1:=-1;

atom2:=-1;

bondCount:=0;

chain[0]:=atomNo;

result:=1;

*//first direction*

prevAtom:=atomNo;

prevBond:=connection[prevAtom].bn[0];

test:=true;

**while** (test) **do** **begin**

**if** (connection[prevAtom].bn[0]=prevBond) **then** currentBond:=connection[prevAtom].bn[1] **else** currentBond:=connection[prevAtom].bn[0];

**if** (atom1List[currentBond]=prevAtom) **then** currentAtom:=atom2List[currentBond] **else** currentAtom:=atom1List[currentBond];

test:=((connection[currentAtom].nb=2) **and** (valencyList[currentAtom]=4));

**if** (test) **then** **begin**

chain[result]:=currentAtom;

inc(result);

prevBond:=currentBond;

prevAtom:=currentAtom;

**end** **else** **begin**

atom1:=currentAtom;

bondCount:=1;

bonds[0]:=currentBond;

**end**;

**end**;

*//second direction*

prevAtom:=atomNo;

prevBond:=connection[prevAtom].bn[1];

test:=true;

**while** (test) **do** **begin**

**if** (connection[prevAtom].bn[0]=prevBond) **then** currentBond:=connection[prevAtom].bn[1] **else** currentBond:=connection[prevAtom].bn[0];

**if** (atom1List[currentBond]=prevAtom) **then** currentAtom:=atom2List[currentBond] **else** currentAtom:=atom1List[currentBond];

test:=((connection[currentAtom].nb=2) **and** (valencyList[currentAtom]=4));

**if** (test) **then** **begin**

chain[result]:=currentAtom;

inc(result);

prevBond:=currentBond;

prevAtom:=currentAtom;

**end** **else** **begin**

atom2:=currentAtom;

**end**;

**end**;

*//formation of bonds chain-required to alternate yn or polyenes chain*

*//they must be store in those order, as chain create to alternate*

prevAtom:=atom1;

prevBond:=bonds[0];

test:=true;

**while** (test) **do** **begin**

**if** (atom1List[prevBond]=prevAtom) **then** currentAtom:=atom2List[prevBond] **else** currentAtom:=atom1List[prevBond];

**if** currentAtom=atom2 **then** test:=false **else** **begin**

**if** (connection[currentAtom].bn[0]=prevBond) **then** currentBond:=connection[currentAtom].bn[1] **else** currentBond:=connection[currentAtom].bn[0];

bonds[bondCount]:=currentBond;

inc(bondCount);

prevBond:=currentBond;

prevAtom:=currentAtom;

**end**;

**end**;

**end**;

**function** findQuick(**const** data:**array** **of** integer; dataSize,probe:integer):integer;

**var**

iLo,iHi,iAvg,n:integer;

**begin**

result:=-1;

**if** (dataSize=0) **then** exit;

iLo:=0;

iHi:=dataSize-1;

**repeat**

iAvg:=(iLo+iHi) **div** 2;

n:=data[iAvg];

**if** n<probe **then** iLo:=iAvg+1 **else**

**if** n>probe **then** iHi:=iAvg-1 **else** result:=iAvg;

**until** (result>=0) **or** (iLo>iHi);

**end**;

**procedure** quickSort(**var** arrayData:**array** **of** integer; iLo,iHi:Integer);

**var**

lo,hi:Integer;

mid,n:integer;

**begin**

lo:=iLo;

hi:=iHi;

mid:=arrayData[(lo+hi) **div** 2];

**repeat**

**while** (arrayData[lo]<mid) **do** inc(lo);

**while** (arrayData[hi]>mid) **do** dec(hi);

**if** lo<=hi **then** **begin**

n:=arrayData[hi];

arrayData[hi]:=arrayData[lo];

arrayData[lo]:=n;

inc(lo);

dec(hi);

**end**;

**until** lo>hi;

**if** hi>iLo **then** quickSort(arrayData,iLo,hi);

**if** lo<iHi **then** quickSort(arrayData,lo,iHi);

**end**;

**function** getNArom(atomN:integer; **const** connection:**array** **of** TConnBonds; **const** orderList:**array** **of** integer):integer;

**var**

i,n:integer;

**begin**

result:=0;

**for** i:=0 **to** connection[atomN].nb-1 **do** **begin**

n:=connection[atomN].bn[i];

**if** orderList[n]=0 **then** inc(result);

**end**;

**end**;

**function** calculateDummyValency(atomNo:integer; **const** connection:**array** **of** TConnBonds;

**const** bondOrder:**array** **of** integer):integer;

**var**

i,n,bondNo:integer;

**begin**

result:=0;

**for** i:=0 **to** connection[atomNo].nb-1 **do** **begin**

bondNo:=connection[atomNo].bn[i];

n:=bondOrder[bondNo];

**if** n=0 **then** n:=1; *//aromatic bond gives at least 1 in the valency*

result:=result+n;

**end**;

**end**;

**function** calculateCurrentValency(atomNo:integer; **const** connection:**array** **of** TConnBonds;

**const** bondOrder:**array** **of** integer):integer;

**var**

i,n,bondNo:integer;

**begin**

result:=0;

**for** i:=0 **to** connection[atomNo].nb-1 **do** **begin**

bondNo:=connection[atomNo].bn[i];

n:=bondOrder[bondNo];

result:=result+n;

**end**;

**end**;

**function** isHole(atomNo:integer; **const** valencyList,orderList:**array** **of** integer; **const** connection:**array** **of** TConnBonds):boolean;

**var**

j,n,aVal:integer;

**begin**

result:=false;

aVal:=0;

**for** j:=0 **to** connection[atomNo].nb-1 **do** **begin**

n:=connection[atomNo].bn[j];

aVal:=aVal+orderList[n];

**end**;

**if** aVal<valencyList[atomNo] **then** isHole:=true **else** **if** aVal>valencyList[atomNo] **then** **begin**

**raise** EAlgorithmError.create(format(BAD_VALENCY,[valencyList[atomNo],atomNo]));

**end**;

**end**;

**procedure** assignBonds(**const** valencyList, an1List, an2List:**array** **of** integer; **const** connection:**array** **of** TConnBonds;

**var** orderList*{,currentValency}*:**array** **of** integer);

**var**

i,j,n:integer;

deltaVal,bondNo,nArom,atomNo*{,secondAtom}*:integer;

addedList,accumulatedList:TInt32List;

**begin**

*//NArom = 1 - valency*

*//NArom=vlency - all first order*

addedList:=**nil**;

accumulatedList:=**nil**;

**try**

addedList:=TInt32List.Create;

addedList.Capacity:=high(valencyList)-low(valencyList)+1;

accumulatedList:=TInt32List.Create;

accumulatedList.Capacity:=addedList.Capacity;

**for** i:=low(connection) **to** high(connection) **do** addedList.Add(i);

**repeat**

**for** i:=0 **to** addedList.Count-1 **do** **begin**

atomNo:=addedList.AsLongint[i];

nArom:=getNArom(atomNo,connection,orderList);

deltaVal:=valencyList[atomNo]-calculateCurrentValency(atomNo,connection,orderList);

**if** (nArom=1) **and** (deltaVal>0) **then** **begin**

*//single aromatic bond*

**if** deltaVal>3 **then** **begin**

**raise** EAlgorithmError.create(format(BAD_VALENCY,[valencyList[atomNo],atomNo]));

**end**;

**for** j:=0 **to** connection[atomNo].nb-1 **do** **begin**

bondNo:=connection[atomNo].bn[j];

**if** orderList[bondNo]=0 **then** **begin**

*// if an1List[bondNo]=atomNo then secondAtom:=an2List[bondNo] else secondAtom:=an1List[bondNo];*

orderList[bondNo]:=deltaVal;

n:=an2List[bondNo];

**if** n=atomNo **then** accumulatedList.Add(an1List[bondNo]) **else** accumulatedList.Add(n);

break;

**end**;

**end**;

**end** **else** **if** (nArom=deltaVal) **then** **begin**

*//number of aromatic bonds is equal to valency*

**for** j:=0 **to** connection[atomNo].nb-1 **do** **begin**

bondNo:=connection[atomNo].bn[j];

**if** orderList[bondNo]=0 **then** **begin**

orderList[bondNo]:=1;

n:=an2List[bondNo];

**if** n=atomNo **then** accumulatedList.Add(an1List[bondNo]) **else** accumulatedList.Add(n);

**end**;

**end**;

**end**;

**end**;

addedList.Assign(accumulatedList);

accumulatedList.Clear;

**until** addedList.Count=0;

**finally**

addedList.free;

accumulatedList.Free;

**end**;

**end**;

**procedure** alternateBonds(**const** valencyList, an1List, an2List:**array** **of** integer; **const** connection:**array** **of** TConnBonds;

**var** orderList:**array** **of** integer);

**var**

currentList,accumulatedList,currentBondOrder,accumulatedBondOrder:TInt32List;

i,j,atomNo,bondNo,bOrder,deltaVal,atomSecond:integer;

twoAdded:boolean;

**begin**

currentList:=**nil**;

accumulatedList:=**nil**;

currentBondOrder:=**nil**;

accumulatedBondOrder:=**nil**;

**try**

currentList:=TInt32List.Create;

accumulatedList:=TInt32List.Create;

currentBondOrder:=TInt32List.Create;

accumulatedBondOrder:=TInt32List.Create;

**for** i:=low(orderList) **to** high(orderList) **do** **if** orderList[i]=0 **then** **begin**

currentList.Add(an1List[i]);

currentBondOrder.add(1);

break;

**end**;

**while** currentList.Count>0 **do** **begin**

**for** i:=0 **to** currentList.Count-1 **do** **begin**

atomNo:=currentList.AsLongint[i];

twoAdded:=false;

**for** j:=0 **to** connection[atomNo].nb-1 **do** **begin**

bondNo:=connection[atomNo].bn[j];

**if** orderList[bondNo]=0 **then** **begin**

**if** currentBondOrder.AsLongint[i]=2 **then** bOrder:=1 **else** bOrder:=2;

**if** (bOrder=2) **and** twoAdded **then** bOrder:=1;

**if** bOrder=2 **then** **begin** *//first atom remaining valencies check*

deltaVal:=valencyList[atomNo]-calculateDummyValency(atomNo,connection,orderList);

**if** deltaVal=0 **then** bOrder:=1;

**end**;

**if** an1List[bondNo]=atomNo **then** atomSecond:=an2List[bondNo] **else** atomSecond:=an1List[bondNo];

**if** bOrder=2 **then** **begin** *//second atom remaining valencies check*

deltaVal:=valencyList[atomSecond]-calculateDummyValency(atomSecond,connection,orderList);

**if** deltaVal=0 **then** bOrder:=1;

**end**;

orderList[bondNo]:=bOrder;

**if** bOrder=2 **then** twoAdded:=true;

accumulatedList.Add(atomSecond);

accumulatedBondOrder.Add(bOrder);

**end**;

**end**;

**end**;

currentList.Assign(accumulatedList);

accumulatedList.Clear;

currentBondOrder.Assign(accumulatedBondOrder);

accumulatedBondOrder.Clear;

**end**;

**finally**

currentList.Free;

accumulatedList.Free;

**end**;

**end**;

*//remove unassigned atoms*

**function** removeHole(atomNo:integer; **const** valencyList, an1List, an2List:**array** **of** integer;

**const** connection:**array** **of** TConnBonds; **var** orderList*{,currentValency}*:**array** **of** integer):boolean;

**var**

prevBond:**array** **of** integer;

currentList,accumulatedList:TInt32List;

i,j,secondAtom,firstAtom,endAtom,bondNo:integer;

doubleNeed:boolean;

**begin**

result:=false;

setLength(prevBond,high(valencyList)-low(valencyList)+1);

**for** i:=low(prevBond) **to** high(prevBond) **do** prevBond[i]:=-1;

prevBond[atomNo]:=high(longint);

currentList:=**nil**;

accumulatedList:=**nil**;

**try**

currentList:=TInt32List.Create;

accumulatedList:=TInt32List.Create;

currentList.Add(atomNo);

endAtom:=-1;

doubleNeed:=false;

**while** (currentList.Count>0) **and** (endAtom<0) **do** **begin**

**for** i:=0 **to** currentList.Count-1 **do** **begin**

firstAtom:=currentList.AsLongint[i];

**for** j:=0 **to** connection[firstAtom].nb-1 **do** **begin**

bondNo:=connection[firstAtom].bn[j];

**if** (doubleNeed **and** (orderList[bondNo]=2)) **or** ((**not** doubleNeed) **and** (orderList[bondNo]=1)) **then** **begin**

**if** an1List[bondNo]=firstAtom **then** secondAtom:=an2List[bondNo] **else** secondAtom:=an1List[bondNo];

**if** connection[secondAtom].nb<valencyList[secondAtom] **then** **begin**

**if** prevBond[secondAtom]<0 **then** **begin**

prevBond[secondAtom]:=bondNo;

accumulatedList.Add(secondAtom);

**if** isHole(secondAtom,valencyList,orderList,connection) **then** **begin**

**if** doubleNeed **then** **begin** *//connection through double-bond with hole-remove*

prevBond[secondAtom]:=-1;

accumulatedList.delete(accumulatedList.Count-1);

**end** **else** endAtom:=secondAtom;

**end**;

**end**;

**end**;

**end**;

**end**;

**end**;

**if** endAtom>=0 **then** currentList.Clear **else** currentList.Assign(accumulatedList);

accumulatedList.Clear;

doubleNeed:=**not** doubleNeed;

**end**;

**if** endAtom>=0 **then** **begin** *//re-alternate bonds*

result:=true;

**repeat**

bondNo:=prevBond[endAtom];

**if** bondNo<>high(longint) **then** **begin**

**if** orderList[bondNo]=1 **then** orderList[bondNo]:=2 **else** orderList[bondNo]:=1;

**if** endAtom=an1List[bondNo] **then** endAtom:=an2List[bondNo] **else** endAtom:=an1List[bondNo];

**end**;

**until** bondNo=high(longint);

**end**;

**finally**

currentList.Free;

accumulatedList.Free;

**end**;

**end**;

**function** kekulizeBondsInternal(**const** valencyList, an1List, an2List:**array** **of** integer;

**var** orderList:**array** **of** integer; **var** nHoles:integer):integer;

*//order list:on input - suggested orders, on output-calculated order*

*//return value-number of non-closed holes. 0 - holes are absent and kekulization was OK*

**var**

connection:**array** **of** TConnBonds;

i,j,n:integer;

**begin**

result:=0;

nHoles:=0;

*//initializing*

**if** (high(an1List)<>high(an2List)) **or** (high(an1List)<>high(orderList)) **then** exit;

setLength(connection,high(valencyList)-low(valencyList)+1);

n:=(high(valencyList)-low(valencyList)+1)*sizeof(TConnBonds);

**for** i:=low(connection) **to** high(connection) **do** **begin**

connection[i].nb:=0;

**for** j:=0 **to** CONN_MAX-1 **do** connection[i].bn[j]:=-1;

**end**;

*//current bonds-connection formation*

**for** i:=low(an1List) **to** high(an1List) **do** **begin**

n:=connection[an1List[i]].nb;

connection[an1List[i]].bn[n]:=i;

connection[an1List[i]].nb:=connection[an1List[i]].nb+1;

n:=connection[an2List[i]].nb;

connection[an2List[i]].bn[n]:=i;

connection[an2List[i]].nb:=connection[an2List[i]].nb+1;

**end**;

*//checking if number of bonds less or equal max val*

**for** i:=low(valencyList) **to** high(valencyList) **do** **if** valencyList[i]<connection[i].nb **then** **begin**

**raise** EAlgorithmError.create(format(BAD_NUMBER_OF_BONDS,[connection[i].nb,i,valencyList[i]]));

**end**;

*//prliminary run - assign all assignable bonds*

assignBonds(valencyList,an1List,an2List,connection,orderList);

**for** i:=low(valencyList) **to** high(valencyList) **do** **if** calculateDummyValency(i,connection,orderList)>valencyList[i] **then** **begin**

*//raise EAlgorithmError.create(format(BAD_NUMBER_OF_BONDS,[connection[i].nb,i,valencyList[i]]));*

result:=-1;

exit;

**end**;

*//alternate bonds*

alternateBonds(valencyList,an1List,an2List,connection,orderList);

**for** i:=low(valencyList) **to** high(valencyList) **do** **if** calculateDummyValency(i,connection,orderList)>valencyList[i] **then** **begin**

**raise** EAlgorithmError.create(format(BAD_NUMBER_OF_BONDS,[connection[i].nb,i,valencyList[i]]));

**end**;

*//make all non-alternated orders to 1*

**for** i:=low(orderList) **to** high(orderList) **do** **if** orderList[i]=0 **then** orderList[i]:=1;

*//unassigned atoms closing*

**for** i:=low(valencyList) **to** high(valencyList) **do** **if** isHole(i,valencyList,orderList,connection) **then** **begin**

**if** removeHole(i,valencyList,an1List,an2List,connection,orderList) **then** inc(nHoles);

**end**;

nHoles:=2*nHoles;

*//number of unclosed holes calculation*

**for** i:=low(valencyList) **to** high(valencyList) **do** **if** isHole(i,valencyList,orderList,connection) **then** **begin**

inc(result);

**end**;

nHoles:=nHoles+result;

**end**;

**function** kekulizeBonds(**const** valencyList:**array** **of** integer; **const** an1List,an2List:**array** **of** integer;

**var** orderList:**array** **of** integer; **var** nHoles,nPass:integer):integer;

**var**

i,n,n1,n2:integer;

an1ListTemp,an2ListTemp,bondIDList:**array** **of** integer;

orderStoreList:**array** **of** integer;

tryCount:integer;

**begin**

nPass:=1;

setLength(orderStoreList,high(orderList)-low(orderList)+1);

**for** i:=low(orderList) **to** high(orderList) **do** orderStoreList[i]:=orderList[i];

result:=kekulizeBondsInternal(valencyList,an1List,an2List,orderList,nHoles);

**if** result>0 **then** **begin**

setLength(an1ListTemp,high(an1List)-low(an1List)+1);

setLength(an2ListTemp,high(an2List)-low(an2List)+1);

setLength(bondIDList,high(orderList)-low(orderList)+1);

randomize;

**for** tryCount:=0 **to** TRY_MAX-1 **do** **begin**

inc(nPass);

**for** i:=low(orderList) **to** high(orderList) **do** **begin**

orderList[i]:=orderStoreList[i];

bondIDList[i]:=i;

**end**;

**for** i:=low(an1List) **to** high(an1List) **do** **begin**

an1ListTemp[i]:=an1List[i];

an2ListTemp[i]:=an2List[i];

**end**;

**for** i:=0 **to** 10*(high(an1List)-low(an1List)) **do** **begin**

n1:=random(high(an1List)-low(an1List));

n2:=random(high(an1List)-low(an1List));

**if** (n1<=high(an1List)) **and** (n2<=high(an1List)) **and** (n1<>n2) **then** **begin**

n:=an1ListTemp[n1];

an1ListTemp[n1]:=an1ListTemp[n2];

an1ListTemp[n2]:=n;

n:=an2ListTemp[n1];

an2ListTemp[n1]:=an2ListTemp[n2];

an2ListTemp[n2]:=n;

n:=orderList[n1];

orderList[n1]:=orderList[n2];

orderList[n2]:=n;

n:=bondIDList[n1];

bondIDList[n1]:=bondIDList[n2];

bondIDList[n2]:=n;

**end**;

**end**;

result:=kekulizeBondsInternal(valencyList,an1ListTemp,an2ListTemp,orderList,nHoles);

**if** result=0 **then** **begin**

break;

**end**;

**end**;

**end**;

**if** (high(bondIDList)-low(bondIDList)+1)=(high(orderList)-low(orderList)+1) **then** **begin**

*//restore initial numbering*

**for** i:=low(bondIDList) **to** high(bondIDList) **do** **begin**

n:=bondIDList[i];

orderStoreList[n]:=orderList[i];

**end**;

**for** i:=low(orderList) **to** high(orderList) **do** orderList[i]:=orderStoreList[i];

**end**;

**end**;

**function** restoreBondOrders(**const** valencyList:**array** **of** integer; **const** an1List,an2List:**array** **of** integer;

**var** orderList:**array** **of** integer; **var** nHoles,nPass:integer):integer;

**var**

connection:**array** **of** TConnBonds;

atomEnumerator:**array** **of** integer;

newValencyList,newAN1List,newAN2List,newOrderList,aromBond,orderEnumerator:**array** **of** integer;

ynAtoms,ynFlags,ynChain,ynBonds,chainStore:**array** **of** integer;

ynCount,chainLength,atom1,atom2,aromBondCount,chainStoreCount,bondOrder,bondNo:integer;

i,j,k,n,n1,n2,nAtoms,nBonds:integer;

**begin**

result:=0;

setLength(connection,high(valencyList)-low(valencyList)+1);

n:=(high(valencyList)-low(valencyList)+1)*sizeof(TConnBonds);

**for** i:=low(connection) **to** high(connection) **do** **begin**

connection[i].nb:=0;

**for** j:=0 **to** CONN_MAX-1 **do** connection[i].bn[j]:=-1;

**end**;

*//current bonds-connection formation*

**for** i:=low(an1List) **to** high(an1List) **do** **begin**

n:=connection[an1List[i]].nb;

connection[an1List[i]].bn[n]:=i;

connection[an1List[i]].nb:=connection[an1List[i]].nb+1;

n:=connection[an2List[i]].nb;

connection[an2List[i]].bn[n]:=i;

connection[an2List[i]].nb:=connection[an2List[i]].nb+1;

**end**;

setLength(ynFlags,high(valencyList)-low(valencyList)+1);

setLength(aromBond,high(valencyList)-low(valencyList)+1);

**for** i:=low(ynFlags) **to** high(ynFlags) **do** ynFlags[i]:=0;

setLength(ynChain,MAX_CHAIN);

setLength(ynBonds,MAX_CHAIN);

ynCount:=0;

chainStoreCount:=0;

aromBondCount:=0;

setLength(ynAtoms,high(valencyList)-low(valencyList)+1);

setLength(chainStore,2*(high(valencyList)-low(valencyList)+1)); *//the array also store chain length*

**for** i:=low(valencyList) **to** high(valencyList) **do** **if** (valencyList[i]=4) **and** (connection[i].nb=2) **and** (ynFlags[i]=0) **then** **begin**

chainLength:=findYnChain(i,valencyList,an1List,an2List,connection,ynChain,ynBonds,atom1,atom2);

**if** (chainLength **mod** 2)=0 **then** **begin**

*//even atom number-prepare for chain removing and nond formation*

**for** j:=0 **to** chainLength-1 **do** **begin**

ynFlags[ynChain[j]]:=1;

ynAtoms[low(ynAtoms)+ynCount]:=ynChain[j]; *//accumulate atoms to be deleted*

inc(ynCount);

**end**;

aromBond[aromBondCount]:=atom1;

inc(aromBondCount);

aromBond[aromBondCount]:=atom2;

inc(aromBondCount);

*//chain to alternate in future*

chainStore[chainStoreCount]:=chainLength+1; *//save number of elements*

inc(chainStoreCount);

**for** j:=0 **to** chainLength *{!! not -1 - bond 1 greate}* **do** **begin**

chainStore[chainStoreCount]:=ynBonds[j];

inc(chainStoreCount);

**end**;

**end** **else** **begin**

*//odd atoms-double-cumulene-double bond assignment*

**for** j:=0 **to** chainLength-1 **do** **begin**

**for** k:=0 **to** connection[ynChain[j]].nb-1 **do** **begin**

n:=connection[ynChain[j]].bn[k];

orderList[n]:=2;

**end**;

ynFlags[ynChain[j]]:=1;

**end**;

**end**;

**end**;

*//maximally freeing resources prior nex calculations*

setLength(ynChain,0);

ynChain:=**nil**;

setLength(ynBonds,0);

ynBonds:=**nil**;

setLength(connection,0);

connection:=**nil**;

**if** ynCount=0 **then** **begin**

*//only odd-length cumulenes or no cumulenes/triple bonds*

*//freeing resources*

setLength(ynAtoms,0);

ynAtoms:=**nil**;

setLength(ynFlags,0);

ynFlags:=**nil**;

setLength(aromBond,0);

aromBond:=**nil**;

result:=kekulizeBonds(valencyList,an1List,an2List,orderList,nHoles,nPass);

**end** **else** **begin**

*//even-length cumulenes or triple bonds*

setLength(atomEnumerator,high(valencyList)-low(valencyList)+1); *//old->new corresponence*

setLength(newValencyList,high(valencyList)-low(valencyList)+1-ynCount);

nAtoms:=0;

*//removing yn-atoms-create enumerator*

**if** ynCount>1 **then** quickSort(ynAtoms,0,ynCount-1);

**for** i:=low(valencyList) **to** high(valencyList) **do** **begin**

n:=findQuick(ynAtoms,ynCount,i);

**if** n<0 **then** **begin**

newValencyList[nAtoms]:=valencyList[i];

atomEnumerator[i]:=nAtoms;

inc(nAtoms);

**end** **else** atomEnumerator[i]:=-(n+1); *//zero index is started*

**end**;

nHoles:=-1;

nPass:=-1;

setLength(newValencyList,nAtoms); *//redim to new*

*//new bond list formation*

setLength(newOrderList,high(orderList)-low(orderList)+1);

setLength(newAn1List,high(orderList)-low(orderList)+1);

setLength(newAn2List,high(orderList)-low(orderList)+1);

setLength(orderEnumerator,high(orderList)-low(orderList)+1);

nBonds:=0;

**for** i:=low(orderList) **to** high(orderList) **do** **begin**

n1:=atomEnumerator[an1List[i]];

n2:=atomEnumerator[an2List[i]];

**if** (n1>=0) **and** (n2>=0) **then** **begin**

newOrderList[nBonds]:=orderList[i];

newAn1List[nBonds]:=n1;

newAn2List[nBonds]:=n2;

orderEnumerator[nBonds]:=i;

inc(nBonds);

**end**;

**end**;

setLength(orderEnumerator,nBonds); *//slice enumeration array to save boundary of dummy bonds addition*

*//dummy bonds addition*

**for** i:=0 **to** (aromBondCount **div** 2)-1 **do** **begin**

n1:=aromBond[2*i];

n2:=aromBond[2*i+1];

n1:=atomEnumerator[n1];

n2:=atomEnumerator[n2];

**if** (n1>=0) **and** (n2>=0) **then** **begin**

newOrderList[nBonds]:=0;

newAn1List[nBonds]:=n1;

newAn2List[nBonds]:=n2;

inc(nBonds);

**end** **else** **raise** EAlgorithmError.Create(LOGIC_ERROR);

**end**;

setLength(newAn1List,nBonds);

setLength(newAn2List,nBonds);

setLength(newOrderList,nBonds);

result:=kekulizeBonds(newValencyList,newAn1List,newAn2List,newOrderList,nHoles,nPass);

*//mapping bond order of existing bonds into old one*

**for** i:=low(orderEnumerator) **to** high(orderEnumerator) **do** **begin**

orderList[orderEnumerator[i]]:=newOrderList[i];

**end**;

*//alternate orders in kumulene\enyn chains*

n:=0;

**for** i:=high(orderEnumerator)+1 **to** high(newOrderList) **do** **begin**

chainLength:=chainStore[n];

inc(n);

bondOrder:=newOrderList[i];

**for** j:=0 **to** chainLength-1 **do** **begin**

bondNo:=chainStore[n];

inc(n);

orderList[bondNo]:=bondOrder;

**if** bondOrder=1 **then** bondOrder:=3 **else** **if** bondOrder=3 **then** bondOrder:=1;

**end**;

**end**;

**end**;

**end**;

**end**.
