# Supplementary material for: Data on generation of Kekulé structures for graphenes, graphynes, nanotubes and fullerenes and their aza-analogs
Source: Data Brief. 2018 Nov 1;21:1166–77. doi: 10.1016/j.dib.2018.10.128 (PMC6258249; doi:10.1016/j.dib.2018.10.128)
Supplement: Supplementary file 11 — Supplementary material [file mmc11.docx]

Table 3. Results of calculations for fullerenes from the library by Yoshida

| File name | Formula | Time (ms) | Avg. no. iterations | Max. no. iterations | Unmatched atom statistic |
| --- | --- | --- | --- | --- | --- |
| No.1-D5d.cc1 | C100 | 0.219 | 1.0 | 3 | 0:158, 2:557, 4:268, 6:17 |
| No.10-C1.cc1 | C100 | 0.141 | 1.1 | 3 | 0:153, 2:618, 4:213, 6:16 |
| No.100-C1.cc1 | C100 | 0.157 | 1.0 | 3 | 0:152, 2:638, 4:202, 6:8 |
| No.101-C1.cc1 | C100 | 0.157 | 1.0 | 2 | 0:154, 2:636, 4:194, 6:15, 8:1 |
| No.102-C1.cc1 | C100 | 0.250 | 1.0 | 3 | 0:156, 2:601, 4:226, 6:17 |
| No.103-C2.cc1 | C100 | 0.265 | 1.0 | 2 | 0:157, 2:631, 4:194, 6:18 |
| No.104-C1.cc1 | C100 | 0.156 | 1.0 | 2 | 0:136, 2:632, 4:220, 6:11, 8:1 |
| No.105-C1.cc1 | C100 | 0.125 | 1.1 | 3 | 0:159, 2:589, 4:229, 6:23 |
| No.106-C1.cc1 | C100 | 0.140 | 1.1 | 3 | 0:148, 2:656, 4:187, 6:9 |
| No.107-C1.cc1 | C100 | 0.140 | 1.0 | 3 | 0:157, 2:602, 4:226, 6:15 |
| No.108-C1.cc1 | C100 | 0.125 | 1.0 | 3 | 0:151, 2:636, 4:201, 6:11, 8:1 |
| No.109-C1.cc1 | C100 | 0.125 | 1.0 | 3 | 0:147, 2:637, 4:204, 6:12 |
| No.11-C1.cc1 | C100 | 0.093 | 1.0 | 2 | 0:157, 2:617, 4:213, 6:13 |
| No.110-C1.cc1 | C100 | 0.094 | 1.0 | 3 | 0:145, 2:601, 4:243, 6:11 |
| No.111-C1.cc1 | C100 | 0.078 | 1.0 | 3 | 0:138, 2:612, 4:231, 6:18, 8:1 |
| No.112-C1.cc1 | C100 | 0.078 | 1.0 | 3 | 0:156, 2:616, 4:219, 6:9 |
| No.113-C1.cc1 | C100 | 0.156 | 1.0 | 2 | 0:151, 2:640, 4:195, 6:13, 8:1 |
| No.114-C1.cc1 | C100 | 0.171 | 1.0 | 2 | 0:149, 2:634, 4:201, 6:16 |
| No.115-C2.cc1 | C100 | 0.250 | 1.0 | 3 | 0:163, 2:616, 4:201, 6:20 |
| No.116-C1.cc1 | C100 | 0.203 | 1.0 | 3 | 0:161, 2:618, 4:205, 6:16 |
| No.117-C2.cc1 | C100 | 0.172 | 1.0 | 3 | 0:171, 2:599, 4:216, 6:14 |
| No.118-C1.cc1 | C100 | 0.157 | 1.0 | 2 | 0:152, 2:630, 4:202, 6:15, 8:1 |
| No.119-C1.cc1 | C100 | 0.125 | 1.0 | 3 | 0:153, 2:647, 4:191, 6:9 |
| No.12-C1.cc1 | C100 | 0.109 | 1.0 | 3 | 0:146, 2:614, 4:224, 6:15, 8:1 |
| No.120-C1.cc1 | C100 | 0.140 | 1.0 | 2 | 0:152, 2:630, 4:193, 6:25 |
| No.121-C1.cc1 | C100 | 0.109 | 1.0 | 2 | 0:172, 2:607, 4:209, 6:12 |
| No.122-C1.cc1 | C100 | 0.094 | 1.0 | 3 | 0:170, 2:615, 4:199, 6:16 |
| No.123-C1.cc1 | C100 | 0.078 | 1.0 | 3 | 0:183, 2:613, 4:195, 6:9 |
| No.124-C1.cc1 | C100 | 0.078 | 1.1 | 2 | 0:169, 2:617, 4:199, 6:15 |
| No.125-C1.cc1 | C100 | 0.079 | 1.0 | 3 | 0:142, 2:620, 4:224, 6:14 |
| No.126-Cs.cc1 | C100 | 0.093 | 1.0 | 3 | 0:167, 2:621, 4:201, 6:11 |
| No.127-Cs.cc1 | C100 | 0.266 | 1.0 | 3 | 0:181, 2:624, 4:186, 6:9 |
| No.128-C1.cc1 | C100 | 0.203 | 1.0 | 3 | 0:149, 2:631, 4:205, 6:15 |
| No.129-C1.cc1 | C100 | 0.157 | 1.0 | 2 | 0:161, 2:640, 4:184, 6:15 |
| No.13-C2.cc1 | C100 | 0.125 | 1.0 | 2 | 0:134, 2:589, 4:260, 6:17 |
| No.130-C2.cc1 | C100 | 0.235 | 1.0 | 3 | 0:145, 2:627, 4:208, 6:18, 8:2 |
| No.131-C1.cc1 | C100 | 0.250 | 1.0 | 3 | 0:132, 2:657, 4:198, 6:13 |
| No.132-C2.cc1 | C100 | 0.203 | 1.0 | 2 | 0:159, 2:596, 4:225, 6:19, 8:1 |
| No.133-C1.cc1 | C100 | 0.140 | 1.0 | 3 | 0:158, 2:613, 4:215, 6:14 |
| No.134-C1.cc1 | C100 | 0.234 | 1.0 | 3 | 0:166, 2:597, 4:222, 6:15 |
| No.135-C1.cc1 | C100 | 0.265 | 1.0 | 3 | 0:148, 2:607, 4:228, 6:17 |
| No.136-C1.cc1 | C100 | 0.156 | 1.0 | 3 | 0:144, 2:630, 4:207, 6:18, 8:1 |
| No.137-C1.cc1 | C100 | 0.141 | 1.0 | 3 | 0:166, 2:606, 4:208, 6:18, 8:2 |
| No.138-C1.cc1 | C100 | 0.109 | 1.0 | 3 | 0:158, 2:612, 4:220, 6:10 |
| No.139-C2.cc1 | C100 | 0.250 | 1.0 | 3 | 0:136, 2:619, 4:235, 6:10 |
| No.14-Cs.cc1 | C100 | 0.266 | 1.1 | 3 | 0:149, 2:605, 4:228, 6:18 |
| No.140-C1.cc1 | C100 | 0.172 | 1.1 | 3 | 0:154, 2:640, 4:193, 6:13 |
| No.141-C1.cc1 | C100 | 0.140 | 1.0 | 2 | 0:149, 2:642, 4:198, 6:11 |
| No.142-C2.cc1 | C100 | 0.141 | 1.0 | 2 | 0:157, 2:632, 4:192, 6:17, 8:2 |
| No.143-C1.cc1 | C100 | 0.125 | 1.0 | 2 | 0:165, 2:623, 4:191, 6:20, 8:1 |
| No.144-C1.cc1 | C100 | 0.235 | 1.0 | 2 | 0:171, 2:622, 4:189, 6:18 |
| No.145-C1.cc1 | C100 | 0.188 | 1.0 | 2 | 0:149, 2:610, 4:224, 6:16, 8:1 |
| No.146-C1.cc1 | C100 | 0.141 | 1.0 | 2 | 0:131, 2:637, 4:218, 6:14 |
| No.147-C1.cc1 | C100 | 0.203 | 1.0 | 2 | 0:150, 2:627, 4:216, 6:7 |
| No.148-C1.cc1 | C100 | 0.266 | 1.0 | 3 | 0:170, 2:620, 4:196, 6:14 |
| No.149-C2.cc1 | C100 | 0.172 | 1.0 | 3 | 0:171, 2:629, 4:188, 6:12 |
| No.15-Cs.cc1 | C100 | 0.203 | 1.0 | 3 | 0:156, 2:617, 4:215, 6:11, 8:1 |
| No.150-Cs.cc1 | C100 | 0.203 | 1.0 | 3 | 0:167, 2:611, 4:213, 6:9 |
| No.151-C2.cc1 | C100 | 0.141 | 1.0 | 3 | 0:159, 2:620, 4:205, 6:16 |
| No.152-C1.cc1 | C100 | 0.250 | 1.0 | 3 | 0:158, 2:637, 4:193, 6:12 |
| No.153-C1.cc1 | C100 | 0.250 | 1.0 | 3 | 0:151, 2:614, 4:224, 6:11 |
| No.154-C1.cc1 | C100 | 0.172 | 1.0 | 3 | 0:166, 2:598, 4:221, 6:15 |
| No.155-C1.cc1 | C100 | 0.141 | 1.1 | 3 | 0:149, 2:661, 4:185, 6:5 |
| No.156-C1.cc1 | C100 | 0.156 | 1.0 | 3 | 0:153, 2:639, 4:198, 6:9, 8:1 |
| No.157-C1.cc1 | C100 | 0.188 | 1.0 | 3 | 0:169, 2:615, 4:201, 6:15 |
| No.158-C1.cc1 | C100 | 0.140 | 1.0 | 3 | 0:175, 2:622, 4:193, 6:10 |
| No.159-C2.cc1 | C100 | 0.109 | 1.0 | 3 | 0:146, 2:627, 4:210, 6:17 |
| No.16-C2.cc1 | C100 | 0.297 | 1.0 | 3 | 0:145, 2:589, 4:253, 6:13 |
| No.160-C1.cc1 | C100 | 0.266 | 1.0 | 3 | 0:152, 2:656, 4:180, 6:12 |
| No.161-C1.cc1 | C100 | 0.265 | 1.1 | 3 | 0:152, 2:637, 4:198, 6:13 |
| No.162-C1.cc1 | C100 | 0.266 | 1.0 | 2 | 0:171, 2:631, 4:184, 6:12, 8:2 |
| No.163-C1.cc1 | C100 | 0.218 | 1.0 | 3 | 0:158, 2:625, 4:204, 6:12, 8:1 |
| No.164-C1.cc1 | C100 | 0.141 | 1.0 | 3 | 0:155, 2:614, 4:218, 6:13 |
| No.165-C1.cc1 | C100 | 0.125 | 1.0 | 3 | 0:158, 2:630, 4:199, 6:13 |
| No.166-C1.cc1 | C100 | 0.109 | 1.0 | 3 | 0:135, 2:615, 4:229, 6:21 |
| No.167-C1.cc1 | C100 | 0.078 | 1.0 | 3 | 0:154, 2:640, 4:196, 6:10 |
| No.168-C1.cc1 | C100 | 0.078 | 1.0 | 3 | 0:163, 2:629, 4:199, 6:9 |
| No.169-C1.cc1 | C100 | 0.078 | 1.1 | 3 | 0:155, 2:634, 4:199, 6:12 |
| No.17-C1.cc1 | C100 | 0.078 | 1.0 | 2 | 0:138, 2:639, 4:204, 6:19 |
| No.170-C1.cc1 | C100 | 0.078 | 1.0 | 3 | 0:150, 2:608, 4:223, 6:19 |
| No.171-C1.cc1 | C100 | 0.204 | 1.0 | 3 | 0:153, 2:643, 4:193, 6:11 |
| No.172-C1.cc1 | C100 | 0.266 | 1.0 | 3 | 0:169, 2:621, 4:205, 6:5 |
| No.173-C1.cc1 | C100 | 0.265 | 1.0 | 3 | 0:179, 2:642, 4:173, 6:6 |
| No.174-C2.cc1 | C100 | 0.172 | 1.0 | 2 | 0:180, 2:634, 4:172, 6:14 |
| No.175-C1.cc1 | C100 | 0.141 | 1.0 | 2 | 0:164, 2:644, 4:182, 6:10 |
| No.176-C1.cc1 | C100 | 0.125 | 1.0 | 3 | 0:155, 2:658, 4:173, 6:14 |
| No.177-C1.cc1 | C100 | 0.093 | 1.1 | 3 | 0:145, 2:641, 4:206, 6:8 |
| No.178-C1.cc1 | C100 | 0.078 | 1.1 | 3 | 0:179, 2:614, 4:196, 6:11 |
| No.179-C1.cc1 | C100 | 0.078 | 1.1 | 3 | 0:144, 2:646, 4:198, 6:12 |
| No.18-C2.cc1 | C100 | 0.172 | 1.0 | 3 | 0:157, 2:606, 4:226, 6:10, 8:1 |
| No.180-C1.cc1 | C100 | 0.250 | 1.0 | 3 | 0:156, 2:632, 4:201, 6:11 |
| No.181-C1.cc1 | C100 | 0.250 | 1.0 | 3 | 0:160, 2:612, 4:211, 6:17 |
| No.182-C1.cc1 | C100 | 0.281 | 1.0 | 3 | 0:155, 2:646, 4:182, 6:17 |
| No.183-C1.cc1 | C100 | 0.250 | 1.0 | 3 | 0:161, 2:646, 4:179, 6:14 |
| No.184-C1.cc1 | C100 | 0.172 | 1.0 | 2 | 0:167, 2:618, 4:205, 6:10 |
| No.185-C1.cc1 | C100 | 0.140 | 1.0 | 3 | 0:140, 2:621, 4:221, 6:17, 8:1 |
| No.186-C1.cc1 | C100 | 0.110 | 1.0 | 3 | 0:159, 2:621, 4:209, 6:11 |
| No.187-C1.cc1 | C100 | 0.157 | 1.0 | 2 | 0:150, 2:628, 4:204, 6:18 |
| No.188-C1.cc1 | C100 | 0.266 | 1.0 | 3 | 0:143, 2:636, 4:209, 6:12 |
| No.189-C1.cc1 | C100 | 0.157 | 1.0 | 3 | 0:148, 2:608, 4:229, 6:15 |
| No.19-C1.cc1 | C100 | 0.172 | 1.0 | 3 | 0:143, 2:614, 4:227, 6:15, 8:1 |
| No.190-C2.cc1 | C100 | 0.156 | 1.0 | 3 | 0:123, 2:619, 4:237, 6:20, 8:1 |
| No.191-C1.cc1 | C100 | 0.250 | 1.0 | 3 | 0:141, 2:648, 4:203, 6:8 |
| No.192-C1.cc1 | C100 | 0.219 | 1.0 | 2 | 0:157, 2:625, 4:209, 6:9 |
| No.193-C1.cc1 | C100 | 0.156 | 1.0 | 3 | 0:175, 2:602, 4:208, 6:15 |
| No.194-C1.cc1 | C100 | 0.141 | 1.0 | 3 | 0:173, 2:617, 4:194, 6:15, 8:1 |
| No.195-C1.cc1 | C100 | 0.109 | 1.1 | 3 | 0:163, 2:649, 4:172, 6:16 |
| No.196-C1.cc1 | C100 | 0.078 | 1.0 | 2 | 0:162, 2:616, 4:208, 6:14 |
| No.197-C1.cc1 | C100 | 0.078 | 1.0 | 3 | 0:175, 2:627, 4:181, 6:17 |
| No.198-C1.cc1 | C100 | 0.109 | 1.0 | 3 | 0:147, 2:646, 4:197, 6:10 |
| No.199-C1.cc1 | C100 | 0.250 | 1.0 | 3 | 0:147, 2:621, 4:210, 6:22 |
| No.2-D2.cc1 | C100 | 0.156 | 1.0 | 4 | 0:125, 2:604, 4:245, 6:26 |
| No.20-C1.cc1 | C100 | 0.140 | 1.0 | 3 | 0:146, 2:620, 4:223, 6:11 |
| No.200-C1.cc1 | C100 | 0.110 | 1.0 | 3 | 0:154, 2:650, 4:188, 6:8 |
| No.201-C1.cc1 | C100 | 0.079 | 1.1 | 3 | 0:161, 2:639, 4:186, 6:14 |
| No.202-C1.cc1 | C100 | 0.078 | 1.0 | 2 | 0:153, 2:647, 4:184, 6:16 |
| No.203-C1.cc1 | C100 | 0.078 | 1.0 | 3 | 0:166, 2:640, 4:179, 6:15 |
| No.204-C1.cc1 | C100 | 0.079 | 1.0 | 2 | 0:185, 2:610, 4:194, 6:11 |
| No.205-C1.cc1 | C100 | 0.094 | 1.0 | 3 | 0:176, 2:599, 4:209, 6:16 |
| No.206-C1.cc1 | C100 | 0.093 | 1.0 | 3 | 0:159, 2:659, 4:176, 6:6 |
| No.207-C1.cc1 | C100 | 0.250 | 1.0 | 2 | 0:163, 2:621, 4:208, 6:8 |
| No.208-Cs.cc1 | C100 | 0.156 | 1.0 | 2 | 0:157, 2:633, 4:190, 6:19, 8:1 |
| No.209-Cs.cc1 | C100 | 0.125 | 1.0 | 3 | 0:163, 2:612, 4:211, 6:14 |
| No.21-C1.cc1 | C100 | 0.109 | 1.0 | 2 | 0:146, 2:596, 4:246, 6:12 |
| No.210-C1.cc1 | C100 | 0.172 | 1.1 | 3 | 0:164, 2:627, 4:189, 6:20 |
| No.211-C1.cc1 | C100 | 0.141 | 1.0 | 3 | 0:148, 2:627, 4:215, 6:10 |
| No.212-C2.cc1 | C100 | 0.125 | 1.0 | 3 | 0:154, 2:635, 4:204, 6:7 |
| No.213-C1.cc1 | C100 | 0.171 | 1.0 | 3 | 0:162, 2:646, 4:186, 6:6 |
| No.214-C1.cc1 | C100 | 0.203 | 1.0 | 3 | 0:131, 2:642, 4:215, 6:12 |
| No.215-C1.cc1 | C100 | 0.172 | 1.1 | 2 | 0:150, 2:655, 4:185, 6:10 |
| No.216-C1.cc1 | C100 | 0.141 | 1.0 | 3 | 0:162, 2:631, 4:194, 6:13 |
| No.217-C1.cc1 | C100 | 0.218 | 1.0 | 4 | 0:175, 2:645, 4:172, 6:8 |
| No.218-C1.cc1 | C100 | 0.250 | 1.0 | 3 | 0:162, 2:648, 4:184, 6:6 |
| No.219-C1.cc1 | C100 | 0.156 | 1.0 | 3 | 0:169, 2:645, 4:175, 6:11 |
| No.22-C1.cc1 | C100 | 0.125 | 1.0 | 3 | 0:128, 2:617, 4:241, 6:14 |
| No.220-C1.cc1 | C100 | 0.125 | 1.0 | 3 | 0:153, 2:635, 4:198, 6:12, 8:2 |
| No.221-C3.cc1 | C100 | 0.093 | 1.0 | 2 | 0:164, 2:647, 4:179, 6:10 |
| No.222-C1.cc1 | C100 | 0.078 | 1.0 | 3 | 0:163, 2:643, 4:181, 6:13 |
| No.223-C1.cc1 | C100 | 0.079 | 1.0 | 3 | 0:141, 2:641, 4:198, 6:20 |
| No.224-C1.cc1 | C100 | 0.078 | 1.0 | 3 | 0:155, 2:653, 4:183, 6:9 |
| No.225-C1.cc1 | C100 | 0.078 | 1.1 | 3 | 0:170, 2:624, 4:195, 6:11 |
| No.226-C1.cc1 | C100 | 0.079 | 1.0 | 3 | 0:172, 2:600, 4:214, 6:14 |
| No.227-C1.cc1 | C100 | 0.078 | 1.0 | 3 | 0:149, 2:649, 4:196, 6:6 |
| No.228-C1.cc1 | C100 | 0.093 | 1.1 | 3 | 0:163, 2:646, 4:183, 6:8 |
| No.229-C1.cc1 | C100 | 0.094 | 1.0 | 2 | 0:171, 2:647, 4:173, 6:9 |
| No.23-C2.cc1 | C100 | 0.078 | 1.0 | 2 | 0:152, 2:593, 4:237, 6:17, 8:1 |
| No.230-C1.cc1 | C100 | 0.078 | 1.0 | 3 | 0:110, 2:670, 4:210, 6:10 |
| No.231-C1.cc1 | C100 | 0.094 | 1.1 | 4 | 0:170, 2:648, 4:170, 6:11, 8:1 |
| No.232-C1.cc1 | C100 | 0.172 | 1.0 | 3 | 0:154, 2:644, 4:189, 6:12, 8:1 |
| No.233-C1.cc1 | C100 | 0.266 | 1.0 | 2 | 0:138, 2:657, 4:191, 6:14 |
| No.234-C1.cc1 | C100 | 0.157 | 1.1 | 3 | 0:143, 2:627, 4:217, 6:13 |
| No.235-C1.cc1 | C100 | 0.140 | 1.0 | 3 | 0:142, 2:639, 4:212, 6:7 |
| No.236-C1.cc1 | C100 | 0.109 | 1.0 | 3 | 0:155, 2:630, 4:203, 6:12 |
| No.237-C1.cc1 | C100 | 0.094 | 1.1 | 2 | 0:178, 2:593, 4:211, 6:18 |
| No.238-C1.cc1 | C100 | 0.094 | 1.1 | 3 | 0:148, 2:640, 4:204, 6:8 |
| No.239-C1.cc1 | C100 | 0.187 | 1.0 | 3 | 0:156, 2:628, 4:207, 6:8, 8:1 |
| No.24-C1.cc1 | C100 | 0.297 | 1.0 | 3 | 0:132, 2:597, 4:257, 6:14 |
| No.240-C1.cc1 | C100 | 0.250 | 1.0 | 3 | 0:169, 2:620, 4:199, 6:12 |
| No.241-C1.cc1 | C100 | 0.266 | 1.0 | 4 | 0:152, 2:648, 4:186, 6:14 |
| No.242-C1.cc1 | C100 | 0.156 | 1.0 | 3 | 0:186, 2:627, 4:176, 6:11 |
| No.243-C1.cc1 | C100 | 0.125 | 1.0 | 3 | 0:181, 2:611, 4:187, 6:21 |
| No.244-Cs.cc1 | C100 | 0.219 | 1.0 | 3 | 0:163, 2:622, 4:202, 6:12, 8:1 |
| No.245-C1.cc1 | C100 | 0.234 | 1.0 | 3 | 0:170, 2:623, 4:189, 6:18 |
| No.246-C1.cc1 | C100 | 0.157 | 1.1 | 3 | 0:160, 2:647, 4:181, 6:12 |
| No.247-C1.cc1 | C100 | 0.203 | 1.1 | 3 | 0:164, 2:654, 4:177, 6:5 |
| No.248-C1.cc1 | C100 | 0.219 | 1.0 | 3 | 0:172, 2:634, 4:187, 6:7 |
| No.249-C1.cc1 | C100 | 0.172 | 1.0 | 3 | 0:166, 2:647, 4:179, 6:7, 8:1 |
| No.25-Cs.cc1 | C100 | 0.141 | 1.0 | 3 | 0:133, 2:633, 4:218, 6:16 |
| No.250-C1.cc1 | C100 | 0.203 | 1.0 | 3 | 0:164, 2:634, 4:188, 6:14 |
| No.251-C1.cc1 | C100 | 0.265 | 1.1 | 3 | 0:174, 2:643, 4:172, 6:11 |
| No.252-Cs.cc1 | C100 | 0.266 | 1.0 | 3 | 0:150, 2:610, 4:225, 6:15 |
| No.253-C1.cc1 | C100 | 0.156 | 1.0 | 3 | 0:183, 2:617, 4:185, 6:15 |
| No.254-C1.cc1 | C100 | 0.125 | 1.0 | 3 | 0:173, 2:615, 4:203, 6:9 |
| No.255-C1.cc1 | C100 | 0.109 | 1.0 | 3 | 0:151, 2:646, 4:190, 6:13 |
| No.256-C1.cc1 | C100 | 0.094 | 1.0 | 2 | 0:175, 2:635, 4:180, 6:10 |
| No.257-C1.cc1 | C100 | 0.078 | 1.0 | 3 | 0:151, 2:652, 4:188, 6:9 |
| No.258-C1.cc1 | C100 | 0.094 | 1.0 | 3 | 0:178, 2:618, 4:187, 6:17 |
| No.259-C1.cc1 | C100 | 0.156 | 1.0 | 3 | 0:144, 2:633, 4:202, 6:21 |
| No.26-C1.cc1 | C100 | 0.250 | 1.0 | 3 | 0:158, 2:622, 4:203, 6:16, 8:1 |
| No.260-C1.cc1 | C100 | 0.219 | 1.0 | 2 | 0:193, 2:627, 4:169, 6:11 |
| No.261-C1.cc1 | C100 | 0.218 | 1.0 | 3 | 0:175, 2:630, 4:178, 6:17 |
| No.262-C1.cc1 | C100 | 0.250 | 1.0 | 2 | 0:151, 2:638, 4:190, 6:21 |
| No.263-C1.cc1 | C100 | 0.156 | 1.0 | 2 | 0:164, 2:613, 4:210, 6:13 |
| No.264-C1.cc1 | C100 | 0.125 | 1.0 | 2 | 0:166, 2:643, 4:173, 6:18 |
| No.265-C1.cc1 | C100 | 0.218 | 1.1 | 3 | 0:176, 2:644, 4:168, 6:12 |
| No.266-C3.cc1 | C100 | 0.187 | 1.0 | 3 | 0:161, 2:633, 4:186, 6:18, 8:2 |
| No.267-C1.cc1 | C100 | 0.203 | 1.0 | 2 | 0:144, 2:623, 4:223, 6:10 |
| No.268-C1.cc1 | C100 | 0.219 | 1.1 | 3 | 0:169, 2:615, 4:207, 6:9 |
| No.269-C1.cc1 | C100 | 0.265 | 1.0 | 3 | 0:153, 2:634, 4:200, 6:13 |
| No.27-C1.cc1 | C100 | 0.172 | 1.0 | 3 | 0:147, 2:659, 4:185, 6:9 |
| No.270-C1.cc1 | C100 | 0.156 | 1.0 | 2 | 0:157, 2:661, 4:171, 6:11 |
| No.271-C2.cc1 | C100 | 0.234 | 1.0 | 4 | 0:142, 2:630, 4:220, 6:8 |
| No.272-C1.cc1 | C100 | 0.250 | 1.0 | 3 | 0:179, 2:605, 4:195, 6:21 |
| No.273-C2.cc1 | C100 | 0.171 | 1.0 | 2 | 0:145, 2:630, 4:216, 6:9 |
| No.274-C1.cc1 | C100 | 0.141 | 1.0 | 3 | 0:158, 2:624, 4:203, 6:14, 8:1 |
| No.275-C1.cc1 | C100 | 0.109 | 1.0 | 4 | 0:166, 2:634, 4:180, 6:19, 8:1 |
| No.276-C1.cc1 | C100 | 0.218 | 1.0 | 3 | 0:161, 2:622, 4:198, 6:19 |
| No.277-C1.cc1 | C100 | 0.266 | 1.0 | 2 | 0:162, 2:651, 4:181, 6:6 |
| No.278-C1.cc1 | C100 | 0.172 | 1.0 | 3 | 0:158, 2:613, 4:209, 6:19, 8:1 |
| No.279-C1.cc1 | C100 | 0.140 | 1.0 | 2 | 0:167, 2:599, 4:219, 6:15 |
| No.28-C1.cc1 | C100 | 0.109 | 1.0 | 3 | 0:148, 2:648, 4:197, 6:7 |
| No.280-C1.cc1 | C100 | 0.094 | 1.0 | 3 | 0:157, 2:617, 4:213, 6:13 |
| No.281-C1.cc1 | C100 | 0.187 | 1.0 | 3 | 0:160, 2:627, 4:199, 6:14 |
| No.282-C1.cc1 | C100 | 0.265 | 1.0 | 3 | 0:141, 2:636, 4:201, 6:22 |
| No.283-C1.cc1 | C100 | 0.172 | 1.0 | 3 | 0:138, 2:651, 4:195, 6:15, 8:1 |
| No.284-C1.cc1 | C100 | 0.125 | 1.0 | 3 | 0:177, 2:623, 4:184, 6:16 |
| No.285-Cs.cc1 | C100 | 0.109 | 1.1 | 3 | 0:148, 2:593, 4:241, 6:18 |
| No.286-C1.cc1 | C100 | 0.094 | 1.0 | 3 | 0:152, 2:654, 4:179, 6:14, 8:1 |
| No.287-Cs.cc1 | C100 | 0.078 | 1.0 | 3 | 0:161, 2:640, 4:190, 6:9 |
| No.288-C1.cc1 | C100 | 0.078 | 1.0 | 2 | 0:169, 2:610, 4:203, 6:18 |
| No.289-C1.cc1 | C100 | 0.078 | 1.0 | 4 | 0:154, 2:612, 4:216, 6:18 |
| No.29-C1.cc1 | C100 | 0.078 | 1.0 | 3 | 0:152, 2:618, 4:220, 6:9, 8:1 |
| No.290-Cs.cc1 | C100 | 0.078 | 1.0 | 3 | 0:146, 2:620, 4:212, 6:22 |
| No.291-Cs.cc1 | C100 | 0.125 | 1.1 | 3 | 0:164, 2:602, 4:222, 6:10, 8:2 |
| No.292-C1.cc1 | C100 | 0.172 | 1.0 | 3 | 0:127, 2:651, 4:209, 6:13 |
| No.293-C2.cc1 | C100 | 0.188 | 1.0 | 4 | 0:143, 2:629, 4:214, 6:13, 8:1 |
| No.294-Cs.cc1 | C100 | 0.172 | 1.0 | 3 | 0:157, 2:644, 4:187, 6:12 |
| No.295-C2v.cc1 | C100 | 0.219 | 1.0 | 3 | 0:118, 2:616, 4:241, 6:25 |
| No.296-C1.cc1 | C100 | 0.218 | 1.1 | 4 | 0:161, 2:616, 4:210, 6:12, 8:1 |
| No.297-C1.cc1 | C100 | 0.188 | 1.0 | 2 | 0:162, 2:619, 4:200, 6:19 |
| No.298-C1.cc1 | C100 | 0.203 | 1.0 | 4 | 0:162, 2:612, 4:212, 6:14 |
| No.299-C1.cc1 | C100 | 0.250 | 1.0 | 3 | 0:165, 2:628, 4:200, 6:7 |
| No.3-C1.cc1 | C100 | 0.156 | 1.0 | 3 | 0:137, 2:620, 4:219, 6:24 |
| No.30-C1.cc1 | C100 | 0.156 | 1.0 | 2 | 0:156, 2:624, 4:198, 6:22 |
| No.300-C1.cc1 | C100 | 0.312 | 1.0 | 2 | 0:170, 2:630, 4:186, 6:14 |
| No.301-C1.cc1 | C100 | 0.234 | 1.0 | 3 | 0:164, 2:625, 4:198, 6:13 |
| No.302-C1.cc1 | C100 | 0.140 | 1.0 | 2 | 0:125, 2:645, 4:220, 6:10 |
| No.303-C1.cc1 | C100 | 0.234 | 1.0 | 3 | 0:145, 2:659, 4:184, 6:12 |
| No.304-C2.cc1 | C100 | 0.266 | 1.0 | 3 | 0:170, 2:638, 4:179, 6:13 |
| No.305-C1.cc1 | C100 | 0.156 | 1.0 | 3 | 0:154, 2:652, 4:176, 6:18 |
| No.306-C1.cc1 | C100 | 0.218 | 1.0 | 3 | 0:154, 2:621, 4:214, 6:10, 8:1 |
| No.307-C1.cc1 | C100 | 0.250 | 1.1 | 3 | 0:143, 2:627, 4:215, 6:15 |
| No.308-C1.cc1 | C100 | 0.172 | 1.1 | 3 | 0:152, 2:639, 4:193, 6:16 |
| No.309-C1.cc1 | C100 | 0.140 | 1.0 | 2 | 0:159, 2:617, 4:203, 6:21 |
| No.31-C1.cc1 | C100 | 0.110 | 1.0 | 2 | 0:134, 2:620, 4:233, 6:12, 8:1 |
| No.310-C2.cc1 | C100 | 0.094 | 1.0 | 3 | 0:177, 2:599, 4:206, 6:18 |
| No.311-C1.cc1 | C100 | 0.094 | 1.1 | 3 | 0:154, 2:615, 4:219, 6:12 |
| No.312-C1.cc1 | C100 | 0.203 | 1.1 | 3 | 0:165, 2:625, 4:205, 6:5 |
| No.313-C1.cc1 | C100 | 0.250 | 1.0 | 3 | 0:157, 2:633, 4:199, 6:11 |
| No.314-C1.cc1 | C100 | 0.156 | 1.1 | 3 | 0:154, 2:647, 4:180, 6:18, 8:1 |
| No.315-C1.cc1 | C100 | 0.140 | 1.0 | 3 | 0:152, 2:635, 4:197, 6:16 |
| No.316-C1.cc1 | C100 | 0.110 | 1.0 | 4 | 0:153, 2:647, 4:187, 6:13 |
| No.317-C1.cc1 | C100 | 0.172 | 1.0 | 3 | 0:159, 2:640, 4:193, 6:8 |
| No.318-C2.cc1 | C100 | 0.265 | 1.1 | 3 | 0:155, 2:611, 4:217, 6:17 |
| No.319-Cs.cc1 | C100 | 0.250 | 1.0 | 3 | 0:147, 2:663, 4:183, 6:7 |
| No.32-C1.cc1 | C100 | 0.156 | 1.0 | 3 | 0:160, 2:601, 4:230, 6:9 |
| No.320-C1.cc1 | C100 | 0.141 | 1.0 | 3 | 0:159, 2:619, 4:206, 6:16 |
| No.321-T.cc1 | C100 | 0.109 | 1.0 | 2 | 0:169, 2:619, 4:200, 6:11, 8:1 |
| No.322-C3.cc1 | C100 | 0.094 | 1.0 | 2 | 0:185, 2:622, 4:176, 6:17 |
| No.323-C1.cc1 | C100 | 0.094 | 1.1 | 3 | 0:175, 2:629, 4:187, 6:9 |
| No.324-C1.cc1 | C100 | 0.110 | 1.1 | 3 | 0:136, 2:635, 4:217, 6:12 |
| No.325-C1.cc1 | C100 | 0.093 | 1.1 | 3 | 0:172, 2:628, 4:191, 6:9 |
| No.326-C2.cc1 | C100 | 0.140 | 1.0 | 3 | 0:160, 2:648, 4:184, 6:8 |
| No.327-C1.cc1 | C100 | 0.125 | 1.0 | 3 | 0:170, 2:630, 4:176, 6:24 |
| No.328-C1.cc1 | C100 | 0.094 | 1.0 | 3 | 0:154, 2:624, 4:209, 6:13 |
| No.329-C1.cc1 | C100 | 0.094 | 1.1 | 3 | 0:157, 2:634, 4:190, 6:19 |
| No.33-C1.cc1 | C100 | 0.125 | 1.0 | 2 | 0:150, 2:646, 4:194, 6:10 |
| No.330-C2.cc1 | C100 | 0.219 | 1.1 | 3 | 0:175, 2:610, 4:203, 6:12 |
| No.331-C1.cc1 | C100 | 0.156 | 1.0 | 4 | 0:157, 2:643, 4:192, 6:8 |
| No.332-C1.cc1 | C100 | 0.125 | 1.0 | 3 | 0:144, 2:663, 4:187, 6:6 |
| No.333-C1.cc1 | C100 | 0.110 | 1.0 | 3 | 0:185, 2:621, 4:183, 6:11 |
| No.334-C1.cc1 | C100 | 0.187 | 1.0 | 3 | 0:166, 2:635, 4:188, 6:10, 8:1 |
| No.335-C1.cc1 | C100 | 0.250 | 1.0 | 3 | 0:157, 2:651, 4:179, 6:12, 8:1 |
| No.336-C1.cc1 | C100 | 0.219 | 1.1 | 3 | 0:164, 2:628, 4:203, 6:4, 8:1 |
| No.337-C1.cc1 | C100 | 0.282 | 1.1 | 4 | 0:170, 2:610, 4:206, 6:13, 8:1 |
| No.338-C2.cc1 | C100 | 0.218 | 1.0 | 4 | 0:152, 2:631, 4:200, 6:17 |
| No.339-C1.cc1 | C100 | 0.157 | 1.0 | 3 | 0:154, 2:634, 4:204, 6:8 |
| No.34-C2.cc1 | C100 | 0.141 | 1.0 | 2 | 0:136, 2:622, 4:226, 6:16 |
| No.340-C1.cc1 | C100 | 0.188 | 1.0 | 3 | 0:166, 2:645, 4:181, 6:8 |
| No.341-C1.cc1 | C100 | 0.218 | 1.0 | 3 | 0:150, 2:655, 4:182, 6:13 |
| No.342-C1.cc1 | C100 | 0.157 | 1.0 | 3 | 0:170, 2:632, 4:188, 6:10 |
| No.343-C2.cc1 | C100 | 0.203 | 1.1 | 3 | 0:161, 2:633, 4:197, 6:9 |
| No.344-C1.cc1 | C100 | 0.266 | 1.0 | 3 | 0:158, 2:633, 4:202, 6:7 |
| No.345-Cs.cc1 | C100 | 0.156 | 1.0 | 3 | 0:157, 2:636, 4:198, 6:8, 8:1 |
| No.346-C1.cc1 | C100 | 0.219 | 1.1 | 3 | 0:141, 2:640, 4:208, 6:11 |
| No.347-C2v.cc1 | C100 | 0.266 | 1.0 | 3 | 0:154, 2:599, 4:230, 6:17 |
| No.348-C1.cc1 | C100 | 0.203 | 1.1 | 3 | 0:173, 2:622, 4:193, 6:12 |
| No.349-C1.cc1 | C100 | 0.141 | 1.0 | 3 | 0:144, 2:649, 4:193, 6:14 |
| No.35-C2.cc1 | C100 | 0.235 | 1.0 | 3 | 0:136, 2:594, 4:253, 6:16, 8:1 |
| No.350-C2.cc1 | C100 | 0.250 | 1.1 | 4 | 0:183, 2:617, 4:189, 6:11 |
| No.351-C1.cc1 | C100 | 0.172 | 1.0 | 2 | 0:164, 2:608, 4:212, 6:16 |
| No.352-C1.cc1 | C100 | 0.125 | 1.0 | 2 | 0:172, 2:634, 4:182, 6:12 |
| No.353-C1.cc1 | C100 | 0.110 | 1.1 | 3 | 0:147, 2:664, 4:179, 6:9, 8:1 |
| No.354-Cs.cc1 | C100 | 0.094 | 1.0 | 3 | 0:176, 2:641, 4:172, 6:11 |
| No.355-C1.cc1 | C100 | 0.109 | 1.0 | 3 | 0:168, 2:611, 4:208, 6:13 |
| No.356-Cs.cc1 | C100 | 0.109 | 1.0 | 3 | 0:139, 2:635, 4:213, 6:13 |
| No.357-C1.cc1 | C100 | 0.094 | 1.0 | 2 | 0:191, 2:607, 4:197, 6:5 |
| No.358-C1.cc1 | C100 | 0.094 | 1.0 | 3 | 0:156, 2:649, 4:190, 6:5 |
| No.359-C1.cc1 | C100 | 0.110 | 1.0 | 2 | 0:152, 2:642, 4:197, 6:8, 8:1 |
| No.36-C2v.cc1 | C100 | 0.093 | 1.0 | 3 | 0:128, 2:604, 4:249, 6:18, 8:1 |
| No.360-C1.cc1 | C100 | 0.110 | 1.0 | 3 | 0:199, 2:614, 4:173, 6:14 |
| No.361-C1.cc1 | C100 | 0.093 | 1.0 | 2 | 0:164, 2:643, 4:182, 6:11 |
| No.362-C1.cc1 | C100 | 0.094 | 1.0 | 3 | 0:182, 2:615, 4:192, 6:11 |
| No.363-C1.cc1 | C100 | 0.094 | 1.0 | 3 | 0:182, 2:625, 4:182, 6:10, 8:1 |
| No.364-C2.cc1 | C100 | 0.094 | 1.0 | 2 | 0:182, 2:634, 4:174, 6:10 |
| No.365-C1.cc1 | C100 | 0.109 | 1.0 | 2 | 0:160, 2:637, 4:191, 6:11, 8:1 |
| No.366-C1.cc1 | C100 | 0.125 | 1.0 | 3 | 0:187, 2:631, 4:170, 6:11, 8:1 |
| No.367-C1.cc1 | C100 | 0.219 | 1.0 | 2 | 0:148, 2:653, 4:189, 6:9, 8:1 |
| No.368-C1.cc1 | C100 | 0.156 | 1.0 | 2 | 0:174, 2:623, 4:195, 6:8 |
| No.369-C1.cc1 | C100 | 0.125 | 1.0 | 3 | 0:167, 2:628, 4:193, 6:12 |
| No.37-C2.cc1 | C100 | 0.171 | 1.0 | 3 | 0:129, 2:599, 4:252, 6:20 |
| No.370-C1.cc1 | C100 | 0.219 | 1.0 | 3 | 0:182, 2:629, 4:172, 6:17 |
| No.371-C1.cc1 | C100 | 0.204 | 1.0 | 3 | 0:167, 2:636, 4:190, 6:7 |
| No.372-C2.cc1 | C100 | 0.156 | 1.0 | 3 | 0:163, 2:612, 4:211, 6:14 |
| No.373-C1.cc1 | C100 | 0.141 | 1.0 | 3 | 0:143, 2:656, 4:194, 6:7 |
| No.374-C1.cc1 | C100 | 0.110 | 1.0 | 3 | 0:184, 2:633, 4:174, 6:9 |
| No.375-C1.cc1 | C100 | 0.125 | 1.1 | 3 | 0:166, 2:628, 4:192, 6:14 |
| No.376-C2.cc1 | C100 | 0.187 | 1.0 | 3 | 0:145, 2:654, 4:191, 6:9, 8:1 |
| No.377-C1.cc1 | C100 | 0.219 | 1.0 | 3 | 0:173, 2:628, 4:188, 6:11 |
| No.378-C2.cc1 | C100 | 0.156 | 1.0 | 3 | 0:156, 2:642, 4:188, 6:14 |
| No.379-C1.cc1 | C100 | 0.265 | 1.0 | 3 | 0:171, 2:645, 4:173, 6:11 |
| No.38-Cs.cc1 | C100 | 0.266 | 1.0 | 3 | 0:168, 2:589, 4:228, 6:15 |
| No.380-C1.cc1 | C100 | 0.172 | 1.0 | 2 | 0:188, 2:611, 4:192, 6:9 |
| No.381-C2.cc1 | C100 | 0.140 | 1.0 | 3 | 0:176, 2:642, 4:167, 6:15 |
| No.382-C1.cc1 | C100 | 0.109 | 1.0 | 2 | 0:173, 2:637, 4:184, 6:6 |
| No.383-C1.cc1 | C100 | 0.110 | 1.0 | 3 | 0:161, 2:645, 4:183, 6:10, 8:1 |
| No.384-C1.cc1 | C100 | 0.093 | 1.0 | 4 | 0:166, 2:646, 4:178, 6:10 |
| No.385-C1.cc1 | C100 | 0.109 | 1.0 | 3 | 0:159, 2:652, 4:176, 6:13 |
| No.386-C1.cc1 | C100 | 0.094 | 1.0 | 3 | 0:166, 2:639, 4:183, 6:12 |
| No.387-C2.cc1 | C100 | 0.188 | 1.0 | 3 | 0:140, 2:655, 4:193, 6:12 |
| No.388-Cs.cc1 | C100 | 0.265 | 1.1 | 4 | 0:169, 2:634, 4:189, 6:8 |
| No.389-C1.cc1 | C100 | 0.219 | 1.0 | 4 | 0:147, 2:637, 4:207, 6:9 |
| No.39-Cs.cc1 | C100 | 0.156 | 1.0 | 3 | 0:140, 2:627, 4:216, 6:16, 8:1 |
| No.390-D2.cc1 | C100 | 0.250 | 1.0 | 2 | 0:154, 2:601, 4:222, 6:23 |
| No.391-C1.cc1 | C100 | 0.265 | 1.0 | 4 | 0:158, 2:641, 4:188, 6:13 |
| No.392-C2.cc1 | C100 | 0.281 | 1.0 | 3 | 0:137, 2:648, 4:202, 6:13 |
| No.393-C1.cc1 | C100 | 0.219 | 1.1 | 3 | 0:148, 2:645, 4:191, 6:16 |
| No.394-Cs.cc1 | C100 | 0.156 | 1.1 | 3 | 0:162, 2:617, 4:211, 6:9, 8:1 |
| No.395-D2d.cc1 | C100 | 0.125 | 1.0 | 3 | 0:162, 2:616, 4:210, 6:11, 8:1 |
| No.396-C2.cc1 | C100 | 0.187 | 1.0 | 3 | 0:149, 2:681, 4:164, 6:6 |
| No.397-C1.cc1 | C100 | 0.250 | 1.0 | 3 | 0:179, 2:633, 4:177, 6:10, 8:1 |
| No.398-C1.cc1 | C100 | 0.203 | 1.0 | 2 | 0:143, 2:669, 4:173, 6:15 |
| No.399-C1.cc1 | C100 | 0.188 | 1.0 | 2 | 0:176, 2:613, 4:201, 6:10 |
| No.4-C2.cc1 | C100 | 0.188 | 1.0 | 3 | 0:126, 2:618, 4:236, 6:20 |
| No.40-C1.cc1 | C100 | 0.188 | 1.0 | 3 | 0:181, 2:589, 4:219, 6:11 |
| No.400-C1.cc1 | C100 | 0.219 | 1.0 | 2 | 0:180, 2:611, 4:196, 6:13 |
| No.401-C2.cc1 | C100 | 0.172 | 1.1 | 3 | 0:149, 2:634, 4:200, 6:16, 8:1 |
| No.402-C2.cc1 | C100 | 0.219 | 1.0 | 3 | 0:180, 2:625, 4:190, 6:5 |
| No.403-C1.cc1 | C100 | 0.281 | 1.1 | 3 | 0:201, 2:600, 4:187, 6:12 |
| No.404-C1.cc1 | C100 | 0.266 | 1.1 | 3 | 0:165, 2:636, 4:185, 6:14 |
| No.405-C1.cc1 | C100 | 0.172 | 1.1 | 3 | 0:164, 2:628, 4:200, 6:8 |
| No.406-D2.cc1 | C100 | 0.172 | 1.0 | 3 | 0:151, 2:630, 4:209, 6:10 |
| No.407-C1.cc1 | C100 | 0.140 | 1.0 | 2 | 0:161, 2:639, 4:191, 6:9 |
| No.408-C1.cc1 | C100 | 0.125 | 1.0 | 2 | 0:170, 2:618, 4:202, 6:10 |
| No.409-C1.cc1 | C100 | 0.110 | 1.0 | 3 | 0:150, 2:638, 4:206, 6:6 |
| No.41-C2.cc1 | C100 | 0.094 | 1.0 | 2 | 0:145, 2:610, 4:227, 6:18 |
| No.410-C1.cc1 | C100 | 0.109 | 1.0 | 2 | 0:162, 2:638, 4:182, 6:18 |
| No.411-C2.cc1 | C100 | 0.094 | 1.0 | 2 | 0:163, 2:632, 4:190, 6:13, 8:2 |
| No.412-C1.cc1 | C100 | 0.110 | 1.0 | 3 | 0:179, 2:625, 4:186, 6:10 |
| No.413-C2.cc1 | C100 | 0.125 | 1.0 | 3 | 0:156, 2:629, 4:201, 6:14 |
| No.414-Cs.cc1 | C100 | 0.203 | 1.0 | 3 | 0:165, 2:657, 4:169, 6:8, 8:1 |
| No.415-C1.cc1 | C100 | 0.141 | 1.0 | 2 | 0:191, 2:648, 4:157, 6:4 |
| No.416-C1.cc1 | C100 | 0.125 | 1.0 | 3 | 0:167, 2:643, 4:177, 6:12, 8:1 |
| No.417-C2v.cc1 | C100 | 0.250 | 1.0 | 2 | 0:156, 2:617, 4:205, 6:22 |
| No.418-C1.cc1 | C100 | 0.250 | 1.0 | 3 | 0:154, 2:604, 4:227, 6:15 |
| No.419-C1.cc1 | C100 | 0.156 | 1.0 | 3 | 0:151, 2:644, 4:192, 6:13 |
| No.42-C1.cc1 | C100 | 0.156 | 1.0 | 3 | 0:164, 2:620, 4:202, 6:14 |
| No.420-C1.cc1 | C100 | 0.140 | 1.0 | 3 | 0:145, 2:634, 4:201, 6:19, 8:1 |
| No.421-C2.cc1 | C100 | 0.125 | 1.0 | 3 | 0:157, 2:634, 4:195, 6:14 |
| No.422-C2.cc1 | C100 | 0.109 | 1.0 | 3 | 0:170, 2:620, 4:193, 6:17 |
| No.423-C1.cc1 | C100 | 0.094 | 1.0 | 3 | 0:138, 2:656, 4:195, 6:11 |
| No.424-C1.cc1 | C100 | 0.172 | 1.0 | 2 | 0:170, 2:643, 4:178, 6:9 |
| No.425-C1.cc1 | C100 | 0.203 | 1.0 | 3 | 0:173, 2:653, 4:160, 6:14 |
| No.426-C1.cc1 | C100 | 0.156 | 1.0 | 3 | 0:159, 2:645, 4:187, 6:9 |
| No.427-C1.cc1 | C100 | 0.125 | 1.0 | 3 | 0:193, 2:606, 4:182, 6:19 |
| No.428-C1.cc1 | C100 | 0.093 | 1.0 | 3 | 0:163, 2:635, 4:188, 6:14 |
| No.429-C2.cc1 | C100 | 0.188 | 1.0 | 3 | 0:184, 2:639, 4:168, 6:9 |
| No.43-C1.cc1 | C100 | 0.250 | 1.0 | 2 | 0:173, 2:648, 4:173, 6:6 |
| No.430-C2.cc1 | C100 | 0.219 | 1.0 | 2 | 0:152, 2:640, 4:197, 6:10, 8:1 |
| No.431-D2.cc1 | C100 | 0.265 | 1.0 | 3 | 0:158, 2:641, 4:191, 6:10 |
| No.432-D2.cc1 | C100 | 0.172 | 1.0 | 2 | 0:151, 2:642, 4:193, 6:14 |
| No.433-C2.cc1 | C100 | 0.125 | 1.0 | 2 | 0:172, 2:624, 4:190, 6:14 |
| No.434-C1.cc1 | C100 | 0.235 | 1.0 | 3 | 0:165, 2:639, 4:189, 6:6, 8:1 |
| No.435-C2.cc1 | C100 | 0.203 | 1.0 | 3 | 0:182, 2:640, 4:164, 6:14 |
| No.436-C1.cc1 | C100 | 0.219 | 1.0 | 3 | 0:175, 2:634, 4:178, 6:13 |
| No.437-C1.cc1 | C100 | 0.219 | 1.0 | 2 | 0:153, 2:633, 4:196, 6:18 |
| No.438-D2.cc1 | C100 | 0.156 | 1.0 | 4 | 0:169, 2:610, 4:207, 6:13, 8:1 |
| No.439-C2.cc1 | C100 | 0.125 | 1.0 | 3 | 0:168, 2:613, 4:204, 6:15 |
| No.44-C1.cc1 | C100 | 0.094 | 1.0 | 3 | 0:144, 2:634, 4:205, 6:16, 8:1 |
| No.440-C2.cc1 | C100 | 0.094 | 1.0 | 3 | 0:191, 2:629, 4:163, 6:16, 8:1 |
| No.441-C1.cc1 | C100 | 0.093 | 1.0 | 2 | 0:171, 2:646, 4:177, 6:6 |
| No.442-C2.cc1 | C100 | 0.094 | 1.0 | 2 | 0:192, 2:629, 4:170, 6:9 |
| No.443-C2.cc1 | C100 | 0.094 | 1.0 | 3 | 0:153, 2:657, 4:181, 6:8, 8:1 |
| No.444-Cs.cc1 | C100 | 0.094 | 1.0 | 3 | 0:149, 2:650, 4:188, 6:13 |
| No.445-D2.cc1 | C100 | 0.094 | 1.0 | 2 | 0:140, 2:654, 4:186, 6:20 |
| No.446-C2.cc1 | C100 | 0.187 | 1.1 | 2 | 0:175, 2:617, 4:201, 6:7 |
| No.447-Cs.cc1 | C100 | 0.188 | 1.0 | 3 | 0:181, 2:631, 4:178, 6:10 |
| No.448-C2.cc1 | C100 | 0.234 | 1.0 | 3 | 0:181, 2:650, 4:161, 6:8 |
| No.449-D2.cc1 | C100 | 0.172 | 1.0 | 3 | 0:183, 2:635, 4:173, 6:9 |
| No.45-C1.cc1 | C100 | 0.140 | 1.0 | 4 | 0:128, 2:618, 4:232, 6:22 |
| No.450-D5.cc1 | C100 | 0.235 | 1.1 | 3 | 0:200, 2:637, 4:158, 6:5 |
| No.46-C1.cc1 | C100 | 0.266 | 1.1 | 4 | 0:154, 2:614, 4:213, 6:18, 8:1 |
| No.47-C1.cc1 | C100 | 0.156 | 1.0 | 2 | 0:144, 2:611, 4:222, 6:22, 8:1 |
| No.48-Cs.cc1 | C100 | 0.188 | 1.1 | 3 | 0:131, 2:636, 4:214, 6:18, 8:1 |
| No.49-C1.cc1 | C100 | 0.250 | 1.0 | 3 | 0:130, 2:645, 4:198, 6:27 |
| No.5-C2.cc1 | C100 | 0.156 | 1.0 | 3 | 0:148, 2:599, 4:231, 6:22 |
| No.50-C1.cc1 | C100 | 0.125 | 1.0 | 2 | 0:161, 2:647, 4:183, 6:9 |
| No.51-C1.cc1 | C100 | 0.109 | 1.0 | 3 | 0:168, 2:635, 4:180, 6:16, 8:1 |
| No.52-C1.cc1 | C100 | 0.109 | 1.0 | 3 | 0:148, 2:628, 4:212, 6:12 |
| No.53-C1.cc1 | C100 | 0.235 | 1.1 | 3 | 0:150, 2:635, 4:200, 6:15 |
| No.54-C1.cc1 | C100 | 0.265 | 1.0 | 4 | 0:148, 2:635, 4:207, 6:10 |
| No.55-C1.cc1 | C100 | 0.250 | 1.0 | 3 | 0:137, 2:603, 4:235, 6:24, 8:1 |
| No.56-C1.cc1 | C100 | 0.156 | 1.0 | 2 | 0:160, 2:626, 4:203, 6:11 |
| No.57-C1.cc1 | C100 | 0.141 | 1.0 | 3 | 0:164, 2:627, 4:193, 6:15, 8:1 |
| No.58-C1.cc1 | C100 | 0.109 | 1.0 | 3 | 0:150, 2:632, 4:205, 6:13 |
| No.59-C1.cc1 | C100 | 0.109 | 1.0 | 3 | 0:123, 2:647, 4:203, 6:27 |
| No.6-C1.cc1 | C100 | 0.172 | 1.0 | 4 | 0:136, 2:598, 4:249, 6:17 |
| No.60-C1.cc1 | C100 | 0.203 | 1.0 | 3 | 0:151, 2:615, 4:214, 6:20 |
| No.61-C1.cc1 | C100 | 0.218 | 1.0 | 3 | 0:148, 2:634, 4:205, 6:13 |
| No.62-C1.cc1 | C100 | 0.250 | 1.0 | 3 | 0:143, 2:629, 4:206, 6:21, 8:1 |
| No.63-C1.cc1 | C100 | 0.187 | 1.0 | 3 | 0:138, 2:625, 4:216, 6:20, 8:1 |
| No.64-Cs.cc1 | C100 | 0.219 | 1.0 | 3 | 0:128, 2:610, 4:242, 6:19, 8:1 |
| No.65-C1.cc1 | C100 | 0.156 | 1.0 | 3 | 0:133, 2:667, 4:183, 6:17 |
| No.66-C2.cc1 | C100 | 0.156 | 1.0 | 3 | 0:126, 2:640, 4:221, 6:13 |
| No.67-D2.cc1 | C100 | 0.188 | 1.0 | 3 | 0:164, 2:596, 4:223, 6:16, 8:1 |
| No.68-C1.cc1 | C100 | 0.219 | 1.0 | 3 | 0:149, 2:632, 4:206, 6:13 |
| No.69-C1.cc1 | C100 | 0.218 | 1.0 | 2 | 0:158, 2:621, 4:211, 6:8, 8:2 |
| No.7-C1.cc1 | C100 | 0.172 | 1.0 | 2 | 0:137, 2:628, 4:215, 6:20 |
| No.70-C1.cc1 | C100 | 0.250 | 1.0 | 2 | 0:161, 2:628, 4:196, 6:15 |
| No.71-C1.cc1 | C100 | 0.157 | 1.0 | 3 | 0:159, 2:655, 4:179, 6:7 |
| No.72-C1.cc1 | C100 | 0.125 | 1.0 | 3 | 0:165, 2:627, 4:184, 6:24 |
| No.73-C1.cc1 | C100 | 0.141 | 1.0 | 2 | 0:179, 2:592, 4:220, 6:9 |
| No.74-C1.cc1 | C100 | 0.125 | 1.0 | 3 | 0:155, 2:621, 4:211, 6:13 |
| No.75-C1.cc1 | C100 | 0.109 | 1.0 | 2 | 0:173, 2:629, 4:186, 6:12 |
| No.76-C1.cc1 | C100 | 0.109 | 1.0 | 3 | 0:173, 2:608, 4:208, 6:11 |
| No.77-C1.cc1 | C100 | 0.203 | 1.0 | 2 | 0:152, 2:639, 4:199, 6:10 |
| No.78-C1.cc1 | C100 | 0.187 | 1.0 | 3 | 0:144, 2:642, 4:194, 6:19, 8:1 |
| No.79-C1.cc1 | C100 | 0.156 | 1.0 | 3 | 0:181, 2:598, 4:207, 6:14 |
| No.8-C2.cc1 | C100 | 0.125 | 1.0 | 2 | 0:152, 2:609, 4:226, 6:13 |
| No.80-C1.cc1 | C100 | 0.156 | 1.0 | 3 | 0:144, 2:640, 4:202, 6:13, 8:1 |
| No.81-C1.cc1 | C100 | 0.282 | 1.0 | 3 | 0:157, 2:611, 4:221, 6:11 |
| No.82-C1.cc1 | C100 | 0.203 | 1.0 | 2 | 0:156, 2:630, 4:198, 6:16 |
| No.83-C1.cc1 | C100 | 0.141 | 1.0 | 2 | 0:138, 2:607, 4:231, 6:23, 8:1 |
| No.84-C1.cc1 | C100 | 0.250 | 1.1 | 4 | 0:164, 2:602, 4:212, 6:22 |
| No.85-Cs.cc1 | C100 | 0.250 | 1.0 | 3 | 0:141, 2:633, 4:212, 6:14 |
| No.86-C1.cc1 | C100 | 0.141 | 1.0 | 3 | 0:175, 2:616, 4:196, 6:13 |
| No.87-C1.cc1 | C100 | 0.235 | 1.0 | 4 | 0:178, 2:593, 4:215, 6:14 |
| No.88-C1.cc1 | C100 | 0.250 | 1.0 | 2 | 0:148, 2:630, 4:209, 6:13 |
| No.89-C1.cc1 | C100 | 0.219 | 1.0 | 2 | 0:156, 2:626, 4:196, 6:21, 8:1 |
| No.9-C1.cc1 | C100 | 0.250 | 1.0 | 2 | 0:163, 2:597, 4:224, 6:15, 8:1 |
| No.90-Cs.cc1 | C100 | 0.265 | 1.0 | 2 | 0:153, 2:601, 4:228, 6:18 |
| No.91-C1.cc1 | C100 | 0.203 | 1.0 | 3 | 0:136, 2:639, 4:209, 6:16 |
| No.92-C1.cc1 | C100 | 0.250 | 1.0 | 2 | 0:139, 2:616, 4:222, 6:23 |
| No.93-Cs.cc1 | C100 | 0.250 | 1.0 | 2 | 0:147, 2:635, 4:198, 6:20 |
| No.94-C2v.cc1 | C100 | 0.172 | 1.0 | 3 | 0:174, 2:617, 4:197, 6:12 |
| No.95-C1.cc1 | C100 | 0.140 | 1.0 | 2 | 0:152, 2:647, 4:186, 6:15 |
| No.96-C1.cc1 | C100 | 0.110 | 1.0 | 2 | 0:152, 2:642, 4:186, 6:18, 8:2 |
| No.97-C1.cc1 | C100 | 0.094 | 1.0 | 2 | 0:159, 2:617, 4:207, 6:17 |
| No.98-C2.cc1 | C100 | 0.110 | 1.0 | 3 | 0:135, 2:660, 4:189, 6:16 |
| No.99-C2.cc1 | C100 | 0.109 | 1.0 | 3 | 0:151, 2:627, 4:209, 6:13 |
| C180.cc1 | C180 | 0.187 | 1.2 | 5 | 0:81, 2:530, 4:327, 6:56, 8:6 |
| C240.cc1 | C240 | 0.266 | 1.2 | 4 | 0:91, 2:519, 4:298, 6:83, 8:9 |
| C260.cc1 | C260 | 0.297 | 1.2 | 5 | 0:73, 2:411, 4:375, 6:121, 8:19, 10:1 |
| C320.cc1 | C320 | 0.640 | 1.3 | 7 | 0:34, 2:318, 4:423, 6:187, 8:31, 10:7 |
| C500.cc1 | C500 | 1.078 | 1.5 | 8 | 0:13, 2:194, 4:383, 6:288, 8:102, 10:18, 12:1, 14:1 |
| C540.cc1 | C540 | 0.907 | 1.6 | 7 | 0:35, 2:229, 4:362, 6:257, 8:93, 10:21, 12:2, 14:1 |
| C720.cc1 | C720 | 1.203 | 1.7 | 10 | 0:4, 2:103, 4:292, 6:331, 8:190, 10:68, 12:12 |
| c20ih.cc1 | C20 | 0.063 | 1.0 | 1 | 0:705, 2:295 |
| c24d6d.cc1 | C24 | 0.094 | 1.0 | 1 | 0:490, 2:510 |
| c26d3h.cc1 | C26 | 0.079 | 1.0 | 1 | 0:459, 2:537, 4:4 |
| c28d2.cc1 | C28 | 0.094 | 1.0 | 1 | 0:430, 2:566, 4:4 |
| c28td.cc1 | C28 | 0.078 | 1.0 | 1 | 0:388, 2:609, 4:3 |
| c30-1.cc1 | C30 | 0.063 | 1.0 | 1 | 0:466, 2:523, 4:11 |
| c30-2.cc1 | C30 | 0.062 | 1.0 | 2 | 0:426, 2:563, 4:11 |
| c30-3.cc1 | C30 | 0.047 | 1.0 | 1 | 0:406, 2:592, 4:2 |
| No.1-C2.cc1 | C32 | 0.047 | 1.0 | 2 | 0:442, 2:550, 4:8 |
| No.2-D2.cc1 | C32 | 0.063 | 1.0 | 2 | 0:449, 2:530, 4:21 |
| No.3-D3d.cc1 | C32 | 0.078 | 1.0 | 1 | 0:415, 2:562, 4:23 |
| No.4-C2.cc1 | C32 | 0.093 | 1.0 | 1 | 0:427, 2:567, 4:6 |
| No.5-D3h.cc1 | C32 | 0.109 | 1.0 | 1 | 0:324, 2:661, 4:15 |
| No.6-D3.cc1 | C32 | 0.109 | 1.0 | 2 | 0:434, 2:558, 4:8 |
| No.1-C2.cc1 | C34 | 0.125 | 1.0 | 2 | 0:359, 2:621, 4:20 |
| No.2-Cs.cc1 | C34 | 0.109 | 1.0 | 2 | 0:419, 2:567, 4:14 |
| No.3-Cs.cc1 | C34 | 0.109 | 1.0 | 1 | 0:378, 2:599, 4:23 |
| No.4-C2.cc1 | C34 | 0.125 | 1.0 | 2 | 0:449, 2:537, 4:14 |
| No.5-C2.cc1 | C34 | 0.125 | 1.0 | 2 | 0:396, 2:591, 4:13 |
| No.6-C3v.cc1 | C34 | 0.109 | 1.0 | 2 | 0:360, 2:625, 4:15 |
| No.1-C2.cc1 | C36 | 0.094 | 1.0 | 2 | 0:327, 2:651, 4:22 |
| No.10-C2.cc1 | C36 | 0.125 | 1.0 | 1 | 0:362, 2:613, 4:25 |
| No.11-C2.cc1 | C36 | 0.140 | 1.0 | 2 | 0:374, 2:606, 4:20 |
| No.12-C2.cc1 | C36 | 0.094 | 1.0 | 2 | 0:402, 2:581, 4:17 |
| No.13-D3h.cc1 | C36 | 0.110 | 1.0 | 2 | 0:472, 2:516, 4:12 |
| No.14-D2d.cc1 | C36 | 0.094 | 1.0 | 2 | 0:457, 2:525, 4:18 |
| No.15-D6h.cc1 | C36 | 0.062 | 1.0 | 1 | 0:428, 2:562, 4:10 |
| No.2-D2.cc1 | C36 | 0.062 | 1.0 | 2 | 0:334, 2:630, 4:36 |
| No.3-C1.cc1 | C36 | 0.063 | 1.0 | 2 | 0:359, 2:609, 4:32 |
| No.4-Cs.cc1 | C36 | 0.094 | 1.0 | 2 | 0:361, 2:615, 4:24 |
| No.5-D2.cc1 | C36 | 0.078 | 1.0 | 2 | 0:309, 2:653, 4:38 |
| No.6-D2d.cc1 | C36 | 0.063 | 1.0 | 1 | 0:375, 2:594, 4:31 |
| No.7-C1.cc1 | C36 | 0.063 | 1.0 | 2 | 0:380, 2:605, 4:15 |
| No.8-Cs.cc1 | C36 | 0.078 | 1.0 | 2 | 0:376, 2:601, 4:23 |
| No.9-C2v.cc1 | C36 | 0.062 | 1.0 | 2 | 0:437, 2:550, 4:13 |
| No.1-C2.cc1 | C38 | 0.140 | 1.0 | 2 | 0:274, 2:679, 4:47 |
| No.10-C2.cc1 | C38 | 0.125 | 1.0 | 2 | 0:375, 2:595, 4:30 |
| No.11-C1.cc1 | C38 | 0.093 | 1.0 | 2 | 0:366, 2:604, 4:30 |
| No.12-C2v.cc1 | C38 | 0.125 | 1.0 | 1 | 0:305, 2:648, 4:47 |
| No.13-C2.cc1 | C38 | 0.140 | 1.0 | 2 | 0:370, 2:604, 4:26 |
| No.14-C1.cc1 | C38 | 0.125 | 1.0 | 2 | 0:392, 2:587, 4:21 |
| No.15-C2v.cc1 | C38 | 0.140 | 1.0 | 2 | 0:321, 2:651, 4:28 |
| No.16-C3v.cc1 | C38 | 0.125 | 1.0 | 2 | 0:396, 2:577, 4:27 |
| No.17-C2.cc1 | C38 | 0.140 | 1.0 | 2 | 0:405, 2:561, 4:34 |
| No.2-D3h.cc1 | C38 | 0.125 | 1.0 | 2 | 0:345, 2:592, 4:63 |
| No.3-C1.cc1 | C38 | 0.140 | 1.0 | 2 | 0:327, 2:638, 4:35 |
| No.4-C1.cc1 | C38 | 0.141 | 1.0 | 2 | 0:340, 2:628, 4:32 |
| No.5-C1.cc1 | C38 | 0.078 | 1.0 | 2 | 0:335, 2:630, 4:35 |
| No.6-C2.cc1 | C38 | 0.141 | 1.0 | 2 | 0:341, 2:635, 4:24 |
| No.7-C1.cc1 | C38 | 0.125 | 1.0 | 2 | 0:339, 2:617, 4:44 |
| No.8-C1.cc1 | C38 | 0.094 | 1.0 | 2 | 0:397, 2:573, 4:30 |
| No.9-D3.cc1 | C38 | 0.094 | 1.0 | 2 | 0:370, 2:607, 4:23 |
| No.1-D5d.cc1 | C40 | 0.125 | 1.0 | 2 | 0:266, 2:657, 4:77 |
| No.10-C1.cc1 | C40 | 0.110 | 1.0 | 2 | 0:320, 2:639, 4:41 |
| No.11-C2.cc1 | C40 | 0.141 | 1.0 | 2 | 0:294, 2:662, 4:44 |
| No.12-C1.cc1 | C40 | 0.094 | 1.0 | 2 | 0:295, 2:654, 4:51 |
| No.13-Cs.cc1 | C40 | 0.078 | 1.0 | 2 | 0:294, 2:665, 4:41 |
| No.14-Cs.cc1 | C40 | 0.093 | 1.0 | 2 | 0:355, 2:613, 4:32 |
| No.15-C2.cc1 | C40 | 0.141 | 1.0 | 2 | 0:376, 2:583, 4:41 |
| No.16-C2.cc1 | C40 | 0.125 | 1.0 | 2 | 0:388, 2:582, 4:30 |
| No.17-C1.cc1 | C40 | 0.125 | 1.0 | 2 | 0:345, 2:633, 4:22 |
| No.18-C2.cc1 | C40 | 0.141 | 1.0 | 2 | 0:313, 2:647, 4:40 |
| No.19-C2.cc1 | C40 | 0.125 | 1.0 | 2 | 0:343, 2:616, 4:41 |
| No.2-C2.cc1 | C40 | 0.125 | 1.0 | 2 | 0:286, 2:663, 4:51 |
| No.20-C3v.cc1 | C40 | 0.125 | 1.0 | 2 | 0:300, 2:659, 4:41 |
| No.21-C2.cc1 | C40 | 0.125 | 1.0 | 2 | 0:293, 2:671, 4:36 |
| No.22-C1.cc1 | C40 | 0.094 | 1.0 | 2 | 0:332, 2:625, 4:43 |
| No.23-C2.cc1 | C40 | 0.125 | 1.0 | 2 | 0:289, 2:644, 4:67 |
| No.24-Cs.cc1 | C40 | 0.141 | 1.0 | 2 | 0:334, 2:633, 4:33 |
| No.25-C2.cc1 | C40 | 0.125 | 1.0 | 2 | 0:341, 2:622, 4:37 |
| No.26-C1.cc1 | C40 | 0.141 | 1.0 | 2 | 0:340, 2:636, 4:24 |
| No.27-C2.cc1 | C40 | 0.141 | 1.0 | 2 | 0:339, 2:622, 4:39 |
| No.28-Cs.cc1 | C40 | 0.141 | 1.0 | 2 | 0:348, 2:618, 4:34 |
| No.29-C2.cc1 | C40 | 0.141 | 1.0 | 2 | 0:352, 2:621, 4:27 |
| No.3-D2.cc1 | C40 | 0.141 | 1.0 | 2 | 0:285, 2:644, 4:71 |
| No.30-C3.cc1 | C40 | 0.125 | 1.0 | 2 | 0:352, 2:623, 4:25 |
| No.31-Cs.cc1 | C40 | 0.125 | 1.0 | 2 | 0:406, 2:566, 4:28 |
| No.32-D2.cc1 | C40 | 0.125 | 1.0 | 3 | 0:318, 2:653, 4:29 |
| No.33-D2h.cc1 | C40 | 0.140 | 1.0 | 2 | 0:302, 2:648, 4:50 |
| No.34-C1.cc1 | C40 | 0.125 | 1.0 | 2 | 0:342, 2:615, 4:43 |
| No.35-C2.cc1 | C40 | 0.094 | 1.0 | 2 | 0:355, 2:602, 4:43 |
| No.36-C2.cc1 | C40 | 0.078 | 1.0 | 2 | 0:331, 2:627, 4:41, 6:1 |
| No.37-C2v.cc1 | C40 | 0.063 | 1.0 | 2 | 0:361, 2:601, 4:38 |
| No.38-D2.cc1 | C40 | 0.079 | 1.0 | 2 | 0:366, 2:614, 4:20 |
| No.39-D5d.cc1 | C40 | 0.078 | 1.0 | 1 | 0:397, 2:565, 4:38 |
| No.4-C1.cc1 | C40 | 0.125 | 1.0 | 2 | 0:321, 2:635, 4:44 |
| No.40-Td.cc1 | C40 | 0.125 | 1.0 | 3 | 0:458, 2:520, 4:22 |
| No.5-Cs.cc1 | C40 | 0.094 | 1.0 | 2 | 0:294, 2:668, 4:38 |
| No.6-C1.cc1 | C40 | 0.110 | 1.0 | 3 | 0:282, 2:669, 4:47, 6:2 |
| No.7-Cs.cc1 | C40 | 0.094 | 1.0 | 2 | 0:297, 2:653, 4:49, 6:1 |
| No.8-C2v.cc1 | C40 | 0.125 | 1.0 | 3 | 0:322, 2:636, 4:42 |
| No.9-C2.cc1 | C40 | 0.125 | 1.0 | 2 | 0:311, 2:650, 4:39 |
| No.1-C2.cc1 | C42 | 0.140 | 1.0 | 2 | 0:259, 2:670, 4:71 |
| No.10-C1.cc1 | C42 | 0.094 | 1.0 | 2 | 0:286, 2:663, 4:51 |
| No.11-Cs.cc1 | C42 | 0.110 | 1.0 | 2 | 0:288, 2:669, 4:43 |
| No.12-Cs.cc1 | C42 | 0.141 | 1.0 | 2 | 0:307, 2:654, 4:39 |
| No.13-C2v.cc1 | C42 | 0.141 | 1.0 | 2 | 0:331, 2:624, 4:44, 6:1 |
| No.14-C1.cc1 | C42 | 0.140 | 1.0 | 2 | 0:346, 2:615, 4:38, 6:1 |
| No.15-C1.cc1 | C42 | 0.109 | 1.0 | 2 | 0:268, 2:687, 4:45 |
| No.16-C2v.cc1 | C42 | 0.125 | 1.0 | 2 | 0:395, 2:576, 4:29 |
| No.17-C1.cc1 | C42 | 0.109 | 1.0 | 2 | 0:355, 2:607, 4:38 |
| No.18-C1.cc1 | C42 | 0.078 | 1.0 | 2 | 0:342, 2:624, 4:34 |
| No.19-Cs.cc1 | C42 | 0.078 | 1.0 | 2 | 0:322, 2:642, 4:36 |
| No.2-C1.cc1 | C42 | 0.062 | 1.0 | 2 | 0:262, 2:664, 4:73, 6:1 |
| No.20-C1.cc1 | C42 | 0.062 | 1.0 | 2 | 0:317, 2:646, 4:37 |
| No.21-C2v.cc1 | C42 | 0.109 | 1.0 | 2 | 0:333, 2:637, 4:30 |
| No.22-Cs.cc1 | C42 | 0.094 | 1.0 | 2 | 0:269, 2:661, 4:70 |
| No.23-C2.cc1 | C42 | 0.078 | 1.0 | 2 | 0:267, 2:679, 4:54 |
| No.24-C1.cc1 | C42 | 0.062 | 1.0 | 2 | 0:299, 2:644, 4:56, 6:1 |
| No.25-C1.cc1 | C42 | 0.063 | 1.0 | 2 | 0:276, 2:664, 4:60 |
| No.26-C1.cc1 | C42 | 0.046 | 1.0 | 2 | 0:303, 2:639, 4:58 |
| No.27-C2.cc1 | C42 | 0.063 | 1.0 | 2 | 0:256, 2:699, 4:45 |
| No.28-C2.cc1 | C42 | 0.063 | 1.0 | 2 | 0:294, 2:655, 4:51 |
| No.29-C1.cc1 | C42 | 0.063 | 1.0 | 2 | 0:327, 2:631, 4:42 |
| No.3-C1.cc1 | C42 | 0.062 | 1.0 | 2 | 0:292, 2:660, 4:48 |
| No.30-C1.cc1 | C42 | 0.047 | 1.0 | 2 | 0:287, 2:669, 4:44 |
| No.31-C2.cc1 | C42 | 0.094 | 1.0 | 2 | 0:318, 2:640, 4:42 |
| No.32-C1.cc1 | C42 | 0.125 | 1.0 | 2 | 0:330, 2:640, 4:30 |
| No.33-C1.cc1 | C42 | 0.140 | 1.0 | 2 | 0:319, 2:643, 4:38 |
| No.34-C1.cc1 | C42 | 0.110 | 1.0 | 2 | 0:337, 2:623, 4:40 |
| No.35-Cs.cc1 | C42 | 0.157 | 1.0 | 2 | 0:364, 2:598, 4:38 |
| No.36-C1.cc1 | C42 | 0.141 | 1.0 | 2 | 0:313, 2:648, 4:39 |
| No.37-C1.cc1 | C42 | 0.140 | 1.0 | 2 | 0:332, 2:611, 4:57 |
| No.38-C2.cc1 | C42 | 0.094 | 1.0 | 2 | 0:314, 2:633, 4:53 |
| No.39-C1.cc1 | C42 | 0.110 | 1.0 | 2 | 0:351, 2:619, 4:30 |
| No.4-C1.cc1 | C42 | 0.094 | 1.0 | 2 | 0:312, 2:631, 4:57 |
| No.40-C2.cc1 | C42 | 0.109 | 1.0 | 2 | 0:335, 2:636, 4:29 |
| No.41-C2.cc1 | C42 | 0.125 | 1.0 | 2 | 0:349, 2:613, 4:38 |
| No.42-Cs.cc1 | C42 | 0.125 | 1.0 | 2 | 0:312, 2:636, 4:52 |
| No.43-C2.cc1 | C42 | 0.140 | 1.0 | 2 | 0:299, 2:665, 4:36 |
| No.44-C1.cc1 | C42 | 0.141 | 1.0 | 2 | 0:327, 2:635, 4:38 |
| No.45-D3.cc1 | C42 | 0.094 | 1.0 | 2 | 0:350, 2:627, 4:23 |
| No.5-C2.cc1 | C42 | 0.078 | 1.0 | 2 | 0:322, 2:621, 4:55, 6:2 |
| No.6-C2v.cc1 | C42 | 0.110 | 1.0 | 2 | 0:257, 2:674, 4:69 |
| No.7-C2.cc1 | C42 | 0.094 | 1.0 | 2 | 0:298, 2:652, 4:50 |
| No.8-C1.cc1 | C42 | 0.078 | 1.0 | 2 | 0:266, 2:685, 4:47, 6:2 |
| No.9-C1.cc1 | C42 | 0.063 | 1.0 | 2 | 0:323, 2:636, 4:41 |
| No.1-C2.cc1 | C44 | 0.094 | 1.0 | 2 | 0:232, 2:685, 4:82, 6:1 |
| No.10-C1.cc1 | C44 | 0.078 | 1.0 | 2 | 0:282, 2:637, 4:81 |
| No.11-Cs.cc1 | C44 | 0.078 | 1.0 | 2 | 0:270, 2:655, 4:75 |
| No.12-C2.cc1 | C44 | 0.079 | 1.0 | 2 | 0:235, 2:676, 4:89 |
| No.13-C2v.cc1 | C44 | 0.078 | 1.0 | 3 | 0:259, 2:657, 4:84 |
| No.14-C2.cc1 | C44 | 0.141 | 1.0 | 2 | 0:264, 2:666, 4:70 |
| No.15-C1.cc1 | C44 | 0.140 | 1.0 | 2 | 0:273, 2:662, 4:64, 6:1 |
| No.16-C1.cc1 | C44 | 0.109 | 1.0 | 2 | 0:290, 2:654, 4:56 |
| No.17-C1.cc1 | C44 | 0.109 | 1.0 | 2 | 0:285, 2:670, 4:45 |
| No.18-C1.cc1 | C44 | 0.109 | 1.0 | 2 | 0:287, 2:647, 4:65, 6:1 |
| No.19-C1.cc1 | C44 | 0.094 | 1.0 | 2 | 0:247, 2:687, 4:66 |
| No.2-D2.cc1 | C44 | 0.078 | 1.0 | 2 | 0:228, 2:685, 4:87 |
| No.20-C2.cc1 | C44 | 0.078 | 1.0 | 2 | 0:291, 2:632, 4:77 |
| No.21-C1.cc1 | C44 | 0.062 | 1.0 | 2 | 0:276, 2:666, 4:58 |
| No.22-C1.cc1 | C44 | 0.047 | 1.0 | 2 | 0:329, 2:614, 4:57 |
| No.23-C1.cc1 | C44 | 0.047 | 1.0 | 2 | 0:319, 2:631, 4:50 |
| No.24-D2.cc1 | C44 | 0.062 | 1.0 | 2 | 0:387, 2:561, 4:52 |
| No.25-C1.cc1 | C44 | 0.141 | 1.0 | 2 | 0:313, 2:628, 4:59 |
| No.26-C1.cc1 | C44 | 0.141 | 1.0 | 2 | 0:308, 2:635, 4:57 |
| No.27-C1.cc1 | C44 | 0.093 | 1.0 | 2 | 0:314, 2:637, 4:49 |
| No.28-Cs.cc1 | C44 | 0.141 | 1.0 | 2 | 0:303, 2:637, 4:60 |
| No.29-C1.cc1 | C44 | 0.125 | 1.0 | 3 | 0:319, 2:633, 4:48 |
| No.3-D3d.cc1 | C44 | 0.141 | 1.0 | 2 | 0:293, 2:618, 4:89 |
| No.30-C1.cc1 | C44 | 0.109 | 1.0 | 2 | 0:320, 2:626, 4:53, 6:1 |
| No.31-C1.cc1 | C44 | 0.093 | 1.0 | 3 | 0:340, 2:605, 4:55 |
| No.32-C2.cc1 | C44 | 0.078 | 1.0 | 2 | 0:318, 2:627, 4:55 |
| No.33-Cs.cc1 | C44 | 0.078 | 1.0 | 2 | 0:287, 2:669, 4:43, 6:1 |
| No.34-C2.cc1 | C44 | 0.078 | 1.0 | 2 | 0:276, 2:663, 4:61 |
| No.35-D3.cc1 | C44 | 0.157 | 1.0 | 2 | 0:299, 2:639, 4:62 |
| No.36-C2.cc1 | C44 | 0.109 | 1.0 | 2 | 0:308, 2:649, 4:43 |
| No.37-D3h.cc1 | C44 | 0.078 | 1.0 | 3 | 0:262, 2:665, 4:73 |
| No.38-D3d.cc1 | C44 | 0.094 | 1.0 | 2 | 0:289, 2:621, 4:89, 6:1 |
| No.39-C2v.cc1 | C44 | 0.078 | 1.0 | 2 | 0:298, 2:637, 4:65 |
| No.4-C2.cc1 | C44 | 0.078 | 1.0 | 2 | 0:257, 2:687, 4:56 |
| No.40-C1.cc1 | C44 | 0.125 | 1.0 | 2 | 0:299, 2:635, 4:66 |
| No.41-C1.cc1 | C44 | 0.140 | 1.0 | 2 | 0:274, 2:653, 4:73 |
| No.42-C1.cc1 | C44 | 0.125 | 1.0 | 2 | 0:298, 2:648, 4:54 |
| No.43-C1.cc1 | C44 | 0.140 | 1.0 | 2 | 0:272, 2:656, 4:72 |
| No.44-C2.cc1 | C44 | 0.140 | 1.0 | 2 | 0:277, 2:649, 4:74 |
| No.45-C2.cc1 | C44 | 0.156 | 1.0 | 2 | 0:251, 2:670, 4:79 |
| No.46-C2.cc1 | C44 | 0.110 | 1.0 | 2 | 0:292, 2:663, 4:45 |
| No.47-C1.cc1 | C44 | 0.110 | 1.0 | 2 | 0:330, 2:618, 4:52 |
| No.48-C1.cc1 | C44 | 0.110 | 1.0 | 2 | 0:281, 2:662, 4:57 |
| No.49-C2.cc1 | C44 | 0.110 | 1.0 | 2 | 0:282, 2:667, 4:51 |
| No.5-C2.cc1 | C44 | 0.110 | 1.0 | 2 | 0:283, 2:656, 4:61 |
| No.50-C1.cc1 | C44 | 0.141 | 1.0 | 2 | 0:307, 2:651, 4:42 |
| No.51-C1.cc1 | C44 | 0.141 | 1.0 | 2 | 0:318, 2:621, 4:61 |
| No.52-C1.cc1 | C44 | 0.110 | 1.0 | 2 | 0:348, 2:605, 4:47 |
| No.53-C1.cc1 | C44 | 0.094 | 1.0 | 2 | 0:292, 2:658, 4:50 |
| No.54-Cs.cc1 | C44 | 0.110 | 1.0 | 2 | 0:311, 2:649, 4:40 |
| No.55-C2v.cc1 | C44 | 0.125 | 1.0 | 2 | 0:320, 2:629, 4:51 |
| No.56-C1.cc1 | C44 | 0.140 | 1.0 | 2 | 0:310, 2:637, 4:53 |
| No.57-C1.cc1 | C44 | 0.172 | 1.0 | 2 | 0:293, 2:658, 4:49 |
| No.58-C1.cc1 | C44 | 0.141 | 1.0 | 2 | 0:285, 2:671, 4:42, 6:2 |
| No.59-C1.cc1 | C44 | 0.140 | 1.0 | 2 | 0:321, 2:628, 4:51 |
| No.6-C2.cc1 | C44 | 0.109 | 1.0 | 2 | 0:294, 2:640, 4:66 |
| No.60-C1.cc1 | C44 | 0.094 | 1.0 | 3 | 0:315, 2:644, 4:41 |
| No.61-C2.cc1 | C44 | 0.141 | 1.0 | 2 | 0:280, 2:684, 4:36 |
| No.62-C1.cc1 | C44 | 0.140 | 1.0 | 2 | 0:283, 2:672, 4:45 |
| No.63-C1.cc1 | C44 | 0.109 | 1.0 | 2 | 0:322, 2:641, 4:37 |
| No.64-C1.cc1 | C44 | 0.141 | 1.0 | 2 | 0:310, 2:651, 4:39 |
| No.65-C1.cc1 | C44 | 0.141 | 1.0 | 2 | 0:299, 2:639, 4:62 |
| No.66-C2.cc1 | C44 | 0.157 | 1.0 | 2 | 0:235, 2:693, 4:72 |
| No.67-C1.cc1 | C44 | 0.187 | 1.0 | 2 | 0:294, 2:660, 4:46 |
| No.68-C2.cc1 | C44 | 0.094 | 1.0 | 2 | 0:349, 2:609, 4:42 |
| No.69-C1.cc1 | C44 | 0.156 | 1.0 | 2 | 0:317, 2:645, 4:38 |
| No.7-C1.cc1 | C44 | 0.141 | 1.0 | 2 | 0:286, 2:654, 4:60 |
| No.70-Cs.cc1 | C44 | 0.141 | 1.0 | 2 | 0:320, 2:645, 4:35 |
| No.71-Cs.cc1 | C44 | 0.125 | 1.0 | 2 | 0:283, 2:657, 4:58, 6:2 |
| No.72-D3h.cc1 | C44 | 0.110 | 1.0 | 2 | 0:359, 2:607, 4:34 |
| No.73-T.cc1 | C44 | 0.140 | 1.0 | 2 | 0:296, 2:664, 4:40 |
| No.74-C2.cc1 | C44 | 0.156 | 1.0 | 2 | 0:301, 2:643, 4:56 |
| No.75-D2.cc1 | C44 | 0.141 | 1.0 | 2 | 0:342, 2:623, 4:35 |
| No.76-C2.cc1 | C44 | 0.110 | 1.0 | 2 | 0:331, 2:626, 4:43 |
| No.77-C1.cc1 | C44 | 0.093 | 1.0 | 2 | 0:299, 2:655, 4:46 |
| No.78-C1.cc1 | C44 | 0.125 | 1.0 | 2 | 0:315, 2:650, 4:35 |
| No.79-C2.cc1 | C44 | 0.125 | 1.0 | 2 | 0:290, 2:662, 4:48 |
| No.8-C1.cc1 | C44 | 0.140 | 1.0 | 2 | 0:274, 2:660, 4:66 |
| No.80-D3.cc1 | C44 | 0.156 | 1.0 | 2 | 0:316, 2:638, 4:46 |
| No.81-C2.cc1 | C44 | 0.157 | 1.0 | 2 | 0:293, 2:648, 4:59 |
| No.82-S4.cc1 | C44 | 0.141 | 1.0 | 2 | 0:286, 2:672, 4:42 |
| No.83-D2.cc1 | C44 | 0.109 | 1.0 | 3 | 0:263, 2:690, 4:47 |
| No.84-Cs.cc1 | C44 | 0.094 | 1.0 | 2 | 0:294, 2:660, 4:46 |
| No.85-D2.cc1 | C44 | 0.078 | 1.0 | 2 | 0:283, 2:652, 4:65 |
| No.86-D3d.cc1 | C44 | 0.078 | 1.0 | 2 | 0:273, 2:632, 4:95 |
| No.87-C2.cc1 | C44 | 0.094 | 1.0 | 2 | 0:362, 2:606, 4:32 |
| No.88-C1.cc1 | C44 | 0.140 | 1.0 | 2 | 0:288, 2:676, 4:36 |
| No.89-D2.cc1 | C44 | 0.156 | 1.0 | 2 | 0:354, 2:613, 4:33 |
| No.9-C1.cc1 | C44 | 0.125 | 1.0 | 2 | 0:241, 2:666, 4:93 |
| No.1-C2.cc1 | C46 | 0.157 | 1.0 | 3 | 0:233, 2:654, 4:111, 6:2 |
| No.10-Cs.cc1 | C46 | 0.156 | 1.0 | 2 | 0:274, 2:639, 4:86, 6:1 |
| No.100-C1.cc1 | C46 | 0.141 | 1.0 | 2 | 0:288, 2:650, 4:62 |
| No.101-C1.cc1 | C46 | 0.125 | 1.0 | 2 | 0:286, 2:656, 4:58 |
| No.102-C1.cc1 | C46 | 0.141 | 1.0 | 2 | 0:300, 2:642, 4:58 |
| No.103-C1.cc1 | C46 | 0.110 | 1.0 | 2 | 0:330, 2:640, 4:29, 6:1 |
| No.104-C2.cc1 | C46 | 0.140 | 1.0 | 2 | 0:326, 2:623, 4:51 |
| No.105-C1.cc1 | C46 | 0.140 | 1.0 | 2 | 0:278, 2:650, 4:72 |
| No.106-Cs.cc1 | C46 | 0.157 | 1.0 | 2 | 0:315, 2:634, 4:51 |
| No.107-Cs.cc1 | C46 | 0.094 | 1.0 | 2 | 0:321, 2:639, 4:40 |
| No.108-Cs.cc1 | C46 | 0.125 | 1.0 | 2 | 0:315, 2:643, 4:42 |
| No.109-C2.cc1 | C46 | 0.141 | 1.0 | 2 | 0:322, 2:646, 4:32 |
| No.11-Cs.cc1 | C46 | 0.156 | 1.0 | 2 | 0:238, 2:661, 4:100, 6:1 |
| No.110-C1.cc1 | C46 | 0.110 | 1.0 | 3 | 0:295, 2:668, 4:37 |
| No.111-C1.cc1 | C46 | 0.079 | 1.0 | 2 | 0:298, 2:654, 4:48 |
| No.112-C2.cc1 | C46 | 0.078 | 1.0 | 2 | 0:283, 2:667, 4:50 |
| No.113-C2.cc1 | C46 | 0.110 | 1.0 | 2 | 0:238, 2:691, 4:71 |
| No.114-C1.cc1 | C46 | 0.125 | 1.0 | 2 | 0:297, 2:676, 4:27 |
| No.115-C3.cc1 | C46 | 0.188 | 1.0 | 2 | 0:287, 2:675, 4:38 |
| No.116-C2.cc1 | C46 | 0.140 | 1.0 | 2 | 0:319, 2:643, 4:38 |
| No.12-C2.cc1 | C46 | 0.141 | 1.0 | 2 | 0:317, 2:614, 4:68, 6:1 |
| No.13-Cs.cc1 | C46 | 0.141 | 1.0 | 3 | 0:287, 2:634, 4:79 |
| No.14-C1.cc1 | C46 | 0.109 | 1.0 | 2 | 0:269, 2:645, 4:86 |
| No.15-C1.cc1 | C46 | 0.109 | 1.0 | 2 | 0:243, 2:667, 4:88, 6:2 |
| No.16-C1.cc1 | C46 | 0.110 | 1.0 | 2 | 0:281, 2:650, 4:68, 6:1 |
| No.17-C1.cc1 | C46 | 0.141 | 1.0 | 2 | 0:262, 2:660, 4:78 |
| No.18-C1.cc1 | C46 | 0.093 | 1.0 | 2 | 0:308, 2:606, 4:85, 6:1 |
| No.19-C1.cc1 | C46 | 0.109 | 1.0 | 2 | 0:234, 2:680, 4:86 |
| No.2-Cs.cc1 | C46 | 0.141 | 1.0 | 3 | 0:294, 2:651, 4:55 |
| No.20-C2.cc1 | C46 | 0.141 | 1.0 | 2 | 0:229, 2:689, 4:81, 6:1 |
| No.21-C1.cc1 | C46 | 0.125 | 1.0 | 2 | 0:244, 2:662, 4:94 |
| No.22-C2.cc1 | C46 | 0.094 | 1.0 | 3 | 0:332, 2:606, 4:62 |
| No.23-C1.cc1 | C46 | 0.078 | 1.0 | 2 | 0:309, 2:629, 4:61, 6:1 |
| No.24-C1.cc1 | C46 | 0.078 | 1.0 | 2 | 0:305, 2:649, 4:46 |
| No.25-C1.cc1 | C46 | 0.062 | 1.0 | 3 | 0:321, 2:614, 4:65 |
| No.26-C1.cc1 | C46 | 0.063 | 1.0 | 2 | 0:315, 2:625, 4:59, 6:1 |
| No.27-C1.cc1 | C46 | 0.078 | 1.0 | 2 | 0:262, 2:681, 4:55, 6:2 |
| No.28-Cs.cc1 | C46 | 0.156 | 1.0 | 2 | 0:296, 2:623, 4:81 |
| No.29-C1.cc1 | C46 | 0.141 | 1.0 | 2 | 0:288, 2:645, 4:66, 6:1 |
| No.3-C1.cc1 | C46 | 0.141 | 1.0 | 3 | 0:250, 2:667, 4:83 |
| No.30-C1.cc1 | C46 | 0.109 | 1.0 | 2 | 0:292, 2:626, 4:82 |
| No.31-C1.cc1 | C46 | 0.094 | 1.0 | 2 | 0:286, 2:659, 4:54, 6:1 |
| No.32-C2.cc1 | C46 | 0.078 | 1.0 | 2 | 0:303, 2:643, 4:54 |
| No.33-Cs.cc1 | C46 | 0.078 | 1.0 | 2 | 0:255, 2:688, 4:56, 6:1 |
| No.34-C1.cc1 | C46 | 0.141 | 1.0 | 3 | 0:267, 2:674, 4:59 |
| No.35-C1.cc1 | C46 | 0.125 | 1.0 | 2 | 0:296, 2:658, 4:46 |
| No.36-C1.cc1 | C46 | 0.094 | 1.0 | 2 | 0:292, 2:652, 4:56 |
| No.37-C1.cc1 | C46 | 0.078 | 1.0 | 2 | 0:281, 2:668, 4:51 |
| No.38-Cs.cc1 | C46 | 0.062 | 1.0 | 2 | 0:246, 2:693, 4:61 |
| No.39-C2v.cc1 | C46 | 0.062 | 1.0 | 2 | 0:299, 2:648, 4:53 |
| No.4-C1.cc1 | C46 | 0.063 | 1.0 | 2 | 0:232, 2:683, 4:83, 6:2 |
| No.40-Cs.cc1 | C46 | 0.047 | 1.0 | 3 | 0:317, 2:627, 4:56 |
| No.41-Cs.cc1 | C46 | 0.047 | 1.0 | 2 | 0:297, 2:642, 4:60, 6:1 |
| No.42-C2v.cc1 | C46 | 0.062 | 1.0 | 3 | 0:300, 2:636, 4:64 |
| No.43-C2.cc1 | C46 | 0.063 | 1.0 | 2 | 0:272, 2:639, 4:89 |
| No.44-C1.cc1 | C46 | 0.047 | 1.0 | 2 | 0:295, 2:627, 4:78 |
| No.45-C1.cc1 | C46 | 0.141 | 1.0 | 2 | 0:248, 2:668, 4:84 |
| No.46-C1.cc1 | C46 | 0.156 | 1.0 | 2 | 0:291, 2:630, 4:79 |
| No.47-C2.cc1 | C46 | 0.156 | 1.0 | 2 | 0:224, 2:690, 4:86 |
| No.48-C1.cc1 | C46 | 0.125 | 1.0 | 2 | 0:242, 2:665, 4:92, 6:1 |
| No.49-C2.cc1 | C46 | 0.157 | 1.0 | 2 | 0:246, 2:659, 4:95 |
| No.5-C1.cc1 | C46 | 0.156 | 1.0 | 2 | 0:267, 2:654, 4:78, 6:1 |
| No.50-C1.cc1 | C46 | 0.141 | 1.0 | 2 | 0:286, 2:667, 4:47 |
| No.51-C1.cc1 | C46 | 0.109 | 1.0 | 2 | 0:258, 2:685, 4:57 |
| No.52-C1.cc1 | C46 | 0.094 | 1.0 | 2 | 0:283, 2:648, 4:68, 6:1 |
| No.53-C2.cc1 | C46 | 0.078 | 1.0 | 3 | 0:274, 2:659, 4:67 |
| No.54-C2.cc1 | C46 | 0.062 | 1.0 | 2 | 0:310, 2:616, 4:73, 6:1 |
| No.55-C1.cc1 | C46 | 0.062 | 1.0 | 2 | 0:313, 2:617, 4:69, 6:1 |
| No.56-C1.cc1 | C46 | 0.094 | 1.0 | 2 | 0:258, 2:687, 4:55 |
| No.57-Cs.cc1 | C46 | 0.140 | 1.0 | 3 | 0:245, 2:685, 4:69, 6:1 |
| No.58-C1.cc1 | C46 | 0.140 | 1.0 | 2 | 0:281, 2:659, 4:60 |
| No.59-C1.cc1 | C46 | 0.156 | 1.0 | 2 | 0:278, 2:652, 4:70 |
| No.6-C1.cc1 | C46 | 0.125 | 1.0 | 2 | 0:251, 2:665, 4:84 |
| No.60-C1.cc1 | C46 | 0.141 | 1.0 | 2 | 0:302, 2:644, 4:54 |
| No.61-C1.cc1 | C46 | 0.109 | 1.0 | 2 | 0:281, 2:668, 4:51 |
| No.62-C1.cc1 | C46 | 0.140 | 1.0 | 3 | 0:280, 2:651, 4:69 |
| No.63-C1.cc1 | C46 | 0.125 | 1.0 | 2 | 0:275, 2:672, 4:52, 6:1 |
| No.64-C1.cc1 | C46 | 0.094 | 1.0 | 2 | 0:279, 2:652, 4:68, 6:1 |
| No.65-Cs.cc1 | C46 | 0.078 | 1.0 | 2 | 0:280, 2:663, 4:57 |
| No.66-C2.cc1 | C46 | 0.078 | 1.0 | 2 | 0:278, 2:677, 4:45 |
| No.67-C1.cc1 | C46 | 0.094 | 1.0 | 2 | 0:314, 2:648, 4:38 |
| No.68-C1.cc1 | C46 | 0.140 | 1.0 | 3 | 0:274, 2:672, 4:54 |
| No.69-C1.cc1 | C46 | 0.141 | 1.0 | 2 | 0:295, 2:660, 4:45 |
| No.7-Cs.cc1 | C46 | 0.156 | 1.0 | 3 | 0:268, 2:667, 4:65 |
| No.70-C1.cc1 | C46 | 0.156 | 1.0 | 2 | 0:274, 2:664, 4:62 |
| No.71-C1.cc1 | C46 | 0.140 | 1.0 | 2 | 0:276, 2:658, 4:65, 6:1 |
| No.72-C1.cc1 | C46 | 0.187 | 1.0 | 2 | 0:287, 2:651, 4:62 |
| No.73-C1.cc1 | C46 | 0.109 | 1.0 | 2 | 0:297, 2:640, 4:63 |
| No.74-C1.cc1 | C46 | 0.110 | 1.0 | 2 | 0:281, 2:650, 4:69 |
| No.75-C1.cc1 | C46 | 0.078 | 1.0 | 2 | 0:291, 2:647, 4:62 |
| No.76-C1.cc1 | C46 | 0.079 | 1.0 | 2 | 0:279, 2:647, 4:73, 6:1 |
| No.77-C2.cc1 | C46 | 0.063 | 1.0 | 2 | 0:274, 2:643, 4:83 |
| No.78-C1.cc1 | C46 | 0.062 | 1.0 | 2 | 0:304, 2:645, 4:51 |
| No.79-C1.cc1 | C46 | 0.063 | 1.0 | 2 | 0:270, 2:674, 4:55, 6:1 |
| No.8-Cs.cc1 | C46 | 0.078 | 1.0 | 2 | 0:277, 2:639, 4:84 |
| No.80-C1.cc1 | C46 | 0.156 | 1.0 | 2 | 0:301, 2:630, 4:68, 6:1 |
| No.81-C1.cc1 | C46 | 0.109 | 1.0 | 2 | 0:256, 2:692, 4:52 |
| No.82-C1.cc1 | C46 | 0.109 | 1.0 | 2 | 0:273, 2:671, 4:56 |
| No.83-Cs.cc1 | C46 | 0.094 | 1.0 | 2 | 0:275, 2:672, 4:53 |
| No.84-C2.cc1 | C46 | 0.079 | 1.0 | 2 | 0:340, 2:621, 4:39 |
| No.85-C1.cc1 | C46 | 0.063 | 1.0 | 2 | 0:322, 2:621, 4:57 |
| No.86-C1.cc1 | C46 | 0.110 | 1.0 | 2 | 0:312, 2:640, 4:48 |
| No.87-C1.cc1 | C46 | 0.140 | 1.0 | 2 | 0:303, 2:644, 4:53 |
| No.88-C1.cc1 | C46 | 0.109 | 1.0 | 2 | 0:300, 2:657, 4:43 |
| No.89-Cs.cc1 | C46 | 0.078 | 1.0 | 3 | 0:303, 2:655, 4:42 |
| No.9-C2.cc1 | C46 | 0.063 | 1.0 | 2 | 0:275, 2:641, 4:83, 6:1 |
| No.90-C1.cc1 | C46 | 0.063 | 1.0 | 2 | 0:293, 2:667, 4:40 |
| No.91-C2v.cc1 | C46 | 0.062 | 1.0 | 2 | 0:367, 2:606, 4:27 |
| No.92-C2v.cc1 | C46 | 0.078 | 1.0 | 2 | 0:305, 2:648, 4:47 |
| No.93-C1.cc1 | C46 | 0.157 | 1.0 | 3 | 0:320, 2:647, 4:33 |
| No.94-C3.cc1 | C46 | 0.109 | 1.0 | 2 | 0:304, 2:652, 4:44 |
| No.95-C2.cc1 | C46 | 0.078 | 1.0 | 2 | 0:314, 2:655, 4:31 |
| No.96-C2.cc1 | C46 | 0.078 | 1.0 | 2 | 0:286, 2:663, 4:51 |
| No.97-C2.cc1 | C46 | 0.063 | 1.0 | 2 | 0:285, 2:655, 4:60 |
| No.98-C1.cc1 | C46 | 0.063 | 1.0 | 2 | 0:311, 2:634, 4:55 |
| No.99-Cs.cc1 | C46 | 0.047 | 1.0 | 2 | 0:295, 2:643, 4:62 |
| No.1-C2.cc1 | C48 | 0.062 | 1.0 | 3 | 0:193, 2:678, 4:128, 6:1 |
| No.10-C1.cc1 | C48 | 0.063 | 1.0 | 2 | 0:263, 2:630, 4:106, 6:1 |
| No.100-C1.cc1 | C48 | 0.125 | 1.0 | 2 | 0:243, 2:684, 4:73 |
| No.101-C1.cc1 | C48 | 0.141 | 1.0 | 2 | 0:241, 2:686, 4:73 |
| No.102-C1.cc1 | C48 | 0.125 | 1.0 | 2 | 0:262, 2:677, 4:60, 6:1 |
| No.103-C1.cc1 | C48 | 0.093 | 1.0 | 2 | 0:282, 2:663, 4:54, 6:1 |
| No.104-C1.cc1 | C48 | 0.078 | 1.0 | 2 | 0:286, 2:653, 4:61 |
| No.105-C1.cc1 | C48 | 0.078 | 1.0 | 2 | 0:233, 2:713, 4:54 |
| No.106-C1.cc1 | C48 | 0.078 | 1.0 | 2 | 0:242, 2:689, 4:69 |
| No.107-C2.cc1 | C48 | 0.063 | 1.0 | 3 | 0:275, 2:660, 4:64, 6:1 |
| No.108-C1.cc1 | C48 | 0.047 | 1.0 | 2 | 0:298, 2:631, 4:71 |
| No.109-C1.cc1 | C48 | 0.078 | 1.0 | 3 | 0:272, 2:667, 4:60, 6:1 |
| No.11-C1.cc1 | C48 | 0.125 | 1.0 | 2 | 0:250, 2:636, 4:112, 6:2 |
| No.110-C1.cc1 | C48 | 0.141 | 1.0 | 2 | 0:252, 2:683, 4:65 |
| No.111-C1.cc1 | C48 | 0.094 | 1.0 | 2 | 0:263, 2:680, 4:57 |
| No.112-C1.cc1 | C48 | 0.125 | 1.0 | 2 | 0:255, 2:683, 4:62 |
| No.113-C1.cc1 | C48 | 0.141 | 1.0 | 2 | 0:290, 2:647, 4:63 |
| No.114-C1.cc1 | C48 | 0.156 | 1.0 | 2 | 0:261, 2:677, 4:62 |
| No.115-C2.cc1 | C48 | 0.140 | 1.0 | 2 | 0:258, 2:672, 4:70 |
| No.116-C1.cc1 | C48 | 0.157 | 1.0 | 2 | 0:284, 2:651, 4:64, 6:1 |
| No.117-C1.cc1 | C48 | 0.156 | 1.0 | 2 | 0:243, 2:675, 4:82 |
| No.118-C1.cc1 | C48 | 0.156 | 1.0 | 2 | 0:262, 2:687, 4:51 |
| No.119-C1.cc1 | C48 | 0.109 | 1.0 | 2 | 0:219, 2:701, 4:80 |
| No.12-C1.cc1 | C48 | 0.110 | 1.0 | 2 | 0:226, 2:678, 4:93, 6:3 |
| No.120-C1.cc1 | C48 | 0.140 | 1.0 | 2 | 0:251, 2:670, 4:78, 6:1 |
| No.121-C1.cc1 | C48 | 0.094 | 1.0 | 2 | 0:263, 2:661, 4:76 |
| No.122-C2.cc1 | C48 | 0.078 | 1.0 | 2 | 0:263, 2:646, 4:90, 6:1 |
| No.123-C1.cc1 | C48 | 0.094 | 1.0 | 2 | 0:234, 2:682, 4:84 |
| No.124-C1.cc1 | C48 | 0.125 | 1.0 | 2 | 0:247, 2:695, 4:58 |
| No.125-C2.cc1 | C48 | 0.157 | 1.0 | 2 | 0:243, 2:685, 4:72 |
| No.126-C1.cc1 | C48 | 0.156 | 1.0 | 2 | 0:282, 2:669, 4:49 |
| No.127-C1.cc1 | C48 | 0.157 | 1.0 | 2 | 0:281, 2:673, 4:46 |
| No.128-C1.cc1 | C48 | 0.125 | 1.0 | 2 | 0:276, 2:668, 4:54, 6:2 |
| No.129-C1.cc1 | C48 | 0.156 | 1.0 | 2 | 0:245, 2:692, 4:63 |
| No.13-C1.cc1 | C48 | 0.109 | 1.0 | 3 | 0:244, 2:655, 4:99, 6:2 |
| No.130-C1.cc1 | C48 | 0.094 | 1.0 | 2 | 0:289, 2:645, 4:66 |
| No.131-C1.cc1 | C48 | 0.078 | 1.0 | 2 | 0:299, 2:659, 4:42 |
| No.132-C1.cc1 | C48 | 0.079 | 1.0 | 2 | 0:289, 2:646, 4:64, 6:1 |
| No.133-C1.cc1 | C48 | 0.062 | 1.0 | 2 | 0:307, 2:649, 4:44 |
| No.134-C2.cc1 | C48 | 0.047 | 1.0 | 3 | 0:269, 2:675, 4:56 |
| No.135-C1.cc1 | C48 | 0.156 | 1.0 | 2 | 0:292, 2:665, 4:43 |
| No.136-Cs.cc1 | C48 | 0.156 | 1.0 | 3 | 0:281, 2:656, 4:63 |
| No.137-C2.cc1 | C48 | 0.109 | 1.0 | 2 | 0:306, 2:651, 4:43 |
| No.138-C2v.cc1 | C48 | 0.078 | 1.0 | 2 | 0:286, 2:679, 4:35 |
| No.139-C1.cc1 | C48 | 0.156 | 1.0 | 2 | 0:278, 2:672, 4:50 |
| No.14-C2.cc1 | C48 | 0.156 | 1.0 | 2 | 0:248, 2:658, 4:93, 6:1 |
| No.140-C1.cc1 | C48 | 0.109 | 1.0 | 2 | 0:266, 2:671, 4:63 |
| No.141-Cs.cc1 | C48 | 0.094 | 1.0 | 2 | 0:261, 2:670, 4:68, 6:1 |
| No.142-Cs.cc1 | C48 | 0.078 | 1.0 | 2 | 0:303, 2:647, 4:50 |
| No.143-C1.cc1 | C48 | 0.078 | 1.0 | 2 | 0:327, 2:627, 4:46 |
| No.144-D2.cc1 | C48 | 0.063 | 1.0 | 3 | 0:304, 2:639, 4:55, 6:2 |
| No.145-C1.cc1 | C48 | 0.062 | 1.0 | 2 | 0:322, 2:635, 4:43 |
| No.146-C1.cc1 | C48 | 0.063 | 1.0 | 2 | 0:267, 2:675, 4:56, 6:2 |
| No.147-C1.cc1 | C48 | 0.047 | 1.0 | 2 | 0:285, 2:667, 4:48 |
| No.148-Cs.cc1 | C48 | 0.047 | 1.0 | 2 | 0:302, 2:656, 4:41, 6:1 |
| No.149-C1.cc1 | C48 | 0.047 | 1.0 | 2 | 0:300, 2:652, 4:47, 6:1 |
| No.15-D2h.cc1 | C48 | 0.047 | 1.0 | 2 | 0:214, 2:664, 4:121, 6:1 |
| No.150-C1.cc1 | C48 | 0.046 | 1.0 | 2 | 0:322, 2:642, 4:36 |
| No.151-C1.cc1 | C48 | 0.047 | 1.0 | 3 | 0:277, 2:679, 4:44 |
| No.152-C2.cc1 | C48 | 0.047 | 1.0 | 2 | 0:279, 2:665, 4:56 |
| No.153-C2.cc1 | C48 | 0.063 | 1.0 | 3 | 0:283, 2:657, 4:60 |
| No.154-C1.cc1 | C48 | 0.078 | 1.0 | 2 | 0:276, 2:656, 4:67, 6:1 |
| No.155-C1.cc1 | C48 | 0.157 | 1.0 | 2 | 0:292, 2:653, 4:53, 6:2 |
| No.156-C1.cc1 | C48 | 0.125 | 1.0 | 2 | 0:326, 2:628, 4:46 |
| No.157-C2.cc1 | C48 | 0.140 | 1.0 | 2 | 0:294, 2:624, 4:82 |
| No.158-C2.cc1 | C48 | 0.109 | 1.0 | 2 | 0:277, 2:658, 4:65 |
| No.159-C1.cc1 | C48 | 0.156 | 1.0 | 2 | 0:292, 2:646, 4:62 |
| No.16-D2.cc1 | C48 | 0.187 | 1.0 | 2 | 0:214, 2:662, 4:124 |
| No.160-C2.cc1 | C48 | 0.094 | 1.0 | 2 | 0:298, 2:638, 4:64 |
| No.161-C2.cc1 | C48 | 0.093 | 1.0 | 2 | 0:311, 2:628, 4:61 |
| No.162-C1.cc1 | C48 | 0.079 | 1.0 | 2 | 0:267, 2:679, 4:52, 6:2 |
| No.163-C2.cc1 | C48 | 0.062 | 1.0 | 2 | 0:274, 2:679, 4:47 |
| No.164-C1.cc1 | C48 | 0.063 | 1.0 | 2 | 0:272, 2:665, 4:62, 6:1 |
| No.165-C1.cc1 | C48 | 0.062 | 1.0 | 3 | 0:274, 2:682, 4:43, 6:1 |
| No.166-C2.cc1 | C48 | 0.047 | 1.0 | 2 | 0:272, 2:676, 4:52 |
| No.167-C1.cc1 | C48 | 0.078 | 1.0 | 2 | 0:274, 2:678, 4:48 |
| No.168-Cs.cc1 | C48 | 0.110 | 1.0 | 3 | 0:299, 2:643, 4:58 |
| No.169-D2.cc1 | C48 | 0.125 | 1.0 | 2 | 0:303, 2:647, 4:50 |
| No.17-C2v.cc1 | C48 | 0.093 | 1.0 | 2 | 0:276, 2:647, 4:76, 6:1 |
| No.170-C2.cc1 | C48 | 0.079 | 1.0 | 2 | 0:304, 2:658, 4:37, 6:1 |
| No.171-C2.cc1 | C48 | 0.063 | 1.0 | 3 | 0:304, 2:649, 4:47 |
| No.172-C1.cc1 | C48 | 0.063 | 1.0 | 2 | 0:267, 2:648, 4:85 |
| No.173-C1.cc1 | C48 | 0.062 | 1.0 | 2 | 0:271, 2:668, 4:60, 6:1 |
| No.174-C1.cc1 | C48 | 0.047 | 1.0 | 2 | 0:249, 2:672, 4:77, 6:2 |
| No.175-C2.cc1 | C48 | 0.047 | 1.0 | 2 | 0:260, 2:663, 4:77 |
| No.176-C1.cc1 | C48 | 0.093 | 1.0 | 2 | 0:304, 2:628, 4:68 |
| No.177-C2.cc1 | C48 | 0.110 | 1.0 | 2 | 0:285, 2:646, 4:69 |
| No.178-C1.cc1 | C48 | 0.141 | 1.0 | 2 | 0:303, 2:649, 4:48 |
| No.179-C1.cc1 | C48 | 0.156 | 1.0 | 3 | 0:256, 2:684, 4:60 |
| No.18-C1.cc1 | C48 | 0.156 | 1.0 | 2 | 0:237, 2:657, 4:105, 6:1 |
| No.180-C2.cc1 | C48 | 0.110 | 1.0 | 3 | 0:281, 2:649, 4:69, 6:1 |
| No.181-C1.cc1 | C48 | 0.110 | 1.0 | 2 | 0:263, 2:651, 4:85, 6:1 |
| No.182-C1.cc1 | C48 | 0.094 | 1.0 | 2 | 0:244, 2:691, 4:64, 6:1 |
| No.183-C2.cc1 | C48 | 0.078 | 1.0 | 3 | 0:267, 2:673, 4:59, 6:1 |
| No.184-Cs.cc1 | C48 | 0.125 | 1.0 | 2 | 0:285, 2:636, 4:78, 6:1 |
| No.185-C2.cc1 | C48 | 0.125 | 1.0 | 2 | 0:271, 2:678, 4:50, 6:1 |
| No.186-D6d.cc1 | C48 | 0.094 | 1.0 | 2 | 0:209, 2:664, 4:123, 6:4 |
| No.187-Cs.cc1 | C48 | 0.078 | 1.0 | 2 | 0:251, 2:696, 4:53 |
| No.188-D3.cc1 | C48 | 0.063 | 1.0 | 2 | 0:286, 2:657, 4:57 |
| No.189-D6d.cc1 | C48 | 0.063 | 1.0 | 2 | 0:286, 2:635, 4:79 |
| No.19-C1.cc1 | C48 | 0.062 | 1.0 | 2 | 0:249, 2:668, 4:83 |
| No.190-C2.cc1 | C48 | 0.125 | 1.0 | 2 | 0:310, 2:628, 4:62 |
| No.191-C2.cc1 | C48 | 0.140 | 1.0 | 2 | 0:290, 2:665, 4:45 |
| No.192-C2.cc1 | C48 | 0.109 | 1.0 | 2 | 0:304, 2:642, 4:54 |
| No.193-C1.cc1 | C48 | 0.094 | 1.0 | 3 | 0:309, 2:646, 4:45 |
| No.194-C2.cc1 | C48 | 0.078 | 1.0 | 2 | 0:310, 2:647, 4:42, 6:1 |
| No.195-C1.cc1 | C48 | 0.078 | 1.0 | 2 | 0:286, 2:662, 4:52 |
| No.196-C1.cc1 | C48 | 0.078 | 1.0 | 2 | 0:252, 2:667, 4:79, 6:2 |
| No.197-Cs.cc1 | C48 | 0.063 | 1.0 | 2 | 0:304, 2:657, 4:39 |
| No.198-D2.cc1 | C48 | 0.047 | 1.0 | 2 | 0:304, 2:646, 4:48, 6:2 |
| No.199-C2.cc1 | C48 | 0.110 | 1.0 | 2 | 0:317, 2:650, 4:33 |
| No.2-D2.cc1 | C48 | 0.125 | 1.0 | 3 | 0:193, 2:677, 4:126, 6:4 |
| No.20-C1.cc1 | C48 | 0.156 | 1.0 | 2 | 0:243, 2:657, 4:98, 6:2 |
| No.21-C1.cc1 | C48 | 0.157 | 1.0 | 2 | 0:278, 2:654, 4:68 |
| No.22-C1.cc1 | C48 | 0.110 | 1.0 | 2 | 0:283, 2:645, 4:70, 6:2 |
| No.23-C1.cc1 | C48 | 0.125 | 1.0 | 3 | 0:240, 2:660, 4:100 |
| No.24-C2.cc1 | C48 | 0.093 | 1.0 | 2 | 0:242, 2:689, 4:69 |
| No.25-C1.cc1 | C48 | 0.109 | 1.0 | 2 | 0:297, 2:622, 4:81 |
| No.26-C1.cc1 | C48 | 0.141 | 1.0 | 2 | 0:182, 2:717, 4:101 |
| No.27-C2.cc1 | C48 | 0.110 | 1.0 | 2 | 0:253, 2:652, 4:94, 6:1 |
| No.28-C1.cc1 | C48 | 0.078 | 1.0 | 2 | 0:279, 2:652, 4:68, 6:1 |
| No.29-C1.cc1 | C48 | 0.079 | 1.0 | 2 | 0:246, 2:676, 4:78 |
| No.3-C1.cc1 | C48 | 0.109 | 1.0 | 3 | 0:246, 2:663, 4:90, 6:1 |
| No.30-C1.cc1 | C48 | 0.156 | 1.0 | 2 | 0:239, 2:682, 4:79 |
| No.31-Cs.cc1 | C48 | 0.156 | 1.0 | 2 | 0:219, 2:685, 4:95, 6:1 |
| No.32-C2.cc1 | C48 | 0.156 | 1.0 | 2 | 0:334, 2:607, 4:59 |
| No.33-C1.cc1 | C48 | 0.172 | 1.0 | 2 | 0:303, 2:635, 4:61, 6:1 |
| No.34-C1.cc1 | C48 | 0.110 | 1.0 | 2 | 0:264, 2:666, 4:70 |
| No.35-C1.cc1 | C48 | 0.156 | 1.0 | 2 | 0:235, 2:661, 4:103, 6:1 |
| No.36-C1.cc1 | C48 | 0.110 | 1.0 | 2 | 0:282, 2:631, 4:86, 6:1 |
| No.37-C2.cc1 | C48 | 0.141 | 1.0 | 2 | 0:281, 2:670, 4:49 |
| No.38-C1.cc1 | C48 | 0.109 | 1.0 | 2 | 0:309, 2:636, 4:54, 6:1 |
| No.39-Cs.cc1 | C48 | 0.078 | 1.0 | 2 | 0:334, 2:610, 4:56 |
| No.4-Cs.cc1 | C48 | 0.141 | 1.0 | 2 | 0:250, 2:646, 4:104 |
| No.40-C2.cc1 | C48 | 0.125 | 1.0 | 2 | 0:310, 2:636, 4:54 |
| No.41-D2h.cc1 | C48 | 0.141 | 1.0 | 2 | 0:299, 2:641, 4:59, 6:1 |
| No.42-C1.cc1 | C48 | 0.156 | 1.0 | 2 | 0:303, 2:631, 4:66 |
| No.43-C2.cc1 | C48 | 0.109 | 1.0 | 2 | 0:290, 2:642, 4:68 |
| No.44-C1.cc1 | C48 | 0.156 | 1.0 | 2 | 0:294, 2:634, 4:72 |
| No.45-C2.cc1 | C48 | 0.109 | 1.0 | 2 | 0:316, 2:621, 4:63 |
| No.46-C2.cc1 | C48 | 0.078 | 1.0 | 2 | 0:204, 2:712, 4:83, 6:1 |
| No.47-C1.cc1 | C48 | 0.140 | 1.0 | 2 | 0:263, 2:678, 4:59 |
| No.48-C1.cc1 | C48 | 0.125 | 1.0 | 2 | 0:244, 2:689, 4:67 |
| No.49-C1.cc1 | C48 | 0.110 | 1.0 | 3 | 0:256, 2:684, 4:60 |
| No.5-C2.cc1 | C48 | 0.125 | 1.0 | 2 | 0:208, 2:690, 4:100, 6:2 |
| No.50-C1.cc1 | C48 | 0.094 | 1.0 | 2 | 0:286, 2:644, 4:70 |
| No.51-C1.cc1 | C48 | 0.078 | 1.0 | 2 | 0:295, 2:636, 4:69 |
| No.52-C1.cc1 | C48 | 0.078 | 1.0 | 2 | 0:278, 2:645, 4:77 |
| No.53-C1.cc1 | C48 | 0.078 | 1.0 | 2 | 0:267, 2:662, 4:71 |
| No.54-C1.cc1 | C48 | 0.078 | 1.0 | 2 | 0:268, 2:655, 4:76, 6:1 |
| No.55-C1.cc1 | C48 | 0.063 | 1.0 | 2 | 0:263, 2:665, 4:72 |
| No.56-C2v.cc1 | C48 | 0.062 | 1.0 | 2 | 0:259, 2:677, 4:63, 6:1 |
| No.57-C1.cc1 | C48 | 0.078 | 1.0 | 3 | 0:249, 2:663, 4:88 |
| No.58-C2.cc1 | C48 | 0.063 | 1.0 | 2 | 0:261, 2:672, 4:66, 6:1 |
| No.59-C2.cc1 | C48 | 0.046 | 1.0 | 2 | 0:224, 2:696, 4:79, 6:1 |
| No.6-C1.cc1 | C48 | 0.047 | 1.0 | 2 | 0:228, 2:680, 4:90, 6:2 |
| No.60-C1.cc1 | C48 | 0.078 | 1.0 | 2 | 0:264, 2:660, 4:75, 6:1 |
| No.61-C2.cc1 | C48 | 0.078 | 1.0 | 2 | 0:281, 2:656, 4:63 |
| No.62-Cs.cc1 | C48 | 0.063 | 1.0 | 2 | 0:282, 2:648, 4:68, 6:2 |
| No.63-C2.cc1 | C48 | 0.062 | 1.0 | 3 | 0:241, 2:680, 4:76, 6:3 |
| No.64-C2.cc1 | C48 | 0.063 | 1.0 | 2 | 0:220, 2:678, 4:102 |
| No.65-C1.cc1 | C48 | 0.156 | 1.0 | 2 | 0:245, 2:661, 4:94 |
| No.66-C1.cc1 | C48 | 0.109 | 1.0 | 2 | 0:259, 2:653, 4:88 |
| No.67-C1.cc1 | C48 | 0.094 | 1.0 | 3 | 0:305, 2:624, 4:71 |
| No.68-C2.cc1 | C48 | 0.109 | 1.0 | 2 | 0:214, 2:692, 4:93, 6:1 |
| No.69-C1.cc1 | C48 | 0.094 | 1.0 | 2 | 0:242, 2:644, 4:114 |
| No.7-C1.cc1 | C48 | 0.079 | 1.0 | 2 | 0:232, 2:664, 4:104 |
| No.70-C2.cc1 | C48 | 0.109 | 1.0 | 2 | 0:218, 2:683, 4:98, 6:1 |
| No.71-C1.cc1 | C48 | 0.140 | 1.0 | 2 | 0:296, 2:633, 4:71 |
| No.72-C1.cc1 | C48 | 0.141 | 1.0 | 2 | 0:265, 2:681, 4:54 |
| No.73-C1.cc1 | C48 | 0.141 | 1.0 | 2 | 0:264, 2:662, 4:74 |
| No.74-Cs.cc1 | C48 | 0.156 | 1.0 | 2 | 0:276, 2:645, 4:79 |
| No.75-Cs.cc1 | C48 | 0.109 | 1.0 | 2 | 0:231, 2:668, 4:101 |
| No.76-C2.cc1 | C48 | 0.094 | 1.0 | 2 | 0:275, 2:637, 4:88 |
| No.77-C1.cc1 | C48 | 0.078 | 1.0 | 2 | 0:321, 2:592, 4:87 |
| No.78-C2.cc1 | C48 | 0.063 | 1.0 | 2 | 0:293, 2:643, 4:64 |
| No.79-C1.cc1 | C48 | 0.063 | 1.0 | 2 | 0:248, 2:657, 4:95 |
| No.8-C1.cc1 | C48 | 0.047 | 1.0 | 3 | 0:267, 2:645, 4:88 |
| No.80-C2h.cc1 | C48 | 0.062 | 1.0 | 2 | 0:309, 2:621, 4:70 |
| No.81-C2.cc1 | C48 | 0.047 | 1.0 | 2 | 0:318, 2:623, 4:59 |
| No.82-C2.cc1 | C48 | 0.047 | 1.0 | 2 | 0:341, 2:602, 4:57 |
| No.83-C2.cc1 | C48 | 0.062 | 1.0 | 2 | 0:303, 2:638, 4:59 |
| No.84-C2.cc1 | C48 | 0.047 | 1.0 | 2 | 0:293, 2:641, 4:66 |
| No.85-C1.cc1 | C48 | 0.047 | 1.0 | 2 | 0:293, 2:660, 4:47 |
| No.86-C1.cc1 | C48 | 0.063 | 1.0 | 2 | 0:244, 2:674, 4:82 |
| No.87-C1.cc1 | C48 | 0.047 | 1.0 | 2 | 0:259, 2:684, 4:56, 6:1 |
| No.88-C1.cc1 | C48 | 0.046 | 1.0 | 2 | 0:264, 2:677, 4:59 |
| No.89-Cs.cc1 | C48 | 0.047 | 1.0 | 3 | 0:281, 2:652, 4:66, 6:1 |
| No.9-C1.cc1 | C48 | 0.047 | 1.0 | 3 | 0:232, 2:673, 4:95 |
| No.90-C1.cc1 | C48 | 0.047 | 1.0 | 2 | 0:276, 2:670, 4:54 |
| No.91-C1.cc1 | C48 | 0.063 | 1.0 | 2 | 0:312, 2:638, 4:49, 6:1 |
| No.92-C1.cc1 | C48 | 0.062 | 1.0 | 3 | 0:287, 2:653, 4:59, 6:1 |
| No.93-C1.cc1 | C48 | 0.063 | 1.0 | 2 | 0:285, 2:643, 4:71, 6:1 |
| No.94-C1.cc1 | C48 | 0.047 | 1.0 | 3 | 0:294, 2:640, 4:65, 6:1 |
| No.95-C2.cc1 | C48 | 0.047 | 1.0 | 2 | 0:290, 2:651, 4:59 |
| No.96-Cs.cc1 | C48 | 0.062 | 1.0 | 2 | 0:229, 2:685, 4:86 |
| No.97-C2.cc1 | C48 | 0.078 | 1.0 | 2 | 0:275, 2:657, 4:68 |
| No.98-C1.cc1 | C48 | 0.156 | 1.0 | 2 | 0:286, 2:637, 4:76, 6:1 |
| No.99-C1.cc1 | C48 | 0.157 | 1.0 | 2 | 0:279, 2:665, 4:56 |
| No.1-D5h.cc1 | C50 | 0.157 | 1.0 | 3 | 0:174, 2:668, 4:157, 6:1 |
| No.10-C1.cc1 | C50 | 0.125 | 1.0 | 3 | 0:214, 2:682, 4:101, 6:3 |
| No.100-Cs.cc1 | C50 | 0.094 | 1.0 | 2 | 0:282, 2:643, 4:75 |
| No.101-C1.cc1 | C50 | 0.109 | 1.0 | 2 | 0:247, 2:664, 4:89 |
| No.102-C1.cc1 | C50 | 0.141 | 1.0 | 2 | 0:263, 2:674, 4:63 |
| No.103-C1.cc1 | C50 | 0.093 | 1.0 | 2 | 0:240, 2:652, 4:107, 6:1 |
| No.104-C1.cc1 | C50 | 0.094 | 1.0 | 2 | 0:275, 2:650, 4:74, 6:1 |
| No.105-C1.cc1 | C50 | 0.078 | 1.0 | 2 | 0:247, 2:660, 4:92, 6:1 |
| No.106-C1.cc1 | C50 | 0.063 | 1.0 | 2 | 0:277, 2:664, 4:59 |
| No.107-C1.cc1 | C50 | 0.109 | 1.0 | 3 | 0:312, 2:615, 4:73 |
| No.108-C2.cc1 | C50 | 0.140 | 1.0 | 2 | 0:257, 2:664, 4:76, 6:3 |
| No.109-C1.cc1 | C50 | 0.125 | 1.0 | 2 | 0:255, 2:671, 4:74 |
| No.11-C1.cc1 | C50 | 0.094 | 1.0 | 2 | 0:236, 2:660, 4:103, 6:1 |
| No.110-C1.cc1 | C50 | 0.078 | 1.0 | 2 | 0:292, 2:655, 4:53 |
| No.111-C1.cc1 | C50 | 0.109 | 1.0 | 2 | 0:298, 2:639, 4:63 |
| No.112-C1.cc1 | C50 | 0.156 | 1.0 | 2 | 0:242, 2:660, 4:98 |
| No.113-C1.cc1 | C50 | 0.157 | 1.0 | 2 | 0:308, 2:619, 4:73 |
| No.114-C1.cc1 | C50 | 0.156 | 1.0 | 2 | 0:300, 2:616, 4:83, 6:1 |
| No.115-C1.cc1 | C50 | 0.109 | 1.0 | 2 | 0:293, 2:635, 4:71, 6:1 |
| No.116-C2.cc1 | C50 | 0.093 | 1.0 | 2 | 0:257, 2:643, 4:97, 6:3 |
| No.117-C1.cc1 | C50 | 0.078 | 1.0 | 2 | 0:288, 2:663, 4:48, 6:1 |
| No.118-C1.cc1 | C50 | 0.109 | 1.0 | 2 | 0:280, 2:628, 4:92 |
| No.119-C1.cc1 | C50 | 0.141 | 1.0 | 2 | 0:298, 2:628, 4:74 |
| No.12-C1.cc1 | C50 | 0.156 | 1.0 | 2 | 0:209, 2:699, 4:90, 6:2 |
| No.120-C1.cc1 | C50 | 0.172 | 1.0 | 2 | 0:241, 2:661, 4:96, 6:2 |
| No.121-C1.cc1 | C50 | 0.125 | 1.0 | 3 | 0:251, 2:678, 4:70, 6:1 |
| No.122-C1.cc1 | C50 | 0.140 | 1.0 | 3 | 0:256, 2:676, 4:68 |
| No.123-C1.cc1 | C50 | 0.156 | 1.0 | 2 | 0:253, 2:679, 4:68 |
| No.124-C1.cc1 | C50 | 0.141 | 1.0 | 2 | 0:258, 2:664, 4:78 |
| No.125-C2v.cc1 | C50 | 0.110 | 1.0 | 2 | 0:258, 2:620, 4:122 |
| No.126-C1.cc1 | C50 | 0.140 | 1.0 | 2 | 0:239, 2:669, 4:92 |
| No.127-C1.cc1 | C50 | 0.156 | 1.0 | 2 | 0:247, 2:658, 4:95 |
| No.128-C1.cc1 | C50 | 0.125 | 1.0 | 2 | 0:259, 2:647, 4:94 |
| No.129-C1.cc1 | C50 | 0.125 | 1.0 | 2 | 0:270, 2:636, 4:91, 6:3 |
| No.13-C2v.cc1 | C50 | 0.156 | 1.0 | 3 | 0:221, 2:647, 4:128, 6:4 |
| No.130-C1.cc1 | C50 | 0.156 | 1.0 | 2 | 0:260, 2:649, 4:88, 6:3 |
| No.131-C1.cc1 | C50 | 0.109 | 1.0 | 2 | 0:274, 2:638, 4:87, 6:1 |
| No.132-C1.cc1 | C50 | 0.094 | 1.0 | 2 | 0:276, 2:637, 4:87 |
| No.133-C1.cc1 | C50 | 0.125 | 1.0 | 2 | 0:237, 2:648, 4:115 |
| No.134-C1.cc1 | C50 | 0.109 | 1.0 | 2 | 0:252, 2:644, 4:104 |
| No.135-C1.cc1 | C50 | 0.156 | 1.0 | 2 | 0:240, 2:649, 4:111 |
| No.136-C1.cc1 | C50 | 0.125 | 1.0 | 3 | 0:236, 2:672, 4:92 |
| No.137-C1.cc1 | C50 | 0.094 | 1.0 | 2 | 0:237, 2:669, 4:94 |
| No.138-C2.cc1 | C50 | 0.078 | 1.0 | 2 | 0:195, 2:675, 4:129, 6:1 |
| No.139-C1.cc1 | C50 | 0.078 | 1.0 | 2 | 0:212, 2:700, 4:88 |
| No.14-C1.cc1 | C50 | 0.063 | 1.0 | 2 | 0:223, 2:657, 4:118, 6:2 |
| No.140-C1.cc1 | C50 | 0.062 | 1.0 | 2 | 0:213, 2:692, 4:93, 6:2 |
| No.141-C1.cc1 | C50 | 0.063 | 1.0 | 2 | 0:247, 2:653, 4:98, 6:2 |
| No.142-C1.cc1 | C50 | 0.062 | 1.0 | 2 | 0:244, 2:670, 4:85, 6:1 |
| No.143-C1.cc1 | C50 | 0.172 | 1.0 | 2 | 0:288, 2:656, 4:55, 6:1 |
| No.144-C1.cc1 | C50 | 0.172 | 1.0 | 4 | 0:266, 2:673, 4:61 |
| No.145-C1.cc1 | C50 | 0.109 | 1.0 | 3 | 0:265, 2:678, 4:57 |
| No.146-C1.cc1 | C50 | 0.125 | 1.0 | 2 | 0:273, 2:661, 4:66 |
| No.147-C1.cc1 | C50 | 0.157 | 1.0 | 2 | 0:260, 2:667, 4:72, 6:1 |
| No.148-C1.cc1 | C50 | 0.125 | 1.0 | 2 | 0:208, 2:702, 4:88, 6:2 |
| No.149-C1.cc1 | C50 | 0.172 | 1.0 | 2 | 0:239, 2:700, 4:59, 6:2 |
| No.15-C1.cc1 | C50 | 0.156 | 1.0 | 3 | 0:238, 2:658, 4:103, 6:1 |
| No.150-C1.cc1 | C50 | 0.109 | 1.0 | 2 | 0:286, 2:663, 4:51 |
| No.151-C1.cc1 | C50 | 0.094 | 1.0 | 2 | 0:297, 2:641, 4:61, 6:1 |
| No.152-C1.cc1 | C50 | 0.156 | 1.0 | 3 | 0:233, 2:701, 4:66 |
| No.153-C1.cc1 | C50 | 0.156 | 1.0 | 2 | 0:263, 2:664, 4:73 |
| No.154-C1.cc1 | C50 | 0.156 | 1.0 | 2 | 0:247, 2:683, 4:69, 6:1 |
| No.155-C1.cc1 | C50 | 0.172 | 1.0 | 2 | 0:213, 2:678, 4:108, 6:1 |
| No.156-C2.cc1 | C50 | 0.156 | 1.0 | 2 | 0:240, 2:690, 4:69, 6:1 |
| No.157-C3v.cc1 | C50 | 0.109 | 1.0 | 2 | 0:289, 2:649, 4:61, 6:1 |
| No.158-Cs.cc1 | C50 | 0.109 | 1.0 | 2 | 0:271, 2:655, 4:72, 6:2 |
| No.159-C1.cc1 | C50 | 0.156 | 1.0 | 3 | 0:235, 2:701, 4:63, 6:1 |
| No.16-C1.cc1 | C50 | 0.110 | 1.0 | 2 | 0:235, 2:647, 4:118 |
| No.160-C1.cc1 | C50 | 0.110 | 1.0 | 3 | 0:295, 2:646, 4:59 |
| No.161-C1.cc1 | C50 | 0.110 | 1.0 | 2 | 0:251, 2:698, 4:51 |
| No.162-Cs.cc1 | C50 | 0.157 | 1.0 | 2 | 0:236, 2:691, 4:73 |
| No.163-C1.cc1 | C50 | 0.172 | 1.0 | 2 | 0:276, 2:641, 4:83 |
| No.164-C1.cc1 | C50 | 0.109 | 1.0 | 2 | 0:253, 2:665, 4:82 |
| No.165-C1.cc1 | C50 | 0.093 | 1.0 | 3 | 0:273, 2:658, 4:69 |
| No.166-C1.cc1 | C50 | 0.078 | 1.0 | 2 | 0:293, 2:649, 4:58 |
| No.167-C2.cc1 | C50 | 0.109 | 1.0 | 2 | 0:291, 2:628, 4:81 |
| No.168-Cs.cc1 | C50 | 0.125 | 1.0 | 3 | 0:313, 2:616, 4:71 |
| No.169-C1.cc1 | C50 | 0.109 | 1.0 | 3 | 0:292, 2:654, 4:53, 6:1 |
| No.17-C1.cc1 | C50 | 0.079 | 1.0 | 2 | 0:215, 2:669, 4:115, 6:1 |
| No.170-C1.cc1 | C50 | 0.078 | 1.0 | 3 | 0:257, 2:653, 4:89, 6:1 |
| No.171-C1.cc1 | C50 | 0.125 | 1.0 | 2 | 0:280, 2:671, 4:48, 6:1 |
| No.172-C1.cc1 | C50 | 0.157 | 1.0 | 2 | 0:317, 2:614, 4:69 |
| No.173-C1.cc1 | C50 | 0.140 | 1.0 | 2 | 0:315, 2:637, 4:48 |
| No.174-C1.cc1 | C50 | 0.156 | 1.0 | 2 | 0:286, 2:651, 4:63 |
| No.175-C2.cc1 | C50 | 0.156 | 1.0 | 2 | 0:247, 2:682, 4:71 |
| No.176-C1.cc1 | C50 | 0.110 | 1.0 | 3 | 0:283, 2:655, 4:62 |
| No.177-C1.cc1 | C50 | 0.078 | 1.0 | 2 | 0:303, 2:625, 4:71, 6:1 |
| No.178-C1.cc1 | C50 | 0.125 | 1.0 | 2 | 0:312, 2:621, 4:66, 6:1 |
| No.179-C2v.cc1 | C50 | 0.172 | 1.0 | 2 | 0:282, 2:682, 4:36 |
| No.18-C2.cc1 | C50 | 0.156 | 1.0 | 2 | 0:235, 2:654, 4:109, 6:2 |
| No.180-C2.cc1 | C50 | 0.109 | 1.0 | 2 | 0:265, 2:674, 4:61 |
| No.181-D3.cc1 | C50 | 0.140 | 1.0 | 2 | 0:295, 2:652, 4:53 |
| No.182-C1.cc1 | C50 | 0.141 | 1.0 | 2 | 0:286, 2:666, 4:46, 6:2 |
| No.183-C1.cc1 | C50 | 0.156 | 1.0 | 2 | 0:277, 2:666, 4:57 |
| No.184-C1.cc1 | C50 | 0.109 | 1.0 | 2 | 0:299, 2:641, 4:58, 6:2 |
| No.185-Cs.cc1 | C50 | 0.125 | 1.0 | 2 | 0:275, 2:656, 4:69 |
| No.186-C1.cc1 | C50 | 0.093 | 1.0 | 2 | 0:281, 2:668, 4:50, 6:1 |
| No.187-C1.cc1 | C50 | 0.141 | 1.0 | 2 | 0:269, 2:659, 4:72 |
| No.188-C1.cc1 | C50 | 0.110 | 1.0 | 2 | 0:314, 2:640, 4:46 |
| No.189-C1.cc1 | C50 | 0.110 | 1.0 | 2 | 0:294, 2:641, 4:64, 6:1 |
| No.19-C1.cc1 | C50 | 0.110 | 1.0 | 3 | 0:229, 2:652, 4:118, 6:1 |
| No.190-C1.cc1 | C50 | 0.078 | 1.0 | 2 | 0:252, 2:675, 4:73 |
| No.191-C1.cc1 | C50 | 0.078 | 1.0 | 2 | 0:258, 2:660, 4:81, 6:1 |
| No.192-C1.cc1 | C50 | 0.078 | 1.0 | 2 | 0:239, 2:675, 4:85, 6:1 |
| No.193-C1.cc1 | C50 | 0.062 | 1.0 | 3 | 0:289, 2:648, 4:63 |
| No.194-C1.cc1 | C50 | 0.047 | 1.0 | 2 | 0:264, 2:670, 4:65, 6:1 |
| No.195-C1.cc1 | C50 | 0.094 | 1.0 | 2 | 0:300, 2:639, 4:61 |
| No.196-C1.cc1 | C50 | 0.156 | 1.0 | 2 | 0:280, 2:647, 4:72, 6:1 |
| No.197-C1.cc1 | C50 | 0.094 | 1.0 | 2 | 0:281, 2:655, 4:64 |
| No.198-C1.cc1 | C50 | 0.078 | 1.0 | 2 | 0:249, 2:692, 4:58, 6:1 |
| No.199-C1.cc1 | C50 | 0.140 | 1.0 | 2 | 0:273, 2:673, 4:54 |
| No.2-C2.cc1 | C50 | 0.172 | 1.0 | 2 | 0:191, 2:672, 4:135, 6:2 |
| No.20-C1.cc1 | C50 | 0.110 | 1.0 | 3 | 0:303, 2:605, 4:89, 6:3 |
| No.200-C1.cc1 | C50 | 0.078 | 1.0 | 2 | 0:271, 2:683, 4:45, 6:1 |
| No.201-C1.cc1 | C50 | 0.063 | 1.0 | 2 | 0:258, 2:679, 4:63 |
| No.202-C1.cc1 | C50 | 0.063 | 1.0 | 2 | 0:273, 2:665, 4:62 |
| No.203-C2.cc1 | C50 | 0.078 | 1.0 | 3 | 0:271, 2:669, 4:60 |
| No.204-C1.cc1 | C50 | 0.094 | 1.0 | 2 | 0:271, 2:669, 4:60 |
| No.205-C2.cc1 | C50 | 0.078 | 1.0 | 2 | 0:266, 2:679, 4:55 |
| No.206-Cs.cc1 | C50 | 0.094 | 1.0 | 3 | 0:281, 2:657, 4:62 |
| No.207-Cs.cc1 | C50 | 0.078 | 1.0 | 2 | 0:307, 2:645, 4:48 |
| No.208-C3.cc1 | C50 | 0.079 | 1.0 | 2 | 0:304, 2:638, 4:57, 6:1 |
| No.209-C1.cc1 | C50 | 0.062 | 1.0 | 2 | 0:266, 2:664, 4:70 |
| No.21-C1.cc1 | C50 | 0.125 | 1.0 | 2 | 0:260, 2:642, 4:98 |
| No.210-C2.cc1 | C50 | 0.172 | 1.0 | 2 | 0:262, 2:657, 4:81 |
| No.211-C1.cc1 | C50 | 0.156 | 1.0 | 2 | 0:240, 2:685, 4:75 |
| No.212-C1.cc1 | C50 | 0.156 | 1.0 | 2 | 0:253, 2:678, 4:69 |
| No.213-C1.cc1 | C50 | 0.156 | 1.0 | 2 | 0:253, 2:677, 4:70 |
| No.214-C2.cc1 | C50 | 0.109 | 1.0 | 3 | 0:347, 2:598, 4:55 |
| No.215-C1.cc1 | C50 | 0.094 | 1.0 | 2 | 0:251, 2:682, 4:67 |
| No.216-C1.cc1 | C50 | 0.125 | 1.0 | 2 | 0:267, 2:657, 4:75, 6:1 |
| No.217-C1.cc1 | C50 | 0.156 | 1.0 | 2 | 0:316, 2:627, 4:57 |
| No.218-C1.cc1 | C50 | 0.156 | 1.0 | 2 | 0:266, 2:667, 4:67 |
| No.219-C1.cc1 | C50 | 0.109 | 1.0 | 2 | 0:285, 2:654, 4:60, 6:1 |
| No.22-C1.cc1 | C50 | 0.125 | 1.0 | 3 | 0:250, 2:660, 4:90 |
| No.220-C1.cc1 | C50 | 0.157 | 1.0 | 2 | 0:256, 2:678, 4:65, 6:1 |
| No.221-C1.cc1 | C50 | 0.157 | 1.0 | 2 | 0:275, 2:672, 4:53 |
| No.222-C1.cc1 | C50 | 0.156 | 1.0 | 2 | 0:262, 2:671, 4:67 |
| No.223-C1.cc1 | C50 | 0.110 | 1.0 | 2 | 0:304, 2:621, 4:74, 6:1 |
| No.224-C1.cc1 | C50 | 0.157 | 1.0 | 2 | 0:260, 2:664, 4:75, 6:1 |
| No.225-C1.cc1 | C50 | 0.125 | 1.0 | 2 | 0:271, 2:664, 4:64, 6:1 |
| No.226-C2.cc1 | C50 | 0.093 | 1.0 | 2 | 0:312, 2:637, 4:51 |
| No.227-C1.cc1 | C50 | 0.125 | 1.0 | 2 | 0:287, 2:627, 4:84, 6:2 |
| No.228-C1.cc1 | C50 | 0.125 | 1.0 | 2 | 0:247, 2:679, 4:73, 6:1 |
| No.229-C2.cc1 | C50 | 0.172 | 1.0 | 2 | 0:280, 2:632, 4:86, 6:2 |
| No.23-C1.cc1 | C50 | 0.156 | 1.0 | 2 | 0:239, 2:667, 4:93, 6:1 |
| No.230-C1.cc1 | C50 | 0.110 | 1.0 | 2 | 0:251, 2:654, 4:93, 6:2 |
| No.231-C1.cc1 | C50 | 0.110 | 1.0 | 2 | 0:260, 2:661, 4:78, 6:1 |
| No.232-C1.cc1 | C50 | 0.157 | 1.0 | 2 | 0:246, 2:671, 4:83 |
| No.233-C1.cc1 | C50 | 0.172 | 1.0 | 2 | 0:260, 2:674, 4:66 |
| No.234-C2.cc1 | C50 | 0.156 | 1.0 | 2 | 0:255, 2:655, 4:90 |
| No.235-C1.cc1 | C50 | 0.157 | 1.0 | 2 | 0:219, 2:683, 4:98 |
| No.236-C1.cc1 | C50 | 0.125 | 1.0 | 2 | 0:259, 2:657, 4:83, 6:1 |
| No.237-C1.cc1 | C50 | 0.125 | 1.0 | 2 | 0:253, 2:695, 4:52 |
| No.238-C2.cc1 | C50 | 0.110 | 1.0 | 2 | 0:238, 2:676, 4:85, 6:1 |
| No.239-C1.cc1 | C50 | 0.109 | 1.0 | 2 | 0:274, 2:659, 4:67 |
| No.24-C2.cc1 | C50 | 0.156 | 1.0 | 2 | 0:245, 2:649, 4:105, 6:1 |
| No.240-C1.cc1 | C50 | 0.188 | 1.0 | 2 | 0:290, 2:662, 4:48 |
| No.241-C1.cc1 | C50 | 0.125 | 1.0 | 2 | 0:294, 2:662, 4:44 |
| No.242-C2v.cc1 | C50 | 0.094 | 1.0 | 2 | 0:251, 2:666, 4:82, 6:1 |
| No.243-C1.cc1 | C50 | 0.094 | 1.0 | 2 | 0:229, 2:692, 4:78, 6:1 |
| No.244-C1.cc1 | C50 | 0.078 | 1.0 | 2 | 0:284, 2:663, 4:53 |
| No.245-C1.cc1 | C50 | 0.078 | 1.0 | 4 | 0:249, 2:682, 4:68, 6:1 |
| No.246-C1.cc1 | C50 | 0.063 | 1.0 | 2 | 0:284, 2:659, 4:57 |
| No.247-Cs.cc1 | C50 | 0.062 | 1.0 | 2 | 0:273, 2:655, 4:72 |
| No.248-C1.cc1 | C50 | 0.047 | 1.0 | 3 | 0:271, 2:676, 4:53 |
| No.249-C1.cc1 | C50 | 0.062 | 1.0 | 2 | 0:288, 2:653, 4:59 |
| No.25-C1.cc1 | C50 | 0.047 | 1.0 | 2 | 0:231, 2:642, 4:125, 6:2 |
| No.250-C2.cc1 | C50 | 0.063 | 1.0 | 2 | 0:270, 2:680, 4:50 |
| No.251-C1.cc1 | C50 | 0.156 | 1.0 | 2 | 0:289, 2:657, 4:54 |
| No.252-C1.cc1 | C50 | 0.109 | 1.0 | 3 | 0:286, 2:654, 4:59, 6:1 |
| No.253-Cs.cc1 | C50 | 0.172 | 1.0 | 3 | 0:298, 2:656, 4:45, 6:1 |
| No.254-C2.cc1 | C50 | 0.125 | 1.0 | 2 | 0:311, 2:640, 4:49 |
| No.255-C2.cc1 | C50 | 0.125 | 1.0 | 2 | 0:275, 2:659, 4:65, 6:1 |
| No.256-C2v.cc1 | C50 | 0.140 | 1.0 | 2 | 0:277, 2:655, 4:67, 6:1 |
| No.257-C2.cc1 | C50 | 0.109 | 1.0 | 2 | 0:280, 2:654, 4:66 |
| No.258-C3.cc1 | C50 | 0.078 | 1.0 | 2 | 0:264, 2:679, 4:56, 6:1 |
| No.259-C1.cc1 | C50 | 0.109 | 1.0 | 2 | 0:288, 2:652, 4:60 |
| No.26-C1.cc1 | C50 | 0.125 | 1.0 | 2 | 0:254, 2:650, 4:96 |
| No.260-C2.cc1 | C50 | 0.156 | 1.0 | 2 | 0:318, 2:629, 4:52, 6:1 |
| No.261-C2.cc1 | C50 | 0.156 | 1.0 | 3 | 0:307, 2:643, 4:50 |
| No.262-Cs.cc1 | C50 | 0.156 | 1.0 | 2 | 0:313, 2:633, 4:54 |
| No.263-C2.cc1 | C50 | 0.125 | 1.0 | 2 | 0:320, 2:634, 4:45, 6:1 |
| No.264-Cs.cc1 | C50 | 0.141 | 1.0 | 2 | 0:295, 2:657, 4:48 |
| No.265-C2.cc1 | C50 | 0.109 | 1.0 | 2 | 0:271, 2:682, 4:47 |
| No.266-Cs.cc1 | C50 | 0.078 | 1.0 | 2 | 0:308, 2:639, 4:53 |
| No.267-Cs.cc1 | C50 | 0.078 | 1.0 | 2 | 0:294, 2:657, 4:49 |
| No.268-C1.cc1 | C50 | 0.172 | 1.0 | 2 | 0:335, 2:602, 4:62, 6:1 |
| No.269-C2.cc1 | C50 | 0.156 | 1.0 | 2 | 0:264, 2:677, 4:58, 6:1 |
| No.27-C2.cc1 | C50 | 0.125 | 1.0 | 2 | 0:257, 2:661, 4:82 |
| No.270-D3.cc1 | C50 | 0.140 | 1.0 | 2 | 0:312, 2:656, 4:32 |
| No.271-D5h.cc1 | C50 | 0.125 | 1.0 | 2 | 0:316, 2:647, 4:37 |
| No.28-C1.cc1 | C50 | 0.093 | 1.0 | 2 | 0:242, 2:658, 4:100 |
| No.29-C1.cc1 | C50 | 0.110 | 1.0 | 2 | 0:241, 2:675, 4:84 |
| No.3-D3h.cc1 | C50 | 0.125 | 1.0 | 2 | 0:220, 2:636, 4:142, 6:2 |
| No.30-C1.cc1 | C50 | 0.125 | 1.0 | 2 | 0:258, 2:662, 4:80 |
| No.31-C1.cc1 | C50 | 0.156 | 1.0 | 2 | 0:277, 2:634, 4:89 |
| No.32-Cs.cc1 | C50 | 0.156 | 1.0 | 2 | 0:227, 2:675, 4:97, 6:1 |
| No.33-Cs.cc1 | C50 | 0.109 | 1.0 | 2 | 0:251, 2:647, 4:100, 6:2 |
| No.34-C1.cc1 | C50 | 0.156 | 1.0 | 2 | 0:274, 2:641, 4:85 |
| No.35-C1.cc1 | C50 | 0.109 | 1.0 | 3 | 0:256, 2:667, 4:76, 6:1 |
| No.36-C1.cc1 | C50 | 0.094 | 1.0 | 2 | 0:252, 2:664, 4:84 |
| No.37-C1.cc1 | C50 | 0.094 | 1.0 | 2 | 0:293, 2:627, 4:80 |
| No.38-C1.cc1 | C50 | 0.140 | 1.0 | 2 | 0:260, 2:650, 4:88, 6:2 |
| No.39-C1.cc1 | C50 | 0.141 | 1.0 | 2 | 0:282, 2:642, 4:74, 6:2 |
| No.4-Cs.cc1 | C50 | 0.140 | 1.0 | 3 | 0:215, 2:646, 4:138, 6:1 |
| No.40-C1.cc1 | C50 | 0.156 | 1.0 | 2 | 0:264, 2:653, 4:82, 6:1 |
| No.41-C1.cc1 | C50 | 0.125 | 1.0 | 3 | 0:249, 2:663, 4:86, 6:2 |
| No.42-Cs.cc1 | C50 | 0.125 | 1.0 | 2 | 0:268, 2:635, 4:97 |
| No.43-C2v.cc1 | C50 | 0.109 | 1.0 | 2 | 0:274, 2:643, 4:82, 6:1 |
| No.44-C1.cc1 | C50 | 0.156 | 1.0 | 3 | 0:270, 2:668, 4:62 |
| No.45-C1.cc1 | C50 | 0.125 | 1.0 | 2 | 0:241, 2:671, 4:87, 6:1 |
| No.46-C1.cc1 | C50 | 0.125 | 1.0 | 2 | 0:241, 2:671, 4:88 |
| No.47-C1.cc1 | C50 | 0.141 | 1.0 | 2 | 0:273, 2:647, 4:78, 6:2 |
| No.48-C1.cc1 | C50 | 0.172 | 1.0 | 3 | 0:229, 2:674, 4:97 |
| No.49-C1.cc1 | C50 | 0.109 | 1.0 | 2 | 0:272, 2:655, 4:73 |
| No.5-Cs.cc1 | C50 | 0.078 | 1.0 | 3 | 0:199, 2:672, 4:127, 6:2 |
| No.50-C1.cc1 | C50 | 0.110 | 1.0 | 2 | 0:238, 2:674, 4:88 |
| No.51-C1.cc1 | C50 | 0.078 | 1.0 | 2 | 0:293, 2:655, 4:52 |
| No.52-C1.cc1 | C50 | 0.125 | 1.0 | 2 | 0:296, 2:637, 4:67 |
| No.53-C1.cc1 | C50 | 0.141 | 1.0 | 2 | 0:264, 2:654, 4:81, 6:1 |
| No.54-C1.cc1 | C50 | 0.093 | 1.0 | 3 | 0:249, 2:675, 4:76 |
| No.55-C1.cc1 | C50 | 0.079 | 1.0 | 2 | 0:307, 2:631, 4:61, 6:1 |
| No.56-C1.cc1 | C50 | 0.063 | 1.0 | 2 | 0:231, 2:662, 4:105, 6:2 |
| No.57-C1.cc1 | C50 | 0.063 | 1.0 | 2 | 0:308, 2:617, 4:75 |
| No.58-C1.cc1 | C50 | 0.094 | 1.0 | 2 | 0:279, 2:657, 4:63, 6:1 |
| No.59-C1.cc1 | C50 | 0.140 | 1.0 | 3 | 0:172, 2:702, 4:125, 6:1 |
| No.6-C2.cc1 | C50 | 0.172 | 1.0 | 3 | 0:227, 2:638, 4:131, 6:4 |
| No.60-C1.cc1 | C50 | 0.156 | 1.0 | 2 | 0:224, 2:675, 4:100, 6:1 |
| No.61-C2.cc1 | C50 | 0.203 | 1.0 | 2 | 0:258, 2:668, 4:73, 6:1 |
| No.62-C1.cc1 | C50 | 0.141 | 1.0 | 2 | 0:248, 2:675, 4:76, 6:1 |
| No.63-C1.cc1 | C50 | 0.125 | 1.0 | 3 | 0:245, 2:656, 4:98, 6:1 |
| No.64-C2.cc1 | C50 | 0.094 | 1.0 | 3 | 0:219, 2:685, 4:95, 6:1 |
| No.65-C1.cc1 | C50 | 0.078 | 1.0 | 2 | 0:277, 2:635, 4:88 |
| No.66-C1.cc1 | C50 | 0.063 | 1.0 | 2 | 0:252, 2:667, 4:81 |
| No.67-C2.cc1 | C50 | 0.062 | 1.0 | 2 | 0:300, 2:603, 4:94, 6:3 |
| No.68-C1.cc1 | C50 | 0.063 | 1.0 | 3 | 0:265, 2:659, 4:74, 6:2 |
| No.69-Cs.cc1 | C50 | 0.093 | 1.0 | 3 | 0:294, 2:634, 4:71, 6:1 |
| No.7-C1.cc1 | C50 | 0.157 | 1.0 | 2 | 0:232, 2:651, 4:115, 6:2 |
| No.70-C1.cc1 | C50 | 0.110 | 1.0 | 3 | 0:283, 2:642, 4:75 |
| No.71-C1.cc1 | C50 | 0.093 | 1.0 | 2 | 0:261, 2:654, 4:85 |
| No.72-Cs.cc1 | C50 | 0.078 | 1.0 | 2 | 0:304, 2:626, 4:69, 6:1 |
| No.73-C1.cc1 | C50 | 0.079 | 1.0 | 2 | 0:246, 2:658, 4:96 |
| No.74-C2.cc1 | C50 | 0.062 | 1.0 | 2 | 0:242, 2:663, 4:94, 6:1 |
| No.75-C1.cc1 | C50 | 0.063 | 1.0 | 2 | 0:250, 2:649, 4:101 |
| No.76-C1.cc1 | C50 | 0.062 | 1.0 | 2 | 0:266, 2:631, 4:102, 6:1 |
| No.77-C1.cc1 | C50 | 0.047 | 1.0 | 2 | 0:277, 2:639, 4:83, 6:1 |
| No.78-C2.cc1 | C50 | 0.062 | 1.0 | 3 | 0:229, 2:639, 4:129, 6:3 |
| No.79-C1.cc1 | C50 | 0.047 | 1.0 | 2 | 0:217, 2:658, 4:123, 6:2 |
| No.8-Cs.cc1 | C50 | 0.078 | 1.0 | 3 | 0:215, 2:696, 4:88, 6:1 |
| No.80-C2.cc1 | C50 | 0.078 | 1.0 | 2 | 0:211, 2:658, 4:130, 6:1 |
| No.81-C1.cc1 | C50 | 0.078 | 1.0 | 2 | 0:263, 2:649, 4:87, 6:1 |
| No.82-C1.cc1 | C50 | 0.062 | 1.0 | 2 | 0:238, 2:675, 4:87 |
| No.83-C1.cc1 | C50 | 0.063 | 1.0 | 2 | 0:261, 2:654, 4:85 |
| No.84-C1.cc1 | C50 | 0.062 | 1.0 | 2 | 0:246, 2:665, 4:89 |
| No.85-C1.cc1 | C50 | 0.157 | 1.0 | 2 | 0:244, 2:663, 4:93 |
| No.86-Cs.cc1 | C50 | 0.125 | 1.0 | 3 | 0:256, 2:661, 4:82, 6:1 |
| No.87-C1.cc1 | C50 | 0.156 | 1.0 | 2 | 0:255, 2:660, 4:83, 6:2 |
| No.88-Cs.cc1 | C50 | 0.109 | 1.0 | 2 | 0:282, 2:627, 4:90, 6:1 |
| No.89-C1.cc1 | C50 | 0.125 | 1.0 | 2 | 0:221, 2:674, 4:105 |
| No.9-C1.cc1 | C50 | 0.156 | 1.0 | 3 | 0:221, 2:679, 4:98, 6:2 |
| No.90-C1.cc1 | C50 | 0.157 | 1.0 | 2 | 0:243, 2:666, 4:89, 6:2 |
| No.91-Cs.cc1 | C50 | 0.110 | 1.0 | 2 | 0:263, 2:662, 4:74, 6:1 |
| No.92-Cs.cc1 | C50 | 0.093 | 1.0 | 3 | 0:253, 2:667, 4:79, 6:1 |
| No.93-C2.cc1 | C50 | 0.141 | 1.0 | 2 | 0:246, 2:648, 4:104, 6:2 |
| No.94-C1.cc1 | C50 | 0.141 | 1.0 | 2 | 0:229, 2:671, 4:99, 6:1 |
| No.95-C2.cc1 | C50 | 0.156 | 1.0 | 2 | 0:236, 2:662, 4:102 |
| No.96-C1.cc1 | C50 | 0.109 | 1.0 | 2 | 0:261, 2:648, 4:90, 6:1 |
| No.97-C1.cc1 | C50 | 0.078 | 1.0 | 2 | 0:281, 2:648, 4:71 |
| No.98-C1.cc1 | C50 | 0.094 | 1.0 | 2 | 0:295, 2:636, 4:68, 6:1 |
| No.99-C1.cc1 | C50 | 0.109 | 1.0 | 2 | 0:280, 2:640, 4:79, 6:1 |
| No.1-C2.cc1 | C52 | 0.156 | 1.0 | 3 | 0:169, 2:663, 4:163, 6:5 |
| No.10-C1.cc1 | C52 | 0.172 | 1.0 | 2 | 0:230, 2:651, 4:118, 6:1 |
| No.100-C1.cc1 | C52 | 0.125 | 1.0 | 2 | 0:247, 2:676, 4:76, 6:1 |
| No.101-C2v.cc1 | C52 | 0.140 | 1.0 | 2 | 0:244, 2:661, 4:95 |
| No.102-Cs.cc1 | C52 | 0.156 | 1.0 | 3 | 0:229, 2:660, 4:109, 6:2 |
| No.103-D2h.cc1 | C52 | 0.125 | 1.0 | 2 | 0:224, 2:674, 4:102 |
| No.104-C1.cc1 | C52 | 0.094 | 1.0 | 2 | 0:256, 2:653, 4:90, 6:1 |
| No.105-C2.cc1 | C52 | 0.078 | 1.0 | 3 | 0:240, 2:655, 4:104, 6:1 |
| No.106-C2.cc1 | C52 | 0.063 | 1.0 | 2 | 0:256, 2:637, 4:105, 6:2 |
| No.107-C2.cc1 | C52 | 0.078 | 1.0 | 2 | 0:279, 2:643, 4:77, 6:1 |
| No.108-C2.cc1 | C52 | 0.078 | 1.0 | 2 | 0:218, 2:693, 4:88, 6:1 |
| No.109-C3v.cc1 | C52 | 0.078 | 1.0 | 3 | 0:199, 2:675, 4:126 |
| No.11-C1.cc1 | C52 | 0.078 | 1.0 | 3 | 0:203, 2:661, 4:131, 6:5 |
| No.110-C2.cc1 | C52 | 0.047 | 1.0 | 2 | 0:221, 2:675, 4:103, 6:1 |
| No.111-C1.cc1 | C52 | 0.062 | 1.0 | 2 | 0:220, 2:670, 4:109, 6:1 |
| No.112-C1.cc1 | C52 | 0.063 | 1.0 | 2 | 0:212, 2:668, 4:119, 6:1 |
| No.113-C1.cc1 | C52 | 0.047 | 1.0 | 2 | 0:239, 2:654, 4:107 |
| No.114-C2.cc1 | C52 | 0.079 | 1.0 | 2 | 0:213, 2:617, 4:168, 6:2 |
| No.115-C1.cc1 | C52 | 0.063 | 1.0 | 2 | 0:230, 2:623, 4:146, 6:1 |
| No.116-C2.cc1 | C52 | 0.078 | 1.0 | 3 | 0:213, 2:670, 4:114, 6:3 |
| No.117-C1.cc1 | C52 | 0.062 | 1.0 | 2 | 0:208, 2:664, 4:126, 6:2 |
| No.118-C1.cc1 | C52 | 0.063 | 1.0 | 2 | 0:219, 2:674, 4:103, 6:4 |
| No.119-C1.cc1 | C52 | 0.062 | 1.0 | 2 | 0:217, 2:660, 4:122, 6:1 |
| No.12-C1.cc1 | C52 | 0.125 | 1.0 | 3 | 0:197, 2:663, 4:140 |
| No.120-Cs.cc1 | C52 | 0.172 | 1.0 | 2 | 0:231, 2:656, 4:111, 6:2 |
| No.121-C1.cc1 | C52 | 0.110 | 1.0 | 2 | 0:215, 2:671, 4:113, 6:1 |
| No.122-C1.cc1 | C52 | 0.156 | 1.0 | 2 | 0:217, 2:668, 4:115 |
| No.123-C1.cc1 | C52 | 0.140 | 1.0 | 2 | 0:239, 2:650, 4:109, 6:2 |
| No.124-C1.cc1 | C52 | 0.172 | 1.0 | 2 | 0:232, 2:652, 4:113, 6:3 |
| No.125-C2.cc1 | C52 | 0.125 | 1.0 | 2 | 0:218, 2:663, 4:117, 6:2 |
| No.126-C1.cc1 | C52 | 0.156 | 1.0 | 2 | 0:235, 2:644, 4:119, 6:2 |
| No.127-C2.cc1 | C52 | 0.125 | 1.0 | 3 | 0:226, 2:642, 4:131, 6:1 |
| No.128-C1.cc1 | C52 | 0.125 | 1.0 | 2 | 0:211, 2:655, 4:134 |
| No.129-C2.cc1 | C52 | 0.172 | 1.0 | 2 | 0:233, 2:656, 4:110, 6:1 |
| No.13-C1.cc1 | C52 | 0.125 | 1.0 | 3 | 0:202, 2:661, 4:137 |
| No.130-C1.cc1 | C52 | 0.125 | 1.0 | 2 | 0:251, 2:653, 4:95, 6:1 |
| No.131-C1.cc1 | C52 | 0.140 | 1.0 | 2 | 0:243, 2:670, 4:87 |
| No.132-C1.cc1 | C52 | 0.125 | 1.0 | 2 | 0:235, 2:675, 4:89, 6:1 |
| No.133-C1.cc1 | C52 | 0.109 | 1.0 | 2 | 0:249, 2:646, 4:104, 6:1 |
| No.134-C1.cc1 | C52 | 0.093 | 1.0 | 2 | 0:240, 2:672, 4:87, 6:1 |
| No.135-C1.cc1 | C52 | 0.079 | 1.0 | 2 | 0:264, 2:641, 4:95 |
| No.136-C1.cc1 | C52 | 0.063 | 1.0 | 2 | 0:234, 2:674, 4:92 |
| No.137-C1.cc1 | C52 | 0.063 | 1.0 | 2 | 0:236, 2:677, 4:87 |
| No.138-C2.cc1 | C52 | 0.094 | 1.0 | 3 | 0:250, 2:655, 4:93, 6:2 |
| No.139-C1.cc1 | C52 | 0.171 | 1.0 | 2 | 0:269, 2:654, 4:77 |
| No.14-C1.cc1 | C52 | 0.110 | 1.0 | 2 | 0:177, 2:678, 4:144, 6:1 |
| No.140-C1.cc1 | C52 | 0.079 | 1.0 | 2 | 0:265, 2:656, 4:78, 6:1 |
| No.141-C1.cc1 | C52 | 0.078 | 1.0 | 2 | 0:249, 2:651, 4:99, 6:1 |
| No.142-C1.cc1 | C52 | 0.078 | 1.0 | 2 | 0:254, 2:664, 4:82 |
| No.143-C1.cc1 | C52 | 0.079 | 1.0 | 2 | 0:293, 2:606, 4:100, 6:1 |
| No.144-C1.cc1 | C52 | 0.078 | 1.0 | 2 | 0:262, 2:650, 4:88 |
| No.145-C1.cc1 | C52 | 0.062 | 1.0 | 2 | 0:236, 2:662, 4:99, 6:3 |
| No.146-C1.cc1 | C52 | 0.063 | 1.0 | 2 | 0:271, 2:642, 4:87 |
| No.147-C1.cc1 | C52 | 0.062 | 1.0 | 2 | 0:219, 2:668, 4:112, 6:1 |
| No.148-C1.cc1 | C52 | 0.047 | 1.0 | 2 | 0:254, 2:660, 4:86 |
| No.149-C1.cc1 | C52 | 0.063 | 1.0 | 2 | 0:251, 2:668, 4:78, 6:3 |
| No.15-C2.cc1 | C52 | 0.046 | 1.0 | 3 | 0:190, 2:679, 4:130, 6:1 |
| No.150-C1.cc1 | C52 | 0.063 | 1.0 | 2 | 0:282, 2:639, 4:77, 6:2 |
| No.151-C1.cc1 | C52 | 0.047 | 1.0 | 2 | 0:249, 2:659, 4:92 |
| No.152-C2.cc1 | C52 | 0.047 | 1.0 | 2 | 0:245, 2:657, 4:97, 6:1 |
| No.153-C1.cc1 | C52 | 0.078 | 1.0 | 2 | 0:248, 2:671, 4:81 |
| No.154-C1.cc1 | C52 | 0.125 | 1.0 | 2 | 0:280, 2:648, 4:72 |
| No.155-C1.cc1 | C52 | 0.109 | 1.0 | 2 | 0:290, 2:658, 4:51, 6:1 |
| No.156-C1.cc1 | C52 | 0.094 | 1.0 | 3 | 0:253, 2:665, 4:80, 6:2 |
| No.157-C1.cc1 | C52 | 0.078 | 1.0 | 2 | 0:229, 2:683, 4:87, 6:1 |
| No.158-C1.cc1 | C52 | 0.062 | 1.0 | 2 | 0:235, 2:688, 4:77 |
| No.159-C1.cc1 | C52 | 0.110 | 1.0 | 2 | 0:258, 2:636, 4:102, 6:4 |
| No.16-C1.cc1 | C52 | 0.110 | 1.0 | 2 | 0:182, 2:657, 4:159, 6:2 |
| No.160-C1.cc1 | C52 | 0.079 | 1.0 | 2 | 0:235, 2:666, 4:99 |
| No.161-C1.cc1 | C52 | 0.110 | 1.0 | 2 | 0:266, 2:647, 4:87 |
| No.162-C2.cc1 | C52 | 0.140 | 1.0 | 2 | 0:231, 2:660, 4:107, 6:2 |
| No.163-C1.cc1 | C52 | 0.172 | 1.0 | 2 | 0:275, 2:657, 4:67, 6:1 |
| No.164-C1.cc1 | C52 | 0.172 | 1.0 | 2 | 0:266, 2:646, 4:87, 6:1 |
| No.165-C1.cc1 | C52 | 0.109 | 1.0 | 3 | 0:270, 2:654, 4:76 |
| No.166-C1.cc1 | C52 | 0.094 | 1.0 | 3 | 0:244, 2:670, 4:86 |
| No.167-C1.cc1 | C52 | 0.078 | 1.0 | 2 | 0:281, 2:655, 4:63, 6:1 |
| No.168-C1.cc1 | C52 | 0.078 | 1.0 | 2 | 0:271, 2:659, 4:68, 6:2 |
| No.169-C1.cc1 | C52 | 0.110 | 1.0 | 2 | 0:241, 2:674, 4:84, 6:1 |
| No.17-C1.cc1 | C52 | 0.156 | 1.0 | 2 | 0:202, 2:655, 4:141, 6:2 |
| No.170-C1.cc1 | C52 | 0.172 | 1.0 | 3 | 0:216, 2:691, 4:92, 6:1 |
| No.171-C2.cc1 | C52 | 0.125 | 1.0 | 2 | 0:264, 2:660, 4:76 |
| No.172-C1.cc1 | C52 | 0.157 | 1.0 | 2 | 0:254, 2:670, 4:74, 6:2 |
| No.173-C1.cc1 | C52 | 0.110 | 1.0 | 2 | 0:232, 2:680, 4:87, 6:1 |
| No.174-C1.cc1 | C52 | 0.093 | 1.0 | 2 | 0:281, 2:627, 4:89, 6:3 |
| No.175-C1.cc1 | C52 | 0.079 | 1.0 | 2 | 0:275, 2:646, 4:79 |
| No.176-C1.cc1 | C52 | 0.125 | 1.0 | 3 | 0:236, 2:647, 4:116, 6:1 |
| No.177-C1.cc1 | C52 | 0.156 | 1.0 | 2 | 0:252, 2:647, 4:101 |
| No.178-D2d.cc1 | C52 | 0.109 | 1.0 | 3 | 0:196, 2:645, 4:157, 6:2 |
| No.179-D2.cc1 | C52 | 0.094 | 1.0 | 4 | 0:232, 2:623, 4:143, 6:2 |
| No.18-C1.cc1 | C52 | 0.078 | 1.0 | 2 | 0:240, 2:666, 4:94 |
| No.180-C2.cc1 | C52 | 0.125 | 1.0 | 3 | 0:200, 2:681, 4:118, 6:1 |
| No.181-C1.cc1 | C52 | 0.172 | 1.0 | 2 | 0:211, 2:680, 4:108, 6:1 |
| No.182-C1.cc1 | C52 | 0.125 | 1.0 | 2 | 0:260, 2:655, 4:83, 6:2 |
| No.183-C1.cc1 | C52 | 0.110 | 1.0 | 2 | 0:229, 2:655, 4:114, 6:2 |
| No.184-C1.cc1 | C52 | 0.078 | 1.0 | 2 | 0:259, 2:655, 4:84, 6:2 |
| No.185-C1.cc1 | C52 | 0.063 | 1.0 | 2 | 0:249, 2:659, 4:92 |
| No.186-C1.cc1 | C52 | 0.063 | 1.0 | 2 | 0:218, 2:671, 4:110, 6:1 |
| No.187-C1.cc1 | C52 | 0.062 | 1.0 | 2 | 0:244, 2:655, 4:101 |
| No.188-C1.cc1 | C52 | 0.094 | 1.0 | 2 | 0:259, 2:662, 4:79 |
| No.189-C1.cc1 | C52 | 0.172 | 1.0 | 2 | 0:235, 2:659, 4:106 |
| No.19-C1.cc1 | C52 | 0.156 | 1.0 | 3 | 0:207, 2:694, 4:98, 6:1 |
| No.190-C1.cc1 | C52 | 0.125 | 1.0 | 2 | 0:172, 2:716, 4:109, 6:3 |
| No.191-C2.cc1 | C52 | 0.093 | 1.0 | 2 | 0:224, 2:655, 4:120, 6:1 |
| No.192-C1.cc1 | C52 | 0.078 | 1.0 | 2 | 0:256, 2:650, 4:93, 6:1 |
| No.193-C1.cc1 | C52 | 0.078 | 1.0 | 3 | 0:227, 2:683, 4:89, 6:1 |
| No.194-C1.cc1 | C52 | 0.109 | 1.0 | 2 | 0:237, 2:682, 4:81 |
| No.195-C1.cc1 | C52 | 0.156 | 1.0 | 2 | 0:218, 2:671, 4:110, 6:1 |
| No.196-C1.cc1 | C52 | 0.125 | 1.0 | 3 | 0:208, 2:660, 4:132 |
| No.197-C2.cc1 | C52 | 0.172 | 1.0 | 2 | 0:225, 2:653, 4:121, 6:1 |
| No.198-C1.cc1 | C52 | 0.141 | 1.0 | 2 | 0:257, 2:648, 4:92, 6:3 |
| No.199-C1.cc1 | C52 | 0.125 | 1.0 | 2 | 0:259, 2:643, 4:98 |
| No.2-D2.cc1 | C52 | 0.110 | 1.0 | 3 | 0:190, 2:655, 4:154, 6:1 |
| No.20-C1.cc1 | C52 | 0.094 | 1.0 | 3 | 0:202, 2:697, 4:100, 6:1 |
| No.200-C1.cc1 | C52 | 0.078 | 1.0 | 2 | 0:288, 2:627, 4:83, 6:2 |
| No.201-C1.cc1 | C52 | 0.078 | 1.0 | 2 | 0:261, 2:656, 4:83 |
| No.202-C1.cc1 | C52 | 0.125 | 1.0 | 3 | 0:258, 2:659, 4:82, 6:1 |
| No.203-C1.cc1 | C52 | 0.109 | 1.0 | 2 | 0:248, 2:659, 4:92, 6:1 |
| No.204-C1.cc1 | C52 | 0.125 | 1.0 | 2 | 0:247, 2:684, 4:69 |
| No.205-C1.cc1 | C52 | 0.109 | 1.0 | 2 | 0:256, 2:670, 4:73, 6:1 |
| No.206-C2.cc1 | C52 | 0.078 | 1.0 | 2 | 0:219, 2:695, 4:86 |
| No.207-C1.cc1 | C52 | 0.062 | 1.0 | 2 | 0:215, 2:702, 4:83 |
| No.208-C1.cc1 | C52 | 0.062 | 1.0 | 2 | 0:255, 2:678, 4:67 |
| No.209-C1.cc1 | C52 | 0.063 | 1.0 | 2 | 0:281, 2:646, 4:72, 6:1 |
| No.21-Cs.cc1 | C52 | 0.093 | 1.0 | 3 | 0:199, 2:692, 4:109 |
| No.210-C2.cc1 | C52 | 0.141 | 1.0 | 2 | 0:231, 2:696, 4:72, 6:1 |
| No.211-C1.cc1 | C52 | 0.109 | 1.0 | 2 | 0:240, 2:675, 4:85 |
| No.212-C1.cc1 | C52 | 0.078 | 1.0 | 2 | 0:274, 2:655, 4:71 |
| No.213-C1.cc1 | C52 | 0.078 | 1.0 | 2 | 0:307, 2:630, 4:62, 6:1 |
| No.214-C1.cc1 | C52 | 0.125 | 1.0 | 2 | 0:271, 2:637, 4:91, 6:1 |
| No.215-C1.cc1 | C52 | 0.171 | 1.0 | 2 | 0:251, 2:674, 4:73, 6:2 |
| No.216-C1.cc1 | C52 | 0.141 | 1.0 | 3 | 0:218, 2:688, 4:93, 6:1 |
| No.217-C1.cc1 | C52 | 0.109 | 1.0 | 2 | 0:249, 2:689, 4:62 |
| No.218-C1.cc1 | C52 | 0.094 | 1.0 | 2 | 0:256, 2:672, 4:72 |
| No.219-C1.cc1 | C52 | 0.078 | 1.0 | 2 | 0:264, 2:666, 4:70 |
| No.22-C2.cc1 | C52 | 0.078 | 1.0 | 2 | 0:172, 2:683, 4:141, 6:4 |
| No.220-C1.cc1 | C52 | 0.063 | 1.0 | 2 | 0:230, 2:669, 4:101 |
| No.221-Cs.cc1 | C52 | 0.078 | 1.0 | 2 | 0:292, 2:647, 4:60, 6:1 |
| No.222-C1.cc1 | C52 | 0.063 | 1.0 | 3 | 0:270, 2:645, 4:85 |
| No.223-C1.cc1 | C52 | 0.047 | 1.0 | 2 | 0:254, 2:673, 4:72, 6:1 |
| No.224-C1.cc1 | C52 | 0.078 | 1.0 | 2 | 0:265, 2:668, 4:67 |
| No.225-Cs.cc1 | C52 | 0.172 | 1.0 | 2 | 0:294, 2:632, 4:74 |
| No.226-C1.cc1 | C52 | 0.125 | 1.0 | 3 | 0:268, 2:653, 4:79 |
| No.227-C1.cc1 | C52 | 0.093 | 1.0 | 3 | 0:231, 2:703, 4:66 |
| No.228-C1.cc1 | C52 | 0.141 | 1.0 | 2 | 0:241, 2:681, 4:78 |
| No.229-Cs.cc1 | C52 | 0.156 | 1.0 | 2 | 0:249, 2:676, 4:74, 6:1 |
| No.23-C1.cc1 | C52 | 0.140 | 1.0 | 2 | 0:215, 2:645, 4:139, 6:1 |
| No.230-C1.cc1 | C52 | 0.156 | 1.0 | 2 | 0:240, 2:659, 4:99, 6:2 |
| No.231-C1.cc1 | C52 | 0.109 | 1.0 | 2 | 0:205, 2:697, 4:98 |
| No.232-C1.cc1 | C52 | 0.141 | 1.0 | 2 | 0:243, 2:674, 4:79, 6:4 |
| No.233-C1.cc1 | C52 | 0.156 | 1.0 | 2 | 0:251, 2:660, 4:89 |
| No.234-C1.cc1 | C52 | 0.172 | 1.0 | 3 | 0:220, 2:684, 4:94, 6:2 |
| No.235-C1.cc1 | C52 | 0.125 | 1.0 | 2 | 0:204, 2:681, 4:113, 6:2 |
| No.236-C1.cc1 | C52 | 0.140 | 1.0 | 2 | 0:240, 2:655, 4:105 |
| No.237-C3.cc1 | C52 | 0.110 | 1.0 | 3 | 0:195, 2:693, 4:111, 6:1 |
| No.238-C1.cc1 | C52 | 0.094 | 1.0 | 2 | 0:219, 2:698, 4:82, 6:1 |
| No.239-C2.cc1 | C52 | 0.078 | 1.0 | 2 | 0:260, 2:659, 4:80, 6:1 |
| No.24-C1.cc1 | C52 | 0.062 | 1.0 | 2 | 0:255, 2:641, 4:101, 6:3 |
| No.240-C1.cc1 | C52 | 0.062 | 1.0 | 2 | 0:283, 2:619, 4:96, 6:2 |
| No.241-Cs.cc1 | C52 | 0.047 | 1.0 | 2 | 0:290, 2:630, 4:79, 6:1 |
| No.242-C2v.cc1 | C52 | 0.063 | 1.0 | 2 | 0:286, 2:639, 4:74, 6:1 |
| No.243-C1.cc1 | C52 | 0.062 | 1.0 | 2 | 0:287, 2:632, 4:81 |
| No.244-C2.cc1 | C52 | 0.047 | 1.0 | 3 | 0:318, 2:613, 4:69 |
| No.245-C1.cc1 | C52 | 0.078 | 1.0 | 2 | 0:290, 2:652, 4:58 |
| No.246-C1.cc1 | C52 | 0.094 | 1.0 | 2 | 0:284, 2:628, 4:87, 6:1 |
| No.247-C1.cc1 | C52 | 0.110 | 1.0 | 2 | 0:246, 2:657, 4:96, 6:1 |
| No.248-C2.cc1 | C52 | 0.094 | 1.0 | 2 | 0:281, 2:653, 4:65, 6:1 |
| No.249-C1.cc1 | C52 | 0.078 | 1.0 | 2 | 0:296, 2:626, 4:77, 6:1 |
| No.25-C1.cc1 | C52 | 0.062 | 1.0 | 2 | 0:216, 2:664, 4:117, 6:3 |
| No.250-C1.cc1 | C52 | 0.078 | 1.0 | 2 | 0:257, 2:659, 4:83, 6:1 |
| No.251-C1.cc1 | C52 | 0.157 | 1.0 | 3 | 0:287, 2:661, 4:51, 6:1 |
| No.252-C1.cc1 | C52 | 0.156 | 1.0 | 2 | 0:283, 2:664, 4:53 |
| No.253-C1.cc1 | C52 | 0.156 | 1.0 | 2 | 0:256, 2:645, 4:98, 6:1 |
| No.254-C2.cc1 | C52 | 0.109 | 1.0 | 2 | 0:272, 2:668, 4:60 |
| No.255-C1.cc1 | C52 | 0.094 | 1.0 | 2 | 0:277, 2:661, 4:61, 6:1 |
| No.256-C1.cc1 | C52 | 0.125 | 1.0 | 2 | 0:285, 2:648, 4:67 |
| No.257-C2.cc1 | C52 | 0.141 | 1.0 | 3 | 0:241, 2:664, 4:95 |
| No.258-C1.cc1 | C52 | 0.172 | 1.0 | 3 | 0:253, 2:679, 4:68 |
| No.259-C1.cc1 | C52 | 0.171 | 1.0 | 2 | 0:248, 2:692, 4:59, 6:1 |
| No.26-C1.cc1 | C52 | 0.125 | 1.0 | 2 | 0:257, 2:650, 4:92, 6:1 |
| No.260-C1.cc1 | C52 | 0.172 | 1.0 | 2 | 0:285, 2:657, 4:57, 6:1 |
| No.261-C1.cc1 | C52 | 0.109 | 1.0 | 2 | 0:309, 2:640, 4:50, 6:1 |
| No.262-C1.cc1 | C52 | 0.093 | 1.0 | 2 | 0:285, 2:653, 4:61, 6:1 |
| No.263-C1.cc1 | C52 | 0.078 | 1.0 | 2 | 0:291, 2:652, 4:57 |
| No.264-C1.cc1 | C52 | 0.078 | 1.0 | 2 | 0:281, 2:643, 4:76 |
| No.265-C2.cc1 | C52 | 0.063 | 1.0 | 2 | 0:299, 2:641, 4:59, 6:1 |
| No.266-C1.cc1 | C52 | 0.093 | 1.0 | 3 | 0:310, 2:635, 4:55 |
| No.267-C2.cc1 | C52 | 0.203 | 1.0 | 2 | 0:255, 2:675, 4:69, 6:1 |
| No.268-C1.cc1 | C52 | 0.125 | 1.0 | 2 | 0:300, 2:652, 4:48 |
| No.269-C1.cc1 | C52 | 0.109 | 1.0 | 3 | 0:288, 2:651, 4:61 |
| No.27-C1.cc1 | C52 | 0.078 | 1.0 | 2 | 0:211, 2:672, 4:115, 6:2 |
| No.270-C1.cc1 | C52 | 0.062 | 1.0 | 2 | 0:249, 2:678, 4:73 |
| No.271-C1.cc1 | C52 | 0.063 | 1.0 | 2 | 0:314, 2:622, 4:64 |
| No.272-C1.cc1 | C52 | 0.047 | 1.0 | 2 | 0:271, 2:652, 4:77 |
| No.273-C1.cc1 | C52 | 0.063 | 1.0 | 2 | 0:307, 2:641, 4:52 |
| No.274-C1.cc1 | C52 | 0.062 | 1.0 | 2 | 0:268, 2:647, 4:85 |
| No.275-C1.cc1 | C52 | 0.078 | 1.0 | 2 | 0:216, 2:692, 4:90, 6:2 |
| No.276-C1.cc1 | C52 | 0.063 | 1.0 | 2 | 0:258, 2:674, 4:68 |
| No.277-C1.cc1 | C52 | 0.047 | 1.0 | 2 | 0:292, 2:642, 4:66 |
| No.278-C2.cc1 | C52 | 0.062 | 1.0 | 2 | 0:285, 2:653, 4:62 |
| No.279-C1.cc1 | C52 | 0.094 | 1.0 | 2 | 0:255, 2:667, 4:77, 6:1 |
| No.28-C1.cc1 | C52 | 0.157 | 1.0 | 2 | 0:202, 2:664, 4:131, 6:3 |
| No.280-C1.cc1 | C52 | 0.125 | 1.0 | 2 | 0:232, 2:689, 4:79 |
| No.281-C1.cc1 | C52 | 0.125 | 1.0 | 2 | 0:298, 2:636, 4:64, 6:2 |
| No.282-C1.cc1 | C52 | 0.125 | 1.0 | 2 | 0:232, 2:705, 4:63 |
| No.283-C2.cc1 | C52 | 0.110 | 1.0 | 2 | 0:305, 2:626, 4:68, 6:1 |
| No.284-C1.cc1 | C52 | 0.093 | 1.0 | 3 | 0:257, 2:671, 4:72 |
| No.285-C1.cc1 | C52 | 0.079 | 1.0 | 2 | 0:266, 2:653, 4:80, 6:1 |
| No.286-C1.cc1 | C52 | 0.062 | 1.0 | 2 | 0:204, 2:695, 4:99, 6:2 |
| No.287-C1.cc1 | C52 | 0.078 | 1.0 | 2 | 0:301, 2:644, 4:55 |
| No.288-C1.cc1 | C52 | 0.172 | 1.0 | 3 | 0:272, 2:671, 4:56, 6:1 |
| No.289-C1.cc1 | C52 | 0.157 | 1.0 | 2 | 0:239, 2:676, 4:85 |
| No.29-C2.cc1 | C52 | 0.110 | 1.0 | 2 | 0:231, 2:657, 4:112 |
| No.290-C1.cc1 | C52 | 0.125 | 1.0 | 2 | 0:261, 2:670, 4:69 |
| No.291-C1.cc1 | C52 | 0.156 | 1.0 | 2 | 0:242, 2:684, 4:73, 6:1 |
| No.292-C1.cc1 | C52 | 0.156 | 1.0 | 2 | 0:250, 2:671, 4:78, 6:1 |
| No.293-C2.cc1 | C52 | 0.172 | 1.0 | 2 | 0:293, 2:641, 4:65, 6:1 |
| No.294-Cs.cc1 | C52 | 0.109 | 1.0 | 2 | 0:244, 2:675, 4:80, 6:1 |
| No.295-C1.cc1 | C52 | 0.078 | 1.0 | 2 | 0:286, 2:632, 4:82 |
| No.296-C3v.cc1 | C52 | 0.078 | 1.0 | 2 | 0:297, 2:644, 4:58, 6:1 |
| No.297-C1.cc1 | C52 | 0.109 | 1.0 | 2 | 0:280, 2:634, 4:85, 6:1 |
| No.298-C1.cc1 | C52 | 0.078 | 1.0 | 2 | 0:254, 2:672, 4:74 |
| No.299-C1.cc1 | C52 | 0.063 | 1.0 | 2 | 0:298, 2:628, 4:74 |
| No.3-Cs.cc1 | C52 | 0.062 | 1.0 | 3 | 0:201, 2:644, 4:150, 6:5 |
| No.30-C1.cc1 | C52 | 0.063 | 1.0 | 2 | 0:227, 2:660, 4:109, 6:4 |
| No.300-C1.cc1 | C52 | 0.062 | 1.0 | 2 | 0:226, 2:684, 4:88, 6:2 |
| No.301-C1.cc1 | C52 | 0.156 | 1.0 | 2 | 0:253, 2:660, 4:87 |
| No.302-C1.cc1 | C52 | 0.109 | 1.0 | 2 | 0:265, 2:677, 4:58 |
| No.303-C3.cc1 | C52 | 0.141 | 1.0 | 2 | 0:248, 2:659, 4:91, 6:2 |
| No.304-C1.cc1 | C52 | 0.109 | 1.0 | 3 | 0:256, 2:666, 4:77, 6:1 |
| No.305-C1.cc1 | C52 | 0.078 | 1.0 | 2 | 0:227, 2:685, 4:88 |
| No.306-C1.cc1 | C52 | 0.140 | 1.0 | 2 | 0:270, 2:661, 4:68, 6:1 |
| No.307-C1.cc1 | C52 | 0.172 | 1.0 | 2 | 0:265, 2:675, 4:60 |
| No.308-C1.cc1 | C52 | 0.110 | 1.0 | 2 | 0:271, 2:650, 4:78, 6:1 |
| No.309-C1.cc1 | C52 | 0.094 | 1.0 | 2 | 0:260, 2:669, 4:70, 6:1 |
| No.31-C2.cc1 | C52 | 0.078 | 1.0 | 3 | 0:210, 2:651, 4:137, 6:2 |
| No.310-C1.cc1 | C52 | 0.078 | 1.0 | 3 | 0:260, 2:655, 4:84, 6:1 |
| No.311-C2.cc1 | C52 | 0.078 | 1.0 | 2 | 0:244, 2:673, 4:83 |
| No.312-C2.cc1 | C52 | 0.157 | 1.0 | 3 | 0:225, 2:690, 4:85 |
| No.313-D2d.cc1 | C52 | 0.110 | 1.0 | 3 | 0:184, 2:732, 4:84 |
| No.314-Cs.cc1 | C52 | 0.140 | 1.0 | 2 | 0:250, 2:671, 4:78, 6:1 |
| No.315-C2.cc1 | C52 | 0.156 | 1.0 | 2 | 0:254, 2:640, 4:104, 6:2 |
| No.316-C1.cc1 | C52 | 0.109 | 1.0 | 2 | 0:247, 2:667, 4:86 |
| No.317-C1.cc1 | C52 | 0.094 | 1.0 | 2 | 0:260, 2:656, 4:83, 6:1 |
| No.318-C1.cc1 | C52 | 0.078 | 1.0 | 2 | 0:255, 2:665, 4:80 |
| No.319-Cs.cc1 | C52 | 0.062 | 1.0 | 2 | 0:256, 2:651, 4:90, 6:3 |
| No.32-C1.cc1 | C52 | 0.078 | 1.0 | 2 | 0:181, 2:694, 4:123, 6:2 |
| No.320-C1.cc1 | C52 | 0.063 | 1.0 | 3 | 0:274, 2:625, 4:99, 6:2 |
| No.321-C1.cc1 | C52 | 0.062 | 1.0 | 2 | 0:265, 2:646, 4:89 |
| No.322-C1.cc1 | C52 | 0.157 | 1.0 | 2 | 0:279, 2:627, 4:92, 6:2 |
| No.323-C2.cc1 | C52 | 0.125 | 1.0 | 2 | 0:249, 2:669, 4:82 |
| No.324-C1.cc1 | C52 | 0.125 | 1.0 | 2 | 0:264, 2:637, 4:99 |
| No.325-C1.cc1 | C52 | 0.156 | 1.0 | 2 | 0:271, 2:655, 4:74 |
| No.326-C1.cc1 | C52 | 0.125 | 1.0 | 2 | 0:254, 2:678, 4:68 |
| No.327-C1.cc1 | C52 | 0.094 | 1.0 | 2 | 0:302, 2:636, 4:60, 6:2 |
| No.328-C1.cc1 | C52 | 0.078 | 1.0 | 2 | 0:315, 2:624, 4:61 |
| No.329-C1.cc1 | C52 | 0.062 | 1.0 | 2 | 0:264, 2:667, 4:69 |
| No.33-C1.cc1 | C52 | 0.062 | 1.0 | 2 | 0:229, 2:669, 4:100, 6:2 |
| No.330-C1.cc1 | C52 | 0.063 | 1.0 | 2 | 0:322, 2:631, 4:47 |
| No.331-C1.cc1 | C52 | 0.109 | 1.0 | 2 | 0:239, 2:668, 4:91, 6:2 |
| No.332-C1.cc1 | C52 | 0.125 | 1.0 | 2 | 0:273, 2:657, 4:70 |
| No.333-D2.cc1 | C52 | 0.140 | 1.0 | 2 | 0:290, 2:638, 4:72 |
| No.334-C2.cc1 | C52 | 0.156 | 1.0 | 2 | 0:229, 2:672, 4:99 |
| No.335-C2.cc1 | C52 | 0.109 | 1.0 | 2 | 0:300, 2:630, 4:70 |
| No.336-C2.cc1 | C52 | 0.078 | 1.0 | 2 | 0:285, 2:630, 4:84, 6:1 |
| No.337-C1.cc1 | C52 | 0.078 | 1.0 | 2 | 0:220, 2:692, 4:87, 6:1 |
| No.338-C1.cc1 | C52 | 0.125 | 1.0 | 2 | 0:304, 2:623, 4:73 |
| No.339-C2.cc1 | C52 | 0.172 | 1.0 | 2 | 0:287, 2:625, 4:88 |
| No.34-C1.cc1 | C52 | 0.125 | 1.0 | 2 | 0:221, 2:670, 4:106, 6:3 |
| No.340-C2.cc1 | C52 | 0.093 | 1.0 | 2 | 0:299, 2:636, 4:65 |
| No.341-C1.cc1 | C52 | 0.094 | 1.0 | 2 | 0:268, 2:653, 4:78, 6:1 |
| No.342-C1.cc1 | C52 | 0.078 | 1.0 | 2 | 0:221, 2:687, 4:92 |
| No.343-C1.cc1 | C52 | 0.078 | 1.0 | 2 | 0:274, 2:663, 4:62, 6:1 |
| No.344-C1.cc1 | C52 | 0.063 | 1.0 | 2 | 0:270, 2:643, 4:83, 6:4 |
| No.345-C1.cc1 | C52 | 0.047 | 1.0 | 2 | 0:236, 2:655, 4:108, 6:1 |
| No.346-C2.cc1 | C52 | 0.125 | 1.0 | 2 | 0:257, 2:659, 4:84 |
| No.347-Cs.cc1 | C52 | 0.140 | 1.0 | 2 | 0:222, 2:678, 4:100 |
| No.348-C2.cc1 | C52 | 0.094 | 1.0 | 2 | 0:230, 2:673, 4:97 |
| No.349-C1.cc1 | C52 | 0.094 | 1.0 | 2 | 0:242, 2:679, 4:79 |
| No.35-C1.cc1 | C52 | 0.109 | 1.0 | 2 | 0:213, 2:688, 4:97, 6:2 |
| No.350-C1.cc1 | C52 | 0.109 | 1.0 | 2 | 0:271, 2:635, 4:94 |
| No.351-C1.cc1 | C52 | 0.172 | 1.0 | 3 | 0:276, 2:649, 4:75 |
| No.352-C1.cc1 | C52 | 0.125 | 1.0 | 2 | 0:267, 2:642, 4:89, 6:2 |
| No.353-C1.cc1 | C52 | 0.094 | 1.0 | 2 | 0:266, 2:654, 4:79, 6:1 |
| No.354-D2.cc1 | C52 | 0.110 | 1.0 | 3 | 0:248, 2:653, 4:99 |
| No.355-C2.cc1 | C52 | 0.172 | 1.0 | 2 | 0:232, 2:658, 4:110 |
| No.356-C1.cc1 | C52 | 0.109 | 1.0 | 3 | 0:248, 2:687, 4:63, 6:2 |
| No.357-C1.cc1 | C52 | 0.078 | 1.0 | 2 | 0:233, 2:672, 4:94, 6:1 |
| No.358-C1.cc1 | C52 | 0.079 | 1.0 | 2 | 0:239, 2:677, 4:84 |
| No.359-C2.cc1 | C52 | 0.078 | 1.0 | 2 | 0:281, 2:656, 4:62, 6:1 |
| No.36-C1.cc1 | C52 | 0.078 | 1.0 | 3 | 0:234, 2:663, 4:102, 6:1 |
| No.360-C1.cc1 | C52 | 0.063 | 1.0 | 2 | 0:252, 2:674, 4:73, 6:1 |
| No.361-C1.cc1 | C52 | 0.078 | 1.0 | 2 | 0:250, 2:664, 4:86 |
| No.362-C2.cc1 | C52 | 0.062 | 1.0 | 2 | 0:235, 2:664, 4:97, 6:4 |
| No.363-C2.cc1 | C52 | 0.047 | 1.0 | 3 | 0:250, 2:653, 4:95, 6:2 |
| No.364-C1.cc1 | C52 | 0.047 | 1.0 | 2 | 0:267, 2:669, 4:63, 6:1 |
| No.365-C1.cc1 | C52 | 0.062 | 1.0 | 2 | 0:253, 2:680, 4:67 |
| No.366-C1.cc1 | C52 | 0.047 | 1.0 | 2 | 0:255, 2:678, 4:67 |
| No.367-C1.cc1 | C52 | 0.062 | 1.0 | 2 | 0:250, 2:665, 4:85 |
| No.368-D2.cc1 | C52 | 0.094 | 1.0 | 2 | 0:208, 2:685, 4:104, 6:3 |
| No.369-C1.cc1 | C52 | 0.125 | 1.0 | 2 | 0:226, 2:679, 4:95 |
| No.37-C1.cc1 | C52 | 0.125 | 1.0 | 2 | 0:212, 2:676, 4:108, 6:4 |
| No.370-C1.cc1 | C52 | 0.172 | 1.0 | 3 | 0:270, 2:662, 4:68 |
| No.371-C1.cc1 | C52 | 0.157 | 1.0 | 2 | 0:290, 2:643, 4:67 |
| No.372-C2.cc1 | C52 | 0.110 | 1.0 | 3 | 0:292, 2:649, 4:58, 6:1 |
| No.373-C1.cc1 | C52 | 0.125 | 1.0 | 2 | 0:254, 2:671, 4:73, 6:2 |
| No.374-C1.cc1 | C52 | 0.156 | 1.0 | 2 | 0:229, 2:688, 4:82, 6:1 |
| No.375-C2.cc1 | C52 | 0.156 | 1.0 | 2 | 0:274, 2:646, 4:79, 6:1 |
| No.376-C2.cc1 | C52 | 0.109 | 1.0 | 3 | 0:271, 2:660, 4:68, 6:1 |
| No.377-C1.cc1 | C52 | 0.094 | 1.0 | 2 | 0:275, 2:660, 4:65 |
| No.378-C2.cc1 | C52 | 0.109 | 1.0 | 2 | 0:325, 2:616, 4:58, 6:1 |
| No.379-C1.cc1 | C52 | 0.094 | 1.0 | 2 | 0:303, 2:630, 4:67 |
| No.38-Cs.cc1 | C52 | 0.140 | 1.0 | 3 | 0:200, 2:687, 4:110, 6:3 |
| No.380-C2.cc1 | C52 | 0.172 | 1.0 | 2 | 0:294, 2:636, 4:69, 6:1 |
| No.381-C2.cc1 | C52 | 0.125 | 1.0 | 2 | 0:308, 2:622, 4:69, 6:1 |
| No.382-C1.cc1 | C52 | 0.125 | 1.0 | 2 | 0:239, 2:693, 4:68 |
| No.383-C2.cc1 | C52 | 0.125 | 1.0 | 2 | 0:257, 2:670, 4:72, 6:1 |
| No.384-C1.cc1 | C52 | 0.094 | 1.0 | 2 | 0:294, 2:642, 4:63, 6:1 |
| No.385-C2.cc1 | C52 | 0.078 | 1.0 | 2 | 0:235, 2:671, 4:91, 6:3 |
| No.386-C2.cc1 | C52 | 0.079 | 1.0 | 2 | 0:271, 2:663, 4:65, 6:1 |
| No.387-C1.cc1 | C52 | 0.078 | 1.0 | 3 | 0:224, 2:699, 4:74, 6:3 |
| No.388-C2.cc1 | C52 | 0.109 | 1.0 | 2 | 0:279, 2:642, 4:79 |
| No.389-C2h.cc1 | C52 | 0.125 | 1.0 | 2 | 0:234, 2:685, 4:81 |
| No.39-C1.cc1 | C52 | 0.156 | 1.0 | 2 | 0:229, 2:671, 4:97, 6:3 |
| No.390-Cs.cc1 | C52 | 0.172 | 1.0 | 2 | 0:269, 2:645, 4:85, 6:1 |
| No.391-C2v.cc1 | C52 | 0.125 | 1.0 | 2 | 0:255, 2:656, 4:89 |
| No.392-Cs.cc1 | C52 | 0.094 | 1.0 | 3 | 0:310, 2:614, 4:75, 6:1 |
| No.393-C2.cc1 | C52 | 0.141 | 1.0 | 2 | 0:251, 2:656, 4:91, 6:2 |
| No.394-D2.cc1 | C52 | 0.109 | 1.0 | 2 | 0:196, 2:705, 4:99 |
| No.395-C2.cc1 | C52 | 0.156 | 1.0 | 2 | 0:249, 2:687, 4:63, 6:1 |
| No.396-C1.cc1 | C52 | 0.156 | 1.0 | 2 | 0:275, 2:656, 4:68, 6:1 |
| No.397-C2.cc1 | C52 | 0.156 | 1.0 | 2 | 0:262, 2:671, 4:66, 6:1 |
| No.398-C1.cc1 | C52 | 0.125 | 1.0 | 2 | 0:298, 2:630, 4:71, 6:1 |
| No.399-C2.cc1 | C52 | 0.172 | 1.0 | 2 | 0:270, 2:657, 4:72, 6:1 |
| No.4-C1.cc1 | C52 | 0.156 | 1.0 | 2 | 0:211, 2:654, 4:134, 6:1 |
| No.40-Cs.cc1 | C52 | 0.109 | 1.0 | 2 | 0:240, 2:660, 4:99, 6:1 |
| No.400-Cs.cc1 | C52 | 0.094 | 1.0 | 2 | 0:256, 2:671, 4:73 |
| No.401-C1.cc1 | C52 | 0.109 | 1.0 | 2 | 0:268, 2:666, 4:66 |
| No.402-C1.cc1 | C52 | 0.125 | 1.0 | 2 | 0:290, 2:639, 4:69, 6:2 |
| No.403-C1.cc1 | C52 | 0.156 | 1.0 | 3 | 0:261, 2:663, 4:76 |
| No.404-C1.cc1 | C52 | 0.156 | 1.0 | 2 | 0:286, 2:652, 4:59, 6:3 |
| No.405-C1.cc1 | C52 | 0.125 | 1.0 | 2 | 0:270, 2:673, 4:57 |
| No.406-C1.cc1 | C52 | 0.125 | 1.0 | 2 | 0:234, 2:682, 4:84 |
| No.407-C1.cc1 | C52 | 0.110 | 1.0 | 2 | 0:246, 2:689, 4:65 |
| No.408-C1.cc1 | C52 | 0.172 | 1.0 | 2 | 0:263, 2:661, 4:73, 6:3 |
| No.409-C1.cc1 | C52 | 0.125 | 1.0 | 2 | 0:282, 2:647, 4:69, 6:2 |
| No.41-C1.cc1 | C52 | 0.094 | 1.0 | 2 | 0:225, 2:662, 4:113 |
| No.410-Cs.cc1 | C52 | 0.078 | 1.0 | 2 | 0:255, 2:672, 4:73 |
| No.411-Cs.cc1 | C52 | 0.078 | 1.0 | 2 | 0:324, 2:613, 4:63 |
| No.412-C1.cc1 | C52 | 0.125 | 1.0 | 2 | 0:265, 2:652, 4:82, 6:1 |
| No.413-C2.cc1 | C52 | 0.141 | 1.0 | 2 | 0:248, 2:647, 4:104, 6:1 |
| No.414-C1.cc1 | C52 | 0.109 | 1.0 | 2 | 0:281, 2:638, 4:81 |
| No.415-C1.cc1 | C52 | 0.109 | 1.0 | 2 | 0:270, 2:658, 4:70, 6:2 |
| No.416-C1.cc1 | C52 | 0.094 | 1.0 | 2 | 0:318, 2:638, 4:44 |
| No.417-C2.cc1 | C52 | 0.141 | 1.0 | 2 | 0:313, 2:627, 4:60 |
| No.418-C1.cc1 | C52 | 0.157 | 1.0 | 2 | 0:315, 2:630, 4:55 |
| No.419-C2.cc1 | C52 | 0.172 | 1.0 | 2 | 0:317, 2:623, 4:60 |
| No.42-C1.cc1 | C52 | 0.125 | 1.0 | 2 | 0:216, 2:669, 4:114, 6:1 |
| No.420-C2.cc1 | C52 | 0.125 | 1.0 | 2 | 0:308, 2:641, 4:51 |
| No.421-C2.cc1 | C52 | 0.094 | 1.0 | 2 | 0:276, 2:669, 4:55 |
| No.422-C2.cc1 | C52 | 0.094 | 1.0 | 2 | 0:287, 2:653, 4:60 |
| No.423-C1.cc1 | C52 | 0.078 | 1.0 | 2 | 0:281, 2:658, 4:59, 6:2 |
| No.424-C1.cc1 | C52 | 0.062 | 1.0 | 2 | 0:315, 2:628, 4:55, 6:2 |
| No.425-C3.cc1 | C52 | 0.062 | 1.0 | 2 | 0:284, 2:668, 4:48 |
| No.426-C1.cc1 | C52 | 0.047 | 1.0 | 2 | 0:252, 2:686, 4:62 |
| No.427-C2.cc1 | C52 | 0.063 | 1.0 | 3 | 0:294, 2:624, 4:81, 6:1 |
| No.428-D2.cc1 | C52 | 0.047 | 1.0 | 2 | 0:261, 2:668, 4:71 |
| No.429-C1.cc1 | C52 | 0.063 | 1.0 | 2 | 0:312, 2:638, 4:50 |
| No.43-Cs.cc1 | C52 | 0.078 | 1.0 | 2 | 0:257, 2:648, 4:95 |
| No.430-C1.cc1 | C52 | 0.156 | 1.0 | 2 | 0:283, 2:661, 4:56 |
| No.431-D2h.cc1 | C52 | 0.172 | 1.0 | 2 | 0:323, 2:600, 4:77 |
| No.432-D2d.cc1 | C52 | 0.125 | 1.0 | 2 | 0:296, 2:638, 4:66 |
| No.433-D2.cc1 | C52 | 0.125 | 1.0 | 3 | 0:292, 2:626, 4:82 |
| No.434-C2.cc1 | C52 | 0.156 | 1.0 | 3 | 0:300, 2:642, 4:57, 6:1 |
| No.435-D2d.cc1 | C52 | 0.125 | 1.0 | 2 | 0:272, 2:650, 4:78 |
| No.436-C1.cc1 | C52 | 0.093 | 1.0 | 2 | 0:296, 2:653, 4:51 |
| No.437-T.cc1 | C52 | 0.094 | 1.0 | 2 | 0:301, 2:645, 4:53, 6:1 |
| No.44-Cs.cc1 | C52 | 0.078 | 1.0 | 2 | 0:254, 2:653, 4:91, 6:2 |
| No.45-C1.cc1 | C52 | 0.078 | 1.0 | 2 | 0:243, 2:659, 4:98 |
| No.46-C1.cc1 | C52 | 0.062 | 1.0 | 2 | 0:267, 2:643, 4:88, 6:2 |
| No.47-C1.cc1 | C52 | 0.047 | 1.0 | 2 | 0:241, 2:641, 4:118 |
| No.48-C1.cc1 | C52 | 0.063 | 1.0 | 3 | 0:259, 2:630, 4:108, 6:3 |
| No.49-C1.cc1 | C52 | 0.047 | 1.0 | 3 | 0:265, 2:647, 4:88 |
| No.5-C2.cc1 | C52 | 0.047 | 1.0 | 2 | 0:179, 2:681, 4:137, 6:3 |
| No.50-C2.cc1 | C52 | 0.156 | 1.0 | 2 | 0:278, 2:642, 4:80 |
| No.51-C1.cc1 | C52 | 0.125 | 1.0 | 3 | 0:246, 2:660, 4:92, 6:2 |
| No.52-C1.cc1 | C52 | 0.109 | 1.0 | 2 | 0:234, 2:659, 4:107 |
| No.53-C1.cc1 | C52 | 0.079 | 1.0 | 2 | 0:219, 2:683, 4:98 |
| No.54-C1.cc1 | C52 | 0.110 | 1.0 | 2 | 0:219, 2:683, 4:95, 6:3 |
| No.55-C1.cc1 | C52 | 0.125 | 1.0 | 2 | 0:222, 2:689, 4:89 |
| No.56-C1.cc1 | C52 | 0.156 | 1.0 | 2 | 0:245, 2:647, 4:108 |
| No.57-C1.cc1 | C52 | 0.110 | 1.0 | 2 | 0:247, 2:666, 4:87 |
| No.58-D2.cc1 | C52 | 0.094 | 1.0 | 2 | 0:219, 2:686, 4:94, 6:1 |
| No.59-C1.cc1 | C52 | 0.078 | 1.0 | 2 | 0:250, 2:658, 4:91, 6:1 |
| No.6-Cs.cc1 | C52 | 0.110 | 1.0 | 3 | 0:200, 2:662, 4:134, 6:4 |
| No.60-C1.cc1 | C52 | 0.110 | 1.0 | 3 | 0:260, 2:647, 4:93 |
| No.61-C1.cc1 | C52 | 0.125 | 1.0 | 2 | 0:230, 2:680, 4:89, 6:1 |
| No.62-C1.cc1 | C52 | 0.172 | 1.0 | 3 | 0:247, 2:657, 4:95, 6:1 |
| No.63-C1.cc1 | C52 | 0.125 | 1.0 | 2 | 0:244, 2:660, 4:96 |
| No.64-C1.cc1 | C52 | 0.094 | 1.0 | 2 | 0:204, 2:697, 4:99 |
| No.65-C1.cc1 | C52 | 0.078 | 1.0 | 2 | 0:255, 2:658, 4:85, 6:2 |
| No.66-Cs.cc1 | C52 | 0.078 | 1.0 | 2 | 0:267, 2:652, 4:81 |
| No.67-Cs.cc1 | C52 | 0.078 | 1.0 | 2 | 0:251, 2:664, 4:84, 6:1 |
| No.68-C1.cc1 | C52 | 0.062 | 1.0 | 3 | 0:266, 2:650, 4:83, 6:1 |
| No.69-C1.cc1 | C52 | 0.078 | 1.0 | 3 | 0:242, 2:668, 4:89, 6:1 |
| No.7-C1.cc1 | C52 | 0.063 | 1.0 | 2 | 0:220, 2:654, 4:125, 6:1 |
| No.70-C1.cc1 | C52 | 0.063 | 1.0 | 3 | 0:263, 2:655, 4:82 |
| No.71-C1.cc1 | C52 | 0.062 | 1.0 | 2 | 0:230, 2:681, 4:89 |
| No.72-C1.cc1 | C52 | 0.063 | 1.0 | 2 | 0:238, 2:678, 4:84 |
| No.73-C1.cc1 | C52 | 0.062 | 1.0 | 3 | 0:247, 2:673, 4:80 |
| No.74-C1.cc1 | C52 | 0.047 | 1.0 | 2 | 0:271, 2:669, 4:59, 6:1 |
| No.75-C1.cc1 | C52 | 0.047 | 1.0 | 2 | 0:237, 2:669, 4:94 |
| No.76-C1.cc1 | C52 | 0.062 | 1.0 | 3 | 0:256, 2:659, 4:83, 6:2 |
| No.77-C1.cc1 | C52 | 0.047 | 1.0 | 2 | 0:208, 2:686, 4:104, 6:2 |
| No.78-C1.cc1 | C52 | 0.062 | 1.0 | 2 | 0:273, 2:640, 4:86, 6:1 |
| No.79-C1.cc1 | C52 | 0.063 | 1.0 | 2 | 0:219, 2:694, 4:87 |
| No.8-C1.cc1 | C52 | 0.062 | 1.0 | 3 | 0:216, 2:667, 4:114, 6:3 |
| No.80-C1.cc1 | C52 | 0.047 | 1.0 | 3 | 0:217, 2:698, 4:84, 6:1 |
| No.81-C1.cc1 | C52 | 0.063 | 1.0 | 3 | 0:254, 2:663, 4:83 |
| No.82-C2.cc1 | C52 | 0.046 | 1.0 | 2 | 0:227, 2:684, 4:86, 6:3 |
| No.83-C2.cc1 | C52 | 0.063 | 1.0 | 2 | 0:204, 2:662, 4:129, 6:5 |
| No.84-C2.cc1 | C52 | 0.125 | 1.0 | 2 | 0:266, 2:666, 4:67, 6:1 |
| No.85-C1.cc1 | C52 | 0.141 | 1.0 | 2 | 0:190, 2:677, 4:133 |
| No.86-C1.cc1 | C52 | 0.140 | 1.0 | 2 | 0:241, 2:674, 4:84, 6:1 |
| No.87-C1.cc1 | C52 | 0.094 | 1.0 | 2 | 0:241, 2:648, 4:111 |
| No.88-C2.cc1 | C52 | 0.140 | 1.0 | 3 | 0:209, 2:682, 4:108, 6:1 |
| No.89-C1.cc1 | C52 | 0.140 | 1.0 | 2 | 0:278, 2:639, 4:83 |
| No.9-C1.cc1 | C52 | 0.110 | 1.0 | 2 | 0:201, 2:626, 4:168, 6:5 |
| No.90-C1.cc1 | C52 | 0.094 | 1.0 | 2 | 0:260, 2:656, 4:83, 6:1 |
| No.91-C1.cc1 | C52 | 0.078 | 1.0 | 2 | 0:267, 2:637, 4:94, 6:2 |
| No.92-Cs.cc1 | C52 | 0.062 | 1.0 | 2 | 0:273, 2:648, 4:77, 6:2 |
| No.93-C1.cc1 | C52 | 0.047 | 1.0 | 2 | 0:266, 2:662, 4:72 |
| No.94-D2d.cc1 | C52 | 0.078 | 1.0 | 3 | 0:227, 2:681, 4:92 |
| No.95-Cs.cc1 | C52 | 0.079 | 1.0 | 2 | 0:256, 2:662, 4:80, 6:2 |
| No.96-C1.cc1 | C52 | 0.078 | 1.0 | 2 | 0:253, 2:674, 4:70, 6:3 |
| No.97-C1.cc1 | C52 | 0.063 | 1.0 | 2 | 0:242, 2:667, 4:89, 6:2 |
| No.98-C1.cc1 | C52 | 0.094 | 1.0 | 2 | 0:230, 2:684, 4:85, 6:1 |
| No.99-C2.cc1 | C52 | 0.062 | 1.0 | 2 | 0:237, 2:687, 4:75, 6:1 |
| C60-Ih.cc1 | C60 | 0.063 | 1.0 | 2 | 0:341, 2:589, 4:67, 6:3 |
| C70-D5h.cc1 | C70 | 0.062 | 1.0 | 2 | 0:249, 2:648, 4:102, 6:1 |
| C72-D6d.cc1 | C72 | 0.172 | 1.0 | 3 | 0:218, 2:663, 4:113, 6:6 |
| C74-D3h.cc1 | C74 | 0.203 | 1.0 | 2 | 0:278, 2:627, 4:91, 6:4 |
| C76-D2.cc1 | C76 | 0.125 | 1.0 | 2 | 0:219, 2:664, 4:116, 6:1 |
| C76-Td.cc1 | C76 | 0.110 | 1.0 | 2 | 0:218, 2:663, 4:114, 6:5 |
| No.1-D3.cc1 | C78 | 0.109 | 1.0 | 3 | 0:179, 2:668, 4:146, 6:7 |
| No.2-C2v.cc1 | C78 | 0.078 | 1.0 | 2 | 0:220, 2:649, 4:124, 6:7 |
| No.3-C2v.cc1 | C78 | 0.078 | 1.0 | 2 | 0:244, 2:625, 4:124, 6:7 |
| No.4-D3h.cc1 | C78 | 0.063 | 1.0 | 2 | 0:207, 2:630, 4:157, 6:6 |
| No.5-D3h.cc1 | C78 | 0.062 | 1.0 | 2 | 0:237, 2:649, 4:113, 6:1 |
| No.1-D5d.cc1 | C80 | 0.078 | 1.0 | 2 | 0:228, 2:622, 4:140, 6:10 |
| No.2-D2.cc1 | C80 | 0.172 | 1.0 | 3 | 0:225, 2:650, 4:120, 6:5 |
| No.3-C2v.cc1 | C80 | 0.218 | 1.0 | 2 | 0:214, 2:653, 4:124, 6:9 |
| No.4-D3.cc1 | C80 | 0.141 | 1.0 | 2 | 0:234, 2:625, 4:139, 6:2 |
| No.5-C2v.cc1 | C80 | 0.187 | 1.0 | 2 | 0:236, 2:653, 4:108, 6:3 |
| No.6-D5h.cc1 | C80 | 0.219 | 1.0 | 3 | 0:262, 2:621, 4:114, 6:3 |
| No.7-Ih.cc1 | C80 | 0.140 | 1.1 | 3 | 0:271, 2:632, 4:96, 6:1 |
| No.1-C2.cc1 | C82 | 0.109 | 1.0 | 2 | 0:224, 2:627, 4:146, 6:3 |
| No.2-Cs.cc1 | C82 | 0.156 | 1.0 | 2 | 0:195, 2:654, 4:147, 6:4 |
| No.3-C2.cc1 | C82 | 0.219 | 1.0 | 2 | 0:239, 2:623, 4:124, 6:14 |
| No.4-Cs.cc1 | C82 | 0.219 | 1.0 | 3 | 0:230, 2:661, 4:106, 6:3 |
| No.5-C2.cc1 | C82 | 0.141 | 1.0 | 3 | 0:247, 2:626, 4:120, 6:7 |
| No.6-Cs.cc1 | C82 | 0.110 | 1.0 | 3 | 0:264, 2:625, 4:107, 6:4 |
| No.7-C3v.cc1 | C82 | 0.109 | 1.0 | 2 | 0:229, 2:635, 4:130, 6:6 |
| No.8-C3v.cc1 | C82 | 0.078 | 1.1 | 3 | 0:257, 2:632, 4:107, 6:4 |
| No.9-C2v.cc1 | C82 | 0.094 | 1.0 | 3 | 0:246, 2:641, 4:110, 6:3 |
| No.1-D2.cc1 | C84 | 0.078 | 1.0 | 2 | 0:173, 2:635, 4:184, 6:8 |
| No.10-Cs.cc1 | C84 | 0.078 | 1.0 | 2 | 0:232, 2:624, 4:139, 6:5 |
| No.11-C2.cc1 | C84 | 0.078 | 1.0 | 2 | 0:223, 2:635, 4:136, 6:6 |
| No.12-C1.cc1 | C84 | 0.125 | 1.0 | 2 | 0:220, 2:635, 4:139, 6:6 |
| No.13-C2.cc1 | C84 | 0.187 | 1.0 | 3 | 0:225, 2:662, 4:105, 6:8 |
| No.14-Cs.cc1 | C84 | 0.235 | 1.0 | 4 | 0:248, 2:628, 4:118, 6:6 |
| No.15-Cs.cc1 | C84 | 0.219 | 1.0 | 3 | 0:221, 2:645, 4:129, 6:5 |
| No.16-Cs.cc1 | C84 | 0.156 | 1.0 | 2 | 0:250, 2:626, 4:122, 6:2 |
| No.17-C2v.cc1 | C84 | 0.125 | 1.0 | 3 | 0:207, 2:673, 4:116, 6:4 |
| No.18-C2v.cc1 | C84 | 0.110 | 1.0 | 2 | 0:242, 2:619, 4:133, 6:6 |
| No.19-D3d.cc1 | C84 | 0.094 | 1.0 | 2 | 0:248, 2:620, 4:130, 6:2 |
| No.2-C2.cc1 | C84 | 0.078 | 1.0 | 2 | 0:194, 2:627, 4:174, 6:5 |
| No.20-Td.cc1 | C84 | 0.203 | 1.0 | 2 | 0:210, 2:615, 4:164, 6:11 |
| No.21-D2.cc1 | C84 | 0.234 | 1.0 | 2 | 0:211, 2:647, 4:140, 6:2 |
| No.22-D2.cc1 | C84 | 0.219 | 1.0 | 3 | 0:248, 2:624, 4:127, 6:1 |
| No.23-D2d.cc1 | C84 | 0.141 | 1.0 | 2 | 0:231, 2:640, 4:126, 6:3 |
| No.24-D6h.cc1 | C84 | 0.219 | 1.0 | 4 | 0:253, 2:621, 4:124, 6:2 |
| No.3-Cs.cc1 | C84 | 0.172 | 1.0 | 2 | 0:210, 2:642, 4:141, 6:7 |
| No.4-D2d.cc1 | C84 | 0.219 | 1.0 | 2 | 0:203, 2:659, 4:131, 6:7 |
| No.5-D2.cc1 | C84 | 0.218 | 1.0 | 2 | 0:189, 2:655, 4:151, 6:5 |
| No.6-C2v.cc1 | C84 | 0.219 | 1.0 | 2 | 0:230, 2:621, 4:140, 6:9 |
| No.7-C2v.cc1 | C84 | 0.250 | 1.0 | 2 | 0:229, 2:640, 4:130, 6:1 |
| No.8-C2.cc1 | C84 | 0.188 | 1.0 | 2 | 0:216, 2:656, 4:125, 6:3 |
| No.9-C2.cc1 | C84 | 0.140 | 1.0 | 2 | 0:235, 2:635, 4:126, 6:4 |
| No.1-C1.cc1 | C86 | 0.172 | 1.0 | 3 | 0:177, 2:656, 4:160, 6:7 |
| No.10-C2v.cc1 | C86 | 0.140 | 1.0 | 2 | 0:237, 2:634, 4:122, 6:7 |
| No.11-C1.cc1 | C86 | 0.219 | 1.0 | 2 | 0:229, 2:610, 4:154, 6:6, 8:1 |
| No.12-C1.cc1 | C86 | 0.250 | 1.0 | 3 | 0:206, 2:647, 4:138, 6:9 |
| No.13-C1.cc1 | C86 | 0.250 | 1.0 | 2 | 0:204, 2:654, 4:136, 6:6 |
| No.14-C2.cc1 | C86 | 0.235 | 1.0 | 3 | 0:203, 2:652, 4:142, 6:3 |
| No.15-Cs.cc1 | C86 | 0.156 | 1.0 | 2 | 0:198, 2:653, 4:147, 6:2 |
| No.16-Cs.cc1 | C86 | 0.218 | 1.0 | 2 | 0:202, 2:626, 4:167, 6:5 |
| No.17-C2.cc1 | C86 | 0.218 | 1.0 | 3 | 0:203, 2:658, 4:134, 6:5 |
| No.18-C3.cc1 | C86 | 0.141 | 1.0 | 3 | 0:189, 2:662, 4:140, 6:9 |
| No.19-D3.cc1 | C86 | 0.125 | 1.0 | 3 | 0:223, 2:642, 4:128, 6:7 |
| No.2-C2.cc1 | C86 | 0.109 | 1.0 | 2 | 0:166, 2:621, 4:200, 6:12, 8:1 |
| No.3-C2.cc1 | C86 | 0.094 | 1.0 | 3 | 0:164, 2:659, 4:171, 6:6 |
| No.4-C2.cc1 | C86 | 0.078 | 1.0 | 2 | 0:200, 2:647, 4:147, 6:6 |
| No.5-C1.cc1 | C86 | 0.172 | 1.0 | 2 | 0:197, 2:652, 4:146, 6:5 |
| No.6-C2.cc1 | C86 | 0.234 | 1.0 | 2 | 0:183, 2:636, 4:176, 6:5 |
| No.7-C1.cc1 | C86 | 0.218 | 1.0 | 2 | 0:219, 2:630, 4:142, 6:9 |
| No.8-Cs.cc1 | C86 | 0.157 | 1.0 | 3 | 0:212, 2:649, 4:136, 6:3 |
| No.9-C2v.cc1 | C86 | 0.125 | 1.1 | 4 | 0:208, 2:629, 4:156, 6:7 |
| No.1-D5h.cc1 | C90 | 0.110 | 1.0 | 3 | 0:162, 2:616, 4:205, 6:17 |
| No.10-Cs.cc1 | C90 | 0.157 | 1.0 | 2 | 0:169, 2:661, 4:162, 6:8 |
| No.11-C1.cc1 | C90 | 0.250 | 1.0 | 2 | 0:193, 2:636, 4:164, 6:7 |
| No.12-C2.cc1 | C90 | 0.203 | 1.0 | 2 | 0:159, 2:658, 4:180, 6:3 |
| No.13-C2v.cc1 | C90 | 0.141 | 1.0 | 2 | 0:179, 2:627, 4:185, 6:9 |
| No.14-C1.cc1 | C90 | 0.125 | 1.0 | 2 | 0:162, 2:657, 4:172, 6:9 |
| No.15-C1.cc1 | C90 | 0.156 | 1.0 | 2 | 0:148, 2:666, 4:178, 6:8 |
| No.16-C2v.cc1 | C90 | 0.219 | 1.0 | 3 | 0:167, 2:667, 4:158, 6:8 |
| No.17-Cs.cc1 | C90 | 0.250 | 1.0 | 2 | 0:160, 2:658, 4:173, 6:9 |
| No.18-C2.cc1 | C90 | 0.234 | 1.0 | 3 | 0:200, 2:621, 4:168, 6:11 |
| No.19-C2.cc1 | C90 | 0.156 | 1.0 | 3 | 0:186, 2:631, 4:176, 6:5, 8:2 |
| No.2-C2v.cc1 | C90 | 0.125 | 1.0 | 2 | 0:152, 2:634, 4:205, 6:9 |
| No.20-C1.cc1 | C90 | 0.110 | 1.0 | 3 | 0:185, 2:637, 4:172, 6:6 |
| No.21-C1.cc1 | C90 | 0.109 | 1.1 | 3 | 0:201, 2:628, 4:163, 6:8 |
| No.22-C1.cc1 | C90 | 0.172 | 1.0 | 3 | 0:191, 2:642, 4:159, 6:8 |
| No.23-C2.cc1 | C90 | 0.250 | 1.0 | 3 | 0:179, 2:646, 4:169, 6:6 |
| No.24-C1.cc1 | C90 | 0.156 | 1.0 | 3 | 0:163, 2:644, 4:186, 6:6, 8:1 |
| No.25-C2v.cc1 | C90 | 0.125 | 1.0 | 2 | 0:159, 2:648, 4:186, 6:7 |
| No.26-C1.cc1 | C90 | 0.109 | 1.0 | 2 | 0:188, 2:635, 4:171, 6:6 |
| No.27-C1.cc1 | C90 | 0.094 | 1.0 | 3 | 0:191, 2:644, 4:158, 6:7 |
| No.28-C2.cc1 | C90 | 0.093 | 1.0 | 2 | 0:173, 2:694, 4:127, 6:6 |
| No.29-C1.cc1 | C90 | 0.094 | 1.0 | 3 | 0:192, 2:647, 4:146, 6:15 |
| No.3-C1.cc1 | C90 | 0.094 | 1.0 | 2 | 0:167, 2:635, 4:189, 6:8, 8:1 |
| No.30-C1.cc1 | C90 | 0.078 | 1.0 | 3 | 0:192, 2:656, 4:144, 6:8 |
| No.31-C2.cc1 | C90 | 0.093 | 1.0 | 3 | 0:193, 2:632, 4:166, 6:9 |
| No.32-C1.cc1 | C90 | 0.250 | 1.0 | 2 | 0:211, 2:623, 4:154, 6:11, 8:1 |
| No.33-Cs.cc1 | C90 | 0.250 | 1.0 | 2 | 0:222, 2:613, 4:158, 6:7 |
| No.34-Cs.cc1 | C90 | 0.156 | 1.0 | 2 | 0:173, 2:652, 4:165, 6:10 |
| No.35-Cs.cc1 | C90 | 0.125 | 1.0 | 3 | 0:190, 2:642, 4:160, 6:8 |
| No.36-C2v.cc1 | C90 | 0.172 | 1.0 | 3 | 0:163, 2:670, 4:159, 6:8 |
| No.37-C2.cc1 | C90 | 0.250 | 1.0 | 3 | 0:196, 2:616, 4:181, 6:7 |
| No.38-C1.cc1 | C90 | 0.157 | 1.0 | 3 | 0:197, 2:627, 4:175, 6:1 |
| No.39-C2v.cc1 | C90 | 0.203 | 1.0 | 2 | 0:182, 2:629, 4:184, 6:5 |
| No.4-C2.cc1 | C90 | 0.235 | 1.0 | 2 | 0:151, 2:658, 4:176, 6:15 |
| No.40-C2.cc1 | C90 | 0.141 | 1.0 | 2 | 0:203, 2:659, 4:130, 6:8 |
| No.41-C2.cc1 | C90 | 0.125 | 1.0 | 3 | 0:186, 2:659, 4:148, 6:7 |
| No.42-C2.cc1 | C90 | 0.109 | 1.1 | 2 | 0:185, 2:658, 4:149, 6:8 |
| No.43-C2.cc1 | C90 | 0.094 | 1.1 | 3 | 0:203, 2:631, 4:158, 6:8 |
| No.44-C2.cc1 | C90 | 0.093 | 1.0 | 3 | 0:197, 2:659, 4:136, 6:8 |
| No.45-C2.cc1 | C90 | 0.094 | 1.0 | 3 | 0:200, 2:646, 4:152, 6:2 |
| No.46-C2v.cc1 | C90 | 0.203 | 1.0 | 3 | 0:200, 2:629, 4:165, 6:5, 8:1 |
| No.5-Cs.cc1 | C90 | 0.187 | 1.0 | 2 | 0:181, 2:623, 4:190, 6:6 |
| No.6-C2.cc1 | C90 | 0.141 | 1.0 | 3 | 0:165, 2:658, 4:169, 6:8 |
| No.7-C1.cc1 | C90 | 0.109 | 1.0 | 3 | 0:191, 2:621, 4:180, 6:7, 8:1 |
| No.8-C2.cc1 | C90 | 0.094 | 1.0 | 2 | 0:168, 2:642, 4:180, 6:10 |
| No.9-C1.cc1 | C90 | 0.094 | 1.0 | 3 | 0:186, 2:666, 4:142, 6:6 |
| No.1-D2.cc1 | C92 | 0.093 | 1.0 | 2 | 0:160, 2:602, 4:220, 6:17, 8:1 |
| No.10-C1.cc1 | C92 | 0.094 | 1.0 | 3 | 0:185, 2:638, 4:169, 6:8 |
| No.11-C1.cc1 | C92 | 0.094 | 1.0 | 2 | 0:186, 2:630, 4:179, 6:5 |
| No.12-C1.cc1 | C92 | 0.079 | 1.0 | 2 | 0:172, 2:654, 4:167, 6:7 |
| No.13-C1.cc1 | C92 | 0.093 | 1.0 | 2 | 0:185, 2:661, 4:143, 6:10, 8:1 |
| No.14-Cs.cc1 | C92 | 0.094 | 1.0 | 2 | 0:167, 2:637, 4:187, 6:9 |
| No.15-Cs.cc1 | C92 | 0.141 | 1.0 | 2 | 0:178, 2:641, 4:175, 6:6 |
| No.16-Cs.cc1 | C92 | 0.172 | 1.1 | 3 | 0:168, 2:654, 4:166, 6:12 |
| No.17-C2.cc1 | C92 | 0.203 | 1.0 | 3 | 0:163, 2:618, 4:205, 6:14 |
| No.18-C1.cc1 | C92 | 0.141 | 1.0 | 3 | 0:181, 2:647, 4:165, 6:6, 8:1 |
| No.19-C2.cc1 | C92 | 0.110 | 1.0 | 3 | 0:169, 2:620, 4:199, 6:11, 8:1 |
| No.2-C1.cc1 | C92 | 0.094 | 1.0 | 2 | 0:163, 2:662, 4:168, 6:6, 8:1 |
| No.20-C1.cc1 | C92 | 0.094 | 1.0 | 2 | 0:174, 2:617, 4:200, 6:9 |
| No.21-Cs.cc1 | C92 | 0.094 | 1.0 | 2 | 0:163, 2:620, 4:194, 6:23 |
| No.22-C2v.cc1 | C92 | 0.094 | 1.0 | 3 | 0:151, 2:610, 4:226, 6:13 |
| No.23-C2.cc1 | C92 | 0.094 | 1.0 | 3 | 0:160, 2:655, 4:176, 6:9 |
| No.24-Cs.cc1 | C92 | 0.093 | 1.0 | 3 | 0:189, 2:602, 4:201, 6:8 |
| No.25-C2.cc1 | C92 | 0.094 | 1.0 | 3 | 0:167, 2:644, 4:176, 6:13 |
| No.26-C2.cc1 | C92 | 0.172 | 1.0 | 2 | 0:193, 2:629, 4:168, 6:8, 8:2 |
| No.27-C2.cc1 | C92 | 0.203 | 1.0 | 3 | 0:184, 2:619, 4:187, 6:10 |
| No.28-D3.cc1 | C92 | 0.234 | 1.0 | 3 | 0:206, 2:605, 4:174, 6:15 |
| No.29-D2h.cc1 | C92 | 0.156 | 1.0 | 2 | 0:153, 2:626, 4:205, 6:16 |
| No.3-C2.cc1 | C92 | 0.140 | 1.0 | 2 | 0:138, 2:620, 4:229, 6:13 |
| No.30-C1.cc1 | C92 | 0.109 | 1.0 | 3 | 0:203, 2:615, 4:171, 6:11 |
| No.31-C2.cc1 | C92 | 0.094 | 1.0 | 4 | 0:188, 2:633, 4:171, 6:7, 8:1 |
| No.32-C1.cc1 | C92 | 0.094 | 1.0 | 4 | 0:185, 2:630, 4:179, 6:6 |
| No.33-C1.cc1 | C92 | 0.094 | 1.0 | 3 | 0:190, 2:639, 4:165, 6:5, 8:1 |
| No.34-C2.cc1 | C92 | 0.110 | 1.0 | 2 | 0:177, 2:652, 4:164, 6:7 |
| No.35-C2v.cc1 | C92 | 0.109 | 1.0 | 3 | 0:180, 2:621, 4:185, 6:13, 8:1 |
| No.36-C2.cc1 | C92 | 0.219 | 1.0 | 3 | 0:176, 2:633, 4:182, 6:8, 8:1 |
| No.37-C1.cc1 | C92 | 0.172 | 1.0 | 3 | 0:175, 2:631, 4:186, 6:8 |
| No.38-C1.cc1 | C92 | 0.125 | 1.0 | 3 | 0:188, 2:636, 4:167, 6:9 |
| No.39-C1.cc1 | C92 | 0.109 | 1.0 | 3 | 0:186, 2:645, 4:162, 6:7 |
| No.4-C2.cc1 | C92 | 0.110 | 1.0 | 3 | 0:163, 2:639, 4:188, 6:10 |
| No.40-C1.cc1 | C92 | 0.203 | 1.0 | 3 | 0:163, 2:657, 4:174, 6:6 |
| No.41-C3.cc1 | C92 | 0.250 | 1.0 | 3 | 0:211, 2:619, 4:165, 6:5 |
| No.42-C1.cc1 | C92 | 0.235 | 1.0 | 3 | 0:191, 2:643, 4:164, 6:2 |
| No.43-C1.cc1 | C92 | 0.204 | 1.0 | 2 | 0:185, 2:648, 4:156, 6:11 |
| No.44-C1.cc1 | C92 | 0.141 | 1.0 | 4 | 0:157, 2:675, 4:160, 6:8 |
| No.45-Cs.cc1 | C92 | 0.125 | 1.0 | 2 | 0:183, 2:640, 4:167, 6:10 |
| No.46-C2.cc1 | C92 | 0.094 | 1.0 | 2 | 0:167, 2:662, 4:164, 6:7 |
| No.47-C1.cc1 | C92 | 0.141 | 1.0 | 3 | 0:186, 2:628, 4:176, 6:10 |
| No.48-C2.cc1 | C92 | 0.203 | 1.0 | 3 | 0:179, 2:630, 4:180, 6:11 |
| No.49-C2.cc1 | C92 | 0.156 | 1.0 | 3 | 0:174, 2:647, 4:173, 6:6 |
| No.5-Cs.cc1 | C92 | 0.125 | 1.0 | 2 | 0:173, 2:631, 4:182, 6:14 |
| No.50-C1.cc1 | C92 | 0.110 | 1.0 | 3 | 0:190, 2:636, 4:166, 6:8 |
| No.51-C2.cc1 | C92 | 0.188 | 1.0 | 2 | 0:160, 2:655, 4:179, 6:6 |
| No.52-C1.cc1 | C92 | 0.203 | 1.0 | 4 | 0:181, 2:625, 4:185, 6:9 |
| No.53-C1.cc1 | C92 | 0.141 | 1.0 | 3 | 0:209, 2:606, 4:177, 6:7, 8:1 |
| No.54-C1.cc1 | C92 | 0.235 | 1.0 | 4 | 0:179, 2:643, 4:165, 6:13 |
| No.55-C1.cc1 | C92 | 0.235 | 1.0 | 2 | 0:189, 2:632, 4:172, 6:7 |
| No.56-C1.cc1 | C92 | 0.203 | 1.0 | 3 | 0:174, 2:629, 4:184, 6:13 |
| No.57-C1.cc1 | C92 | 0.219 | 1.0 | 3 | 0:159, 2:677, 4:159, 6:5 |
| No.58-C1.cc1 | C92 | 0.235 | 1.0 | 3 | 0:183, 2:642, 4:165, 6:10 |
| No.59-C1.cc1 | C92 | 0.157 | 1.0 | 3 | 0:154, 2:677, 4:158, 6:11 |
| No.6-Cs.cc1 | C92 | 0.125 | 1.0 | 3 | 0:156, 2:652, 4:186, 6:6 |
| No.60-C1.cc1 | C92 | 0.125 | 1.0 | 3 | 0:194, 2:635, 4:166, 6:5 |
| No.61-C2.cc1 | C92 | 0.109 | 1.0 | 3 | 0:181, 2:677, 4:133, 6:9 |
| No.62-C1.cc1 | C92 | 0.094 | 1.0 | 2 | 0:211, 2:640, 4:141, 6:8 |
| No.63-C1.cc1 | C92 | 0.093 | 1.0 | 3 | 0:184, 2:647, 4:166, 6:3 |
| No.64-C2.cc1 | C92 | 0.078 | 1.1 | 3 | 0:199, 2:623, 4:174, 6:4 |
| No.65-C2.cc1 | C92 | 0.109 | 1.0 | 3 | 0:168, 2:666, 4:163, 6:3 |
| No.66-C1.cc1 | C92 | 0.110 | 1.1 | 3 | 0:181, 2:654, 4:158, 6:7 |
| No.67-C1.cc1 | C92 | 0.218 | 1.0 | 3 | 0:203, 2:624, 4:167, 6:6 |
| No.68-C1.cc1 | C92 | 0.234 | 1.0 | 2 | 0:175, 2:678, 4:138, 6:8, 8:1 |
| No.69-C2.cc1 | C92 | 0.203 | 1.0 | 2 | 0:190, 2:655, 4:147, 6:8 |
| No.7-C2.cc1 | C92 | 0.141 | 1.0 | 2 | 0:178, 2:651, 4:157, 6:11, 8:3 |
| No.70-C1.cc1 | C92 | 0.125 | 1.0 | 3 | 0:191, 2:627, 4:172, 6:10 |
| No.71-D3.cc1 | C92 | 0.093 | 1.0 | 2 | 0:193, 2:658, 4:145, 6:4 |
| No.72-C1.cc1 | C92 | 0.094 | 1.0 | 2 | 0:176, 2:654, 4:161, 6:8, 8:1 |
| No.73-C1.cc1 | C92 | 0.094 | 1.0 | 2 | 0:187, 2:621, 4:188, 6:4 |
| No.74-C2.cc1 | C92 | 0.094 | 1.0 | 3 | 0:166, 2:668, 4:163, 6:3 |
| No.75-C2.cc1 | C92 | 0.187 | 1.0 | 3 | 0:165, 2:666, 4:162, 6:7 |
| No.76-C1.cc1 | C92 | 0.266 | 1.0 | 3 | 0:168, 2:642, 4:181, 6:9 |
| No.77-C2.cc1 | C92 | 0.250 | 1.0 | 3 | 0:192, 2:635, 4:169, 6:4 |
| No.78-D3.cc1 | C92 | 0.234 | 1.0 | 2 | 0:192, 2:641, 4:159, 6:8 |
| No.79-C2.cc1 | C92 | 0.141 | 1.0 | 3 | 0:177, 2:664, 4:155, 6:4 |
| No.8-C1.cc1 | C92 | 0.125 | 1.0 | 3 | 0:176, 2:635, 4:178, 6:11 |
| No.80-C2.cc1 | C92 | 0.110 | 1.0 | 2 | 0:163, 2:667, 4:159, 6:10, 8:1 |
| No.81-D2.cc1 | C92 | 0.093 | 1.0 | 3 | 0:187, 2:624, 4:176, 6:13 |
| No.82-D2.cc1 | C92 | 0.125 | 1.0 | 3 | 0:194, 2:639, 4:159, 6:8 |
| No.83-D3.cc1 | C92 | 0.109 | 1.0 | 3 | 0:171, 2:671, 4:153, 6:5 |
| No.84-D2.cc1 | C92 | 0.110 | 1.0 | 3 | 0:194, 2:642, 4:158, 6:6 |
| No.85-D3.cc1 | C92 | 0.094 | 1.0 | 3 | 0:187, 2:654, 4:155, 6:4 |
| No.86-T.cc1 | C92 | 0.172 | 1.0 | 3 | 0:200, 2:640, 4:153, 6:7 |
| No.9-C2.cc1 | C92 | 0.250 | 1.0 | 2 | 0:181, 2:637, 4:175, 6:6, 8:1 |
| No.1-C2.cc1 | C94 | 0.250 | 1.0 | 3 | 0:149, 2:639, 4:197, 6:15 |
| No.10-C1.cc1 | C94 | 0.188 | 1.0 | 4 | 0:189, 2:618, 4:189, 6:4 |
| No.100-C1.cc1 | C94 | 0.204 | 1.0 | 2 | 0:163, 2:641, 4:186, 6:10 |
| No.101-C1.cc1 | C94 | 0.203 | 1.0 | 2 | 0:182, 2:640, 4:169, 6:9 |
| No.102-C1.cc1 | C94 | 0.157 | 1.0 | 3 | 0:157, 2:645, 4:190, 6:8 |
| No.103-C1.cc1 | C94 | 0.125 | 1.0 | 2 | 0:182, 2:649, 4:161, 6:7, 8:1 |
| No.104-C1.cc1 | C94 | 0.109 | 1.0 | 3 | 0:160, 2:670, 4:161, 6:9 |
| No.105-C1.cc1 | C94 | 0.234 | 1.0 | 2 | 0:179, 2:643, 4:169, 6:9 |
| No.106-C1.cc1 | C94 | 0.250 | 1.0 | 2 | 0:152, 2:647, 4:190, 6:11 |
| No.107-C2.cc1 | C94 | 0.250 | 1.0 | 2 | 0:187, 2:621, 4:182, 6:9, 8:1 |
| No.108-C2.cc1 | C94 | 0.265 | 1.0 | 2 | 0:166, 2:634, 4:189, 6:11 |
| No.109-C2.cc1 | C94 | 0.188 | 1.0 | 2 | 0:177, 2:619, 4:196, 6:8 |
| No.11-C1.cc1 | C94 | 0.235 | 1.0 | 3 | 0:178, 2:619, 4:189, 6:13, 8:1 |
| No.110-C3.cc1 | C94 | 0.156 | 1.0 | 2 | 0:173, 2:631, 4:187, 6:9 |
| No.111-C1.cc1 | C94 | 0.157 | 1.0 | 3 | 0:185, 2:622, 4:189, 6:4 |
| No.112-C1.cc1 | C94 | 0.234 | 1.0 | 3 | 0:191, 2:625, 4:177, 6:7 |
| No.113-C1.cc1 | C94 | 0.235 | 1.0 | 3 | 0:168, 2:629, 4:197, 6:6 |
| No.114-C3.cc1 | C94 | 0.234 | 1.0 | 3 | 0:164, 2:662, 4:165, 6:9 |
| No.115-C1.cc1 | C94 | 0.203 | 1.0 | 2 | 0:181, 2:659, 4:154, 6:6 |
| No.116-C1.cc1 | C94 | 0.157 | 1.1 | 3 | 0:187, 2:636, 4:171, 6:6 |
| No.117-C2.cc1 | C94 | 0.218 | 1.1 | 3 | 0:177, 2:645, 4:170, 6:8 |
| No.118-C1.cc1 | C94 | 0.188 | 1.1 | 4 | 0:205, 2:629, 4:158, 6:8 |
| No.119-C1.cc1 | C94 | 0.266 | 1.1 | 3 | 0:191, 2:653, 4:150, 6:6 |
| No.12-C1.cc1 | C94 | 0.219 | 1.0 | 2 | 0:172, 2:642, 4:178, 6:8 |
| No.120-Cs.cc1 | C94 | 0.156 | 1.1 | 3 | 0:183, 2:656, 4:151, 6:10 |
| No.121-C2.cc1 | C94 | 0.125 | 1.0 | 3 | 0:184, 2:636, 4:169, 6:11 |
| No.122-C1.cc1 | C94 | 0.110 | 1.0 | 2 | 0:191, 2:639, 4:165, 6:5 |
| No.123-C1.cc1 | C94 | 0.125 | 1.0 | 3 | 0:163, 2:663, 4:160, 6:14 |
| No.124-C2.cc1 | C94 | 0.110 | 1.0 | 3 | 0:173, 2:670, 4:150, 6:7 |
| No.125-C1.cc1 | C94 | 0.094 | 1.0 | 3 | 0:162, 2:671, 4:159, 6:7, 8:1 |
| No.126-C2.cc1 | C94 | 0.094 | 1.0 | 2 | 0:186, 2:669, 4:134, 6:11 |
| No.127-C1.cc1 | C94 | 0.094 | 1.1 | 3 | 0:178, 2:663, 4:149, 6:10 |
| No.128-C1.cc1 | C94 | 0.093 | 1.0 | 2 | 0:185, 2:649, 4:160, 6:6 |
| No.129-C1.cc1 | C94 | 0.172 | 1.0 | 3 | 0:167, 2:680, 4:143, 6:10 |
| No.13-Cs.cc1 | C94 | 0.250 | 1.0 | 2 | 0:190, 2:614, 4:182, 6:14 |
| No.130-C2.cc1 | C94 | 0.250 | 1.0 | 3 | 0:197, 2:639, 4:161, 6:3 |
| No.131-C1.cc1 | C94 | 0.156 | 1.0 | 3 | 0:169, 2:671, 4:152, 6:8 |
| No.132-C1.cc1 | C94 | 0.125 | 1.0 | 3 | 0:193, 2:632, 4:163, 6:12 |
| No.133-C2.cc1 | C94 | 0.110 | 1.0 | 3 | 0:188, 2:634, 4:168, 6:9, 8:1 |
| No.134-C3v.cc1 | C94 | 0.109 | 1.0 | 3 | 0:178, 2:658, 4:157, 6:7 |
| No.14-Cs.cc1 | C94 | 0.171 | 1.0 | 3 | 0:164, 2:637, 4:182, 6:17 |
| No.15-C1.cc1 | C94 | 0.172 | 1.0 | 2 | 0:151, 2:675, 4:167, 6:6, 8:1 |
| No.16-C1.cc1 | C94 | 0.234 | 1.0 | 3 | 0:209, 2:629, 4:150, 6:12 |
| No.17-Cs.cc1 | C94 | 0.203 | 1.0 | 2 | 0:187, 2:641, 4:157, 6:15 |
| No.18-Cs.cc1 | C94 | 0.156 | 1.0 | 2 | 0:195, 2:625, 4:173, 6:7 |
| No.19-C1.cc1 | C94 | 0.187 | 1.0 | 3 | 0:172, 2:630, 4:189, 6:9 |
| No.2-C1.cc1 | C94 | 0.141 | 1.0 | 2 | 0:161, 2:637, 4:183, 6:19 |
| No.20-C2.cc1 | C94 | 0.125 | 1.0 | 3 | 0:163, 2:640, 4:191, 6:6 |
| No.21-C1.cc1 | C94 | 0.094 | 1.0 | 4 | 0:176, 2:628, 4:189, 6:7 |
| No.22-C2.cc1 | C94 | 0.110 | 1.0 | 2 | 0:160, 2:651, 4:179, 6:10 |
| No.23-Cs.cc1 | C94 | 0.094 | 1.0 | 3 | 0:182, 2:620, 4:193, 6:5 |
| No.24-C2v.cc1 | C94 | 0.093 | 1.0 | 2 | 0:164, 2:643, 4:186, 6:7 |
| No.25-C1.cc1 | C94 | 0.094 | 1.0 | 3 | 0:156, 2:659, 4:180, 6:5 |
| No.26-C2.cc1 | C94 | 0.094 | 1.0 | 2 | 0:178, 2:617, 4:193, 6:12 |
| No.27-C1.cc1 | C94 | 0.109 | 1.0 | 3 | 0:151, 2:649, 4:192, 6:8 |
| No.28-C1.cc1 | C94 | 0.093 | 1.0 | 3 | 0:183, 2:608, 4:205, 6:4 |
| No.29-C1.cc1 | C94 | 0.125 | 1.0 | 2 | 0:181, 2:624, 4:186, 6:8, 8:1 |
| No.3-C2.cc1 | C94 | 0.110 | 1.0 | 2 | 0:148, 2:616, 4:227, 6:9 |
| No.30-C1.cc1 | C94 | 0.093 | 1.0 | 3 | 0:173, 2:610, 4:197, 6:20 |
| No.31-Cs.cc1 | C94 | 0.203 | 1.0 | 3 | 0:180, 2:641, 4:172, 6:7 |
| No.32-C1.cc1 | C94 | 0.187 | 1.0 | 3 | 0:154, 2:634, 4:198, 6:13, 8:1 |
| No.33-C2.cc1 | C94 | 0.141 | 1.0 | 3 | 0:188, 2:646, 4:161, 6:5 |
| No.34-C1.cc1 | C94 | 0.125 | 1.0 | 3 | 0:190, 2:637, 4:165, 6:8 |
| No.35-C1.cc1 | C94 | 0.234 | 1.0 | 2 | 0:189, 2:634, 4:172, 6:5 |
| No.36-C1.cc1 | C94 | 0.250 | 1.0 | 3 | 0:182, 2:630, 4:178, 6:10 |
| No.37-C1.cc1 | C94 | 0.156 | 1.0 | 2 | 0:175, 2:641, 4:171, 6:13 |
| No.38-C1.cc1 | C94 | 0.235 | 1.0 | 3 | 0:152, 2:632, 4:195, 6:20, 8:1 |
| No.39-Cs.cc1 | C94 | 0.250 | 1.0 | 3 | 0:192, 2:629, 4:171, 6:8 |
| No.4-C2.cc1 | C94 | 0.203 | 1.0 | 3 | 0:144, 2:607, 4:236, 6:13 |
| No.40-Cs.cc1 | C94 | 0.140 | 1.0 | 2 | 0:151, 2:652, 4:179, 6:17, 8:1 |
| No.41-C1.cc1 | C94 | 0.219 | 1.0 | 2 | 0:184, 2:608, 4:193, 6:14, 8:1 |
| No.42-Cs.cc1 | C94 | 0.250 | 1.0 | 2 | 0:199, 2:620, 4:174, 6:7 |
| No.43-C2.cc1 | C94 | 0.250 | 1.0 | 2 | 0:192, 2:648, 4:157, 6:3 |
| No.44-Cs.cc1 | C94 | 0.203 | 1.0 | 2 | 0:197, 2:632, 4:165, 6:6 |
| No.45-Cs.cc1 | C94 | 0.203 | 1.0 | 2 | 0:148, 2:659, 4:185, 6:8 |
| No.46-Cs.cc1 | C94 | 0.234 | 1.0 | 4 | 0:162, 2:622, 4:194, 6:22 |
| No.47-C2.cc1 | C94 | 0.250 | 1.0 | 2 | 0:169, 2:653, 4:169, 6:9 |
| No.48-C1.cc1 | C94 | 0.219 | 1.0 | 3 | 0:177, 2:626, 4:187, 6:10 |
| No.49-C1.cc1 | C94 | 0.156 | 1.0 | 3 | 0:176, 2:649, 4:163, 6:12 |
| No.5-C1.cc1 | C94 | 0.203 | 1.0 | 2 | 0:147, 2:622, 4:219, 6:12 |
| No.50-C1.cc1 | C94 | 0.219 | 1.0 | 3 | 0:169, 2:633, 4:192, 6:5, 8:1 |
| No.51-C2.cc1 | C94 | 0.250 | 1.0 | 2 | 0:177, 2:642, 4:168, 6:13 |
| No.52-C2v.cc1 | C94 | 0.203 | 1.0 | 2 | 0:158, 2:645, 4:187, 6:10 |
| No.53-C1.cc1 | C94 | 0.250 | 1.0 | 3 | 0:170, 2:631, 4:186, 6:13 |
| No.54-C1.cc1 | C94 | 0.156 | 1.0 | 3 | 0:160, 2:667, 4:163, 6:10 |
| No.55-C1.cc1 | C94 | 0.141 | 1.0 | 3 | 0:180, 2:639, 4:173, 6:8 |
| No.56-C1.cc1 | C94 | 0.109 | 1.0 | 2 | 0:162, 2:656, 4:170, 6:12 |
| No.57-C1.cc1 | C94 | 0.093 | 1.0 | 3 | 0:165, 2:658, 4:165, 6:12 |
| No.58-C1.cc1 | C94 | 0.094 | 1.0 | 3 | 0:145, 2:643, 4:205, 6:7 |
| No.59-C1.cc1 | C94 | 0.094 | 1.0 | 3 | 0:182, 2:649, 4:163, 6:6 |
| No.6-C1.cc1 | C94 | 0.094 | 1.0 | 3 | 0:180, 2:596, 4:211, 6:13 |
| No.60-C1.cc1 | C94 | 0.125 | 1.0 | 3 | 0:168, 2:645, 4:180, 6:7 |
| No.61-C2.cc1 | C94 | 0.109 | 1.0 | 2 | 0:192, 2:627, 4:169, 6:12 |
| No.62-C1.cc1 | C94 | 0.110 | 1.0 | 3 | 0:199, 2:619, 4:173, 6:9 |
| No.63-C1.cc1 | C94 | 0.156 | 1.0 | 3 | 0:177, 2:636, 4:176, 6:11 |
| No.64-C1.cc1 | C94 | 0.297 | 1.0 | 2 | 0:139, 2:642, 4:206, 6:13 |
| No.65-C1.cc1 | C94 | 0.203 | 1.0 | 2 | 0:169, 2:676, 4:149, 6:6 |
| No.66-C1.cc1 | C94 | 0.234 | 1.0 | 4 | 0:187, 2:651, 4:155, 6:7 |
| No.67-C1.cc1 | C94 | 0.203 | 1.0 | 3 | 0:157, 2:645, 4:188, 6:10 |
| No.68-C1.cc1 | C94 | 0.141 | 1.0 | 3 | 0:190, 2:640, 4:161, 6:9 |
| No.69-C1.cc1 | C94 | 0.172 | 1.0 | 3 | 0:178, 2:640, 4:179, 6:3 |
| No.7-C2.cc1 | C94 | 0.203 | 1.0 | 3 | 0:174, 2:632, 4:182, 6:12 |
| No.70-C1.cc1 | C94 | 0.234 | 1.0 | 3 | 0:180, 2:637, 4:173, 6:9, 8:1 |
| No.71-C1.cc1 | C94 | 0.156 | 1.0 | 3 | 0:153, 2:658, 4:177, 6:12 |
| No.72-C1.cc1 | C94 | 0.141 | 1.0 | 3 | 0:194, 2:629, 4:171, 6:6 |
| No.73-C2.cc1 | C94 | 0.109 | 1.0 | 3 | 0:155, 2:646, 4:194, 6:5 |
| No.74-C1.cc1 | C94 | 0.094 | 1.0 | 3 | 0:172, 2:637, 4:181, 6:10 |
| No.75-C1.cc1 | C94 | 0.093 | 1.0 | 2 | 0:175, 2:637, 4:184, 6:4 |
| No.76-C1.cc1 | C94 | 0.141 | 1.0 | 3 | 0:158, 2:658, 4:172, 6:12 |
| No.77-C1.cc1 | C94 | 0.219 | 1.0 | 2 | 0:157, 2:672, 4:164, 6:7 |
| No.78-C1.cc1 | C94 | 0.250 | 1.0 | 2 | 0:195, 2:631, 4:166, 6:7, 8:1 |
| No.79-C1.cc1 | C94 | 0.250 | 1.0 | 3 | 0:162, 2:660, 4:168, 6:10 |
| No.8-C1.cc1 | C94 | 0.235 | 1.0 | 3 | 0:171, 2:620, 4:201, 6:8 |
| No.80-C1.cc1 | C94 | 0.157 | 1.0 | 3 | 0:171, 2:657, 4:162, 6:10 |
| No.81-C1.cc1 | C94 | 0.156 | 1.0 | 2 | 0:177, 2:637, 4:174, 6:12 |
| No.82-C1.cc1 | C94 | 0.140 | 1.0 | 4 | 0:175, 2:645, 4:170, 6:10 |
| No.83-C1.cc1 | C94 | 0.109 | 1.0 | 3 | 0:192, 2:627, 4:174, 6:7 |
| No.84-C1.cc1 | C94 | 0.110 | 1.0 | 3 | 0:182, 2:632, 4:174, 6:12 |
| No.85-C1.cc1 | C94 | 0.093 | 1.0 | 3 | 0:165, 2:659, 4:169, 6:7 |
| No.86-C2.cc1 | C94 | 0.094 | 1.0 | 2 | 0:155, 2:637, 4:196, 6:11, 8:1 |
| No.87-C1.cc1 | C94 | 0.172 | 1.0 | 2 | 0:166, 2:664, 4:165, 6:5 |
| No.88-C1.cc1 | C94 | 0.250 | 1.0 | 3 | 0:170, 2:639, 4:180, 6:11 |
| No.89-C2.cc1 | C94 | 0.235 | 1.0 | 2 | 0:152, 2:659, 4:181, 6:8 |
| No.9-C2.cc1 | C94 | 0.250 | 1.0 | 2 | 0:144, 2:644, 4:194, 6:18 |
| No.90-C1.cc1 | C94 | 0.156 | 1.0 | 2 | 0:182, 2:647, 4:165, 6:6 |
| No.91-C1.cc1 | C94 | 0.125 | 1.0 | 3 | 0:167, 2:656, 4:170, 6:7 |
| No.92-C1.cc1 | C94 | 0.109 | 1.0 | 3 | 0:200, 2:638, 4:154, 6:8 |
| No.93-C1.cc1 | C94 | 0.219 | 1.0 | 2 | 0:159, 2:663, 4:170, 6:8 |
| No.94-C2.cc1 | C94 | 0.250 | 1.0 | 2 | 0:169, 2:657, 4:165, 6:9 |
| No.95-C1.cc1 | C94 | 0.157 | 1.0 | 2 | 0:201, 2:625, 4:169, 6:5 |
| No.96-C3.cc1 | C94 | 0.125 | 1.0 | 3 | 0:180, 2:647, 4:166, 6:7 |
| No.97-C1.cc1 | C94 | 0.110 | 1.0 | 2 | 0:184, 2:646, 4:159, 6:11 |
| No.98-C1.cc1 | C94 | 0.094 | 1.0 | 3 | 0:175, 2:634, 4:182, 6:9 |
| No.99-C1.cc1 | C94 | 0.094 | 1.0 | 3 | 0:189, 2:633, 4:169, 6:9 |
| No.1-D2.cc1 | C96 | 0.109 | 1.0 | 2 | 0:144, 2:581, 4:250, 6:24, 8:1 |
| No.10-Cs.cc1 | C96 | 0.094 | 1.0 | 3 | 0:149, 2:592, 4:242, 6:17 |
| No.100-C1.cc1 | C96 | 0.109 | 1.0 | 2 | 0:138, 2:664, 4:193, 6:5 |
| No.101-C1.cc1 | C96 | 0.109 | 1.0 | 2 | 0:160, 2:656, 4:172, 6:12 |
| No.102-C1.cc1 | C96 | 0.188 | 1.0 | 3 | 0:171, 2:641, 4:181, 6:6, 8:1 |
| No.103-C1.cc1 | C96 | 0.250 | 1.0 | 2 | 0:172, 2:631, 4:185, 6:12 |
| No.104-C1.cc1 | C96 | 0.156 | 1.0 | 2 | 0:161, 2:630, 4:198, 6:11 |
| No.105-C2.cc1 | C96 | 0.172 | 1.0 | 2 | 0:151, 2:642, 4:200, 6:7 |
| No.106-C1.cc1 | C96 | 0.157 | 1.0 | 3 | 0:170, 2:643, 4:175, 6:11, 8:1 |
| No.107-C1.cc1 | C96 | 0.141 | 1.0 | 3 | 0:160, 2:670, 4:164, 6:6 |
| No.108-C1.cc1 | C96 | 0.125 | 1.0 | 3 | 0:167, 2:639, 4:178, 6:16 |
| No.109-D2.cc1 | C96 | 0.094 | 1.0 | 2 | 0:152, 2:643, 4:191, 6:13, 8:1 |
| No.11-Cs.cc1 | C96 | 0.094 | 1.0 | 2 | 0:178, 2:601, 4:204, 6:17 |
| No.110-C2v.cc1 | C96 | 0.140 | 1.0 | 3 | 0:161, 2:600, 4:226, 6:13 |
| No.111-D2.cc1 | C96 | 0.250 | 1.0 | 3 | 0:123, 2:632, 4:229, 6:16 |
| No.112-C1.cc1 | C96 | 0.172 | 1.0 | 3 | 0:176, 2:618, 4:195, 6:11 |
| No.113-C1.cc1 | C96 | 0.156 | 1.0 | 3 | 0:176, 2:641, 4:171, 6:12 |
| No.114-C1.cc1 | C96 | 0.218 | 1.0 | 2 | 0:197, 2:639, 4:156, 6:7, 8:1 |
| No.115-C2.cc1 | C96 | 0.266 | 1.0 | 3 | 0:184, 2:644, 4:168, 6:4 |
| No.116-C1.cc1 | C96 | 0.250 | 1.0 | 2 | 0:189, 2:632, 4:168, 6:11 |
| No.117-C1.cc1 | C96 | 0.203 | 1.0 | 2 | 0:165, 2:644, 4:182, 6:9 |
| No.118-C2.cc1 | C96 | 0.140 | 1.0 | 3 | 0:145, 2:662, 4:186, 6:7 |
| No.119-C1.cc1 | C96 | 0.172 | 1.0 | 3 | 0:174, 2:651, 4:166, 6:9 |
| No.12-C1.cc1 | C96 | 0.140 | 1.0 | 2 | 0:148, 2:655, 4:187, 6:10 |
| No.120-C1.cc1 | C96 | 0.109 | 1.0 | 2 | 0:155, 2:659, 4:176, 6:10 |
| No.121-D3.cc1 | C96 | 0.204 | 1.0 | 2 | 0:163, 2:643, 4:173, 6:21 |
| No.122-C2.cc1 | C96 | 0.188 | 1.0 | 2 | 0:176, 2:632, 4:185, 6:7 |
| No.123-C1.cc1 | C96 | 0.172 | 1.0 | 2 | 0:159, 2:651, 4:179, 6:11 |
| No.124-C2.cc1 | C96 | 0.125 | 1.0 | 3 | 0:160, 2:643, 4:188, 6:9 |
| No.125-C1.cc1 | C96 | 0.094 | 1.0 | 2 | 0:183, 2:642, 4:164, 6:11 |
| No.126-C2.cc1 | C96 | 0.093 | 1.0 | 3 | 0:175, 2:655, 4:159, 6:11 |
| No.127-C2.cc1 | C96 | 0.094 | 1.0 | 3 | 0:161, 2:663, 4:161, 6:15 |
| No.128-C1.cc1 | C96 | 0.203 | 1.0 | 3 | 0:169, 2:646, 4:176, 6:9 |
| No.129-C1.cc1 | C96 | 0.234 | 1.0 | 4 | 0:175, 2:629, 4:192, 6:4 |
| No.13-C2.cc1 | C96 | 0.172 | 1.0 | 2 | 0:141, 2:614, 4:231, 6:14 |
| No.130-C1.cc1 | C96 | 0.219 | 1.0 | 3 | 0:175, 2:641, 4:174, 6:10 |
| No.131-C1.cc1 | C96 | 0.203 | 1.0 | 3 | 0:155, 2:653, 4:185, 6:7 |
| No.132-C2.cc1 | C96 | 0.204 | 1.0 | 3 | 0:169, 2:655, 4:162, 6:14 |
| No.133-Cs.cc1 | C96 | 0.141 | 1.0 | 4 | 0:142, 2:651, 4:192, 6:15 |
| No.134-C1.cc1 | C96 | 0.188 | 1.0 | 3 | 0:154, 2:657, 4:182, 6:7 |
| No.135-C1.cc1 | C96 | 0.235 | 1.0 | 3 | 0:178, 2:629, 4:183, 6:10 |
| No.136-D3.cc1 | C96 | 0.250 | 1.0 | 2 | 0:165, 2:620, 4:205, 6:10 |
| No.137-C3v.cc1 | C96 | 0.250 | 1.0 | 2 | 0:190, 2:639, 4:162, 6:9 |
| No.138-C1.cc1 | C96 | 0.204 | 1.0 | 3 | 0:183, 2:624, 4:187, 6:6 |
| No.139-C1.cc1 | C96 | 0.172 | 1.0 | 2 | 0:176, 2:636, 4:177, 6:11 |
| No.14-C2.cc1 | C96 | 0.125 | 1.0 | 3 | 0:163, 2:633, 4:187, 6:17 |
| No.140-C1.cc1 | C96 | 0.157 | 1.0 | 3 | 0:176, 2:618, 4:198, 6:8 |
| No.141-C1.cc1 | C96 | 0.250 | 1.1 | 3 | 0:170, 2:636, 4:182, 6:12 |
| No.142-C2.cc1 | C96 | 0.156 | 1.0 | 3 | 0:158, 2:663, 4:173, 6:5, 8:1 |
| No.143-C1.cc1 | C96 | 0.203 | 1.0 | 3 | 0:166, 2:637, 4:186, 6:11 |
| No.144-C1.cc1 | C96 | 0.156 | 1.0 | 2 | 0:191, 2:622, 4:181, 6:5, 8:1 |
| No.145-C1.cc1 | C96 | 0.125 | 1.0 | 3 | 0:181, 2:622, 4:186, 6:11 |
| No.146-Cs.cc1 | C96 | 0.110 | 1.0 | 2 | 0:180, 2:655, 4:156, 6:9 |
| No.147-Cs.cc1 | C96 | 0.094 | 1.1 | 3 | 0:193, 2:629, 4:171, 6:7 |
| No.148-D2h.cc1 | C96 | 0.094 | 1.0 | 2 | 0:135, 2:631, 4:213, 6:21 |
| No.149-C2v.cc1 | C96 | 0.172 | 1.0 | 2 | 0:169, 2:622, 4:200, 6:9 |
| No.15-C2.cc1 | C96 | 0.235 | 1.0 | 2 | 0:154, 2:629, 4:202, 6:15 |
| No.150-D2.cc1 | C96 | 0.203 | 1.0 | 3 | 0:174, 2:609, 4:201, 6:16 |
| No.151-C2.cc1 | C96 | 0.141 | 1.0 | 2 | 0:152, 2:632, 4:207, 6:9 |
| No.152-C1.cc1 | C96 | 0.218 | 1.1 | 3 | 0:180, 2:635, 4:175, 6:10 |
| No.153-C1.cc1 | C96 | 0.172 | 1.0 | 3 | 0:166, 2:639, 4:189, 6:6 |
| No.154-D2.cc1 | C96 | 0.141 | 1.0 | 3 | 0:174, 2:629, 4:188, 6:9 |
| No.155-C1.cc1 | C96 | 0.172 | 1.0 | 3 | 0:178, 2:644, 4:172, 6:6 |
| No.156-Cs.cc1 | C96 | 0.250 | 1.0 | 3 | 0:158, 2:647, 4:187, 6:8 |
| No.157-C2.cc1 | C96 | 0.172 | 1.0 | 2 | 0:161, 2:647, 4:183, 6:9 |
| No.158-C2.cc1 | C96 | 0.125 | 1.0 | 3 | 0:173, 2:654, 4:160, 6:13 |
| No.159-C1.cc1 | C96 | 0.187 | 1.0 | 3 | 0:177, 2:623, 4:191, 6:9 |
| No.16-C1.cc1 | C96 | 0.188 | 1.0 | 2 | 0:157, 2:619, 4:214, 6:9, 8:1 |
| No.160-C1.cc1 | C96 | 0.203 | 1.1 | 4 | 0:171, 2:640, 4:175, 6:14 |
| No.161-Cs.cc1 | C96 | 0.141 | 1.0 | 3 | 0:188, 2:631, 4:172, 6:9 |
| No.162-C2v.cc1 | C96 | 0.218 | 1.0 | 3 | 0:187, 2:633, 4:168, 6:12 |
| No.163-D2d.cc1 | C96 | 0.250 | 1.0 | 2 | 0:176, 2:627, 4:185, 6:12 |
| No.164-C1.cc1 | C96 | 0.203 | 1.0 | 3 | 0:170, 2:660, 4:162, 6:8 |
| No.165-C2.cc1 | C96 | 0.156 | 1.0 | 3 | 0:181, 2:627, 4:182, 6:10 |
| No.166-C1.cc1 | C96 | 0.125 | 1.0 | 3 | 0:192, 2:604, 4:199, 6:5 |
| No.167-C2.cc1 | C96 | 0.109 | 1.0 | 2 | 0:186, 2:649, 4:153, 6:12 |
| No.168-C2.cc1 | C96 | 0.203 | 1.0 | 3 | 0:156, 2:647, 4:192, 6:5 |
| No.169-C1.cc1 | C96 | 0.250 | 1.0 | 4 | 0:177, 2:644, 4:175, 6:4 |
| No.17-C1.cc1 | C96 | 0.172 | 1.0 | 2 | 0:155, 2:666, 4:168, 6:10, 8:1 |
| No.170-Cs.cc1 | C96 | 0.188 | 1.0 | 4 | 0:165, 2:661, 4:167, 6:7 |
| No.171-C2.cc1 | C96 | 0.250 | 1.0 | 3 | 0:165, 2:651, 4:173, 6:11 |
| No.172-C1.cc1 | C96 | 0.265 | 1.0 | 3 | 0:181, 2:650, 4:161, 6:8 |
| No.173-C2.cc1 | C96 | 0.172 | 1.0 | 2 | 0:178, 2:626, 4:191, 6:5 |
| No.174-C2.cc1 | C96 | 0.125 | 1.0 | 3 | 0:180, 2:642, 4:168, 6:9, 8:1 |
| No.175-C1.cc1 | C96 | 0.109 | 1.0 | 3 | 0:185, 2:644, 4:160, 6:11 |
| No.176-C2.cc1 | C96 | 0.093 | 1.0 | 2 | 0:188, 2:640, 4:169, 6:3 |
| No.177-C1.cc1 | C96 | 0.094 | 1.0 | 3 | 0:182, 2:627, 4:180, 6:11 |
| No.178-Cs.cc1 | C96 | 0.094 | 1.0 | 2 | 0:198, 2:623, 4:167, 6:11, 8:1 |
| No.179-C2.cc1 | C96 | 0.093 | 1.0 | 3 | 0:203, 2:623, 4:167, 6:6, 8:1 |
| No.18-C1.cc1 | C96 | 0.094 | 1.0 | 2 | 0:162, 2:637, 4:191, 6:9, 8:1 |
| No.180-Cs.cc1 | C96 | 0.156 | 1.0 | 2 | 0:198, 2:632, 4:156, 6:14 |
| No.181-C2.cc1 | C96 | 0.203 | 1.0 | 3 | 0:174, 2:654, 4:164, 6:8 |
| No.182-C2.cc1 | C96 | 0.266 | 1.0 | 3 | 0:176, 2:645, 4:176, 6:3 |
| No.183-D2.cc1 | C96 | 0.172 | 1.0 | 3 | 0:182, 2:675, 4:136, 6:7 |
| No.184-D6h.cc1 | C96 | 0.203 | 1.0 | 2 | 0:188, 2:634, 4:170, 6:8 |
| No.185-D6d.cc1 | C96 | 0.250 | 1.0 | 2 | 0:205, 2:660, 4:132, 6:3 |
| No.186-D2.cc1 | C96 | 0.157 | 1.1 | 3 | 0:182, 2:639, 4:170, 6:9 |
| No.187-D6d.cc1 | C96 | 0.125 | 1.1 | 4 | 0:161, 2:630, 4:200, 6:9 |
| No.19-C1.cc1 | C96 | 0.109 | 1.0 | 2 | 0:153, 2:627, 4:211, 6:9 |
| No.2-D3.cc1 | C96 | 0.109 | 1.0 | 2 | 0:134, 2:623, 4:228, 6:13, 8:2 |
| No.20-C1.cc1 | C96 | 0.094 | 1.0 | 2 | 0:150, 2:645, 4:189, 6:16 |
| No.21-D2.cc1 | C96 | 0.219 | 1.0 | 3 | 0:182, 2:593, 4:207, 6:18 |
| No.22-C1.cc1 | C96 | 0.188 | 1.0 | 3 | 0:154, 2:623, 4:208, 6:15 |
| No.23-C1.cc1 | C96 | 0.219 | 1.0 | 3 | 0:143, 2:664, 4:181, 6:12 |
| No.24-C1.cc1 | C96 | 0.250 | 1.0 | 3 | 0:175, 2:602, 4:214, 6:9 |
| No.25-C1.cc1 | C96 | 0.203 | 1.0 | 2 | 0:134, 2:636, 4:216, 6:12, 8:2 |
| No.26-C1.cc1 | C96 | 0.172 | 1.0 | 3 | 0:170, 2:633, 4:190, 6:7 |
| No.27-C1.cc1 | C96 | 0.140 | 1.0 | 2 | 0:173, 2:630, 4:187, 6:10 |
| No.28-C1.cc1 | C96 | 0.109 | 1.0 | 3 | 0:165, 2:608, 4:213, 6:14 |
| No.29-C1.cc1 | C96 | 0.094 | 1.0 | 2 | 0:165, 2:625, 4:202, 6:8 |
| No.3-D3d.cc1 | C96 | 0.188 | 1.0 | 4 | 0:128, 2:605, 4:248, 6:19 |
| No.30-C2.cc1 | C96 | 0.266 | 1.0 | 4 | 0:155, 2:626, 4:204, 6:15 |
| No.31-C2.cc1 | C96 | 0.250 | 1.0 | 3 | 0:167, 2:625, 4:193, 6:15 |
| No.32-C1.cc1 | C96 | 0.172 | 1.0 | 2 | 0:159, 2:651, 4:179, 6:11 |
| No.33-D3h.cc1 | C96 | 0.141 | 1.0 | 2 | 0:152, 2:593, 4:234, 6:20, 8:1 |
| No.34-C1.cc1 | C96 | 0.109 | 1.0 | 3 | 0:185, 2:626, 4:177, 6:12 |
| No.35-C1.cc1 | C96 | 0.109 | 1.0 | 2 | 0:158, 2:657, 4:176, 6:9 |
| No.36-C1.cc1 | C96 | 0.110 | 1.0 | 3 | 0:170, 2:639, 4:184, 6:7 |
| No.37-C1.cc1 | C96 | 0.094 | 1.0 | 3 | 0:151, 2:632, 4:204, 6:13 |
| No.38-C1.cc1 | C96 | 0.203 | 1.0 | 3 | 0:180, 2:639, 4:174, 6:7 |
| No.39-C1.cc1 | C96 | 0.187 | 1.0 | 2 | 0:164, 2:593, 4:223, 6:19, 8:1 |
| No.4-C2.cc1 | C96 | 0.141 | 1.0 | 3 | 0:117, 2:626, 4:241, 6:16 |
| No.40-C2.cc1 | C96 | 0.125 | 1.0 | 3 | 0:142, 2:640, 4:193, 6:24, 8:1 |
| No.41-C2.cc1 | C96 | 0.109 | 1.0 | 3 | 0:154, 2:644, 4:196, 6:6 |
| No.42-C2.cc1 | C96 | 0.250 | 1.0 | 2 | 0:173, 2:624, 4:192, 6:11 |
| No.43-C1.cc1 | C96 | 0.234 | 1.0 | 2 | 0:145, 2:636, 4:203, 6:16 |
| No.44-C1.cc1 | C96 | 0.141 | 1.0 | 2 | 0:186, 2:635, 4:175, 6:4 |
| No.45-C1.cc1 | C96 | 0.125 | 1.0 | 2 | 0:165, 2:614, 4:209, 6:12 |
| No.46-C2.cc1 | C96 | 0.109 | 1.0 | 3 | 0:158, 2:642, 4:190, 6:10 |
| No.47-C1.cc1 | C96 | 0.094 | 1.0 | 3 | 0:167, 2:652, 4:167, 6:14 |
| No.48-C2.cc1 | C96 | 0.093 | 1.0 | 2 | 0:160, 2:627, 4:199, 6:14 |
| No.49-C1.cc1 | C96 | 0.094 | 1.0 | 2 | 0:147, 2:654, 4:189, 6:10 |
| No.5-C1.cc1 | C96 | 0.250 | 1.0 | 3 | 0:152, 2:628, 4:203, 6:17 |
| No.50-Cs.cc1 | C96 | 0.265 | 1.0 | 2 | 0:157, 2:637, 4:194, 6:11, 8:1 |
| No.51-C1.cc1 | C96 | 0.250 | 1.0 | 2 | 0:148, 2:646, 4:196, 6:9, 8:1 |
| No.52-C2.cc1 | C96 | 0.234 | 1.0 | 3 | 0:159, 2:623, 4:207, 6:9, 8:2 |
| No.53-C1.cc1 | C96 | 0.203 | 1.0 | 3 | 0:167, 2:634, 4:191, 6:8 |
| No.54-C1.cc1 | C96 | 0.250 | 1.0 | 2 | 0:166, 2:650, 4:172, 6:12 |
| No.55-C2.cc1 | C96 | 0.156 | 1.0 | 3 | 0:172, 2:619, 4:203, 6:6 |
| No.56-C1.cc1 | C96 | 0.234 | 1.0 | 2 | 0:152, 2:657, 4:183, 6:8 |
| No.57-C1.cc1 | C96 | 0.250 | 1.0 | 3 | 0:188, 2:651, 4:147, 6:14 |
| No.58-C1.cc1 | C96 | 0.265 | 1.0 | 2 | 0:186, 2:640, 4:166, 6:7, 8:1 |
| No.59-C1.cc1 | C96 | 0.266 | 1.0 | 3 | 0:165, 2:675, 4:149, 6:11 |
| No.6-C2.cc1 | C96 | 0.235 | 1.0 | 3 | 0:126, 2:622, 4:237, 6:15 |
| No.60-C1.cc1 | C96 | 0.140 | 1.0 | 3 | 0:180, 2:626, 4:182, 6:10, 8:2 |
| No.61-C1.cc1 | C96 | 0.187 | 1.0 | 3 | 0:184, 2:638, 4:171, 6:7 |
| No.62-C1.cc1 | C96 | 0.203 | 1.0 | 3 | 0:169, 2:639, 4:178, 6:14 |
| No.63-C1.cc1 | C96 | 0.250 | 1.0 | 3 | 0:173, 2:636, 4:182, 6:9 |
| No.64-C1.cc1 | C96 | 0.219 | 1.0 | 3 | 0:170, 2:644, 4:174, 6:12 |
| No.65-C1.cc1 | C96 | 0.203 | 1.0 | 3 | 0:155, 2:627, 4:207, 6:11 |
| No.66-C1.cc1 | C96 | 0.140 | 1.0 | 2 | 0:154, 2:654, 4:178, 6:14 |
| No.67-C1.cc1 | C96 | 0.250 | 1.0 | 4 | 0:181, 2:634, 4:175, 6:10 |
| No.68-C1.cc1 | C96 | 0.234 | 1.0 | 4 | 0:162, 2:611, 4:219, 6:8 |
| No.69-C1.cc1 | C96 | 0.203 | 1.0 | 3 | 0:160, 2:666, 4:168, 6:6 |
| No.7-C1.cc1 | C96 | 0.141 | 1.0 | 3 | 0:154, 2:614, 4:221, 6:11 |
| No.70-C1.cc1 | C96 | 0.265 | 1.0 | 3 | 0:179, 2:677, 4:134, 6:10 |
| No.71-C1.cc1 | C96 | 0.250 | 1.0 | 2 | 0:191, 2:600, 4:202, 6:7 |
| No.72-C1.cc1 | C96 | 0.172 | 1.0 | 2 | 0:186, 2:625, 4:182, 6:7 |
| No.73-C2.cc1 | C96 | 0.141 | 1.0 | 3 | 0:182, 2:612, 4:197, 6:9 |
| No.74-C1.cc1 | C96 | 0.187 | 1.0 | 3 | 0:159, 2:637, 4:192, 6:11, 8:1 |
| No.75-C1.cc1 | C96 | 0.266 | 1.0 | 3 | 0:193, 2:631, 4:166, 6:10 |
| No.76-C1.cc1 | C96 | 0.281 | 1.0 | 3 | 0:188, 2:614, 4:189, 6:9 |
| No.77-C1.cc1 | C96 | 0.188 | 1.0 | 2 | 0:156, 2:647, 4:188, 6:9 |
| No.78-C1.cc1 | C96 | 0.140 | 1.0 | 3 | 0:156, 2:658, 4:177, 6:9 |
| No.79-C1.cc1 | C96 | 0.140 | 1.0 | 3 | 0:173, 2:634, 4:184, 6:9 |
| No.8-Cs.cc1 | C96 | 0.250 | 1.0 | 3 | 0:118, 2:624, 4:237, 6:20, 8:1 |
| No.80-C2.cc1 | C96 | 0.266 | 1.0 | 2 | 0:153, 2:666, 4:170, 6:11 |
| No.81-Cs.cc1 | C96 | 0.282 | 1.0 | 3 | 0:177, 2:646, 4:165, 6:12 |
| No.82-C2.cc1 | C96 | 0.250 | 1.0 | 3 | 0:162, 2:651, 4:170, 6:17 |
| No.83-C2.cc1 | C96 | 0.156 | 1.0 | 3 | 0:169, 2:645, 4:172, 6:14 |
| No.84-C1.cc1 | C96 | 0.141 | 1.0 | 3 | 0:158, 2:678, 4:158, 6:6 |
| No.85-C1.cc1 | C96 | 0.234 | 1.0 | 2 | 0:153, 2:624, 4:209, 6:14 |
| No.86-C1.cc1 | C96 | 0.266 | 1.0 | 3 | 0:165, 2:607, 4:220, 6:8 |
| No.87-C1.cc1 | C96 | 0.266 | 1.0 | 3 | 0:156, 2:656, 4:175, 6:13 |
| No.88-C1.cc1 | C96 | 0.203 | 1.0 | 2 | 0:161, 2:631, 4:193, 6:15 |
| No.89-Cs.cc1 | C96 | 0.250 | 1.1 | 3 | 0:160, 2:634, 4:195, 6:11 |
| No.9-C2.cc1 | C96 | 0.250 | 1.0 | 3 | 0:157, 2:636, 4:196, 6:11 |
| No.90-C1.cc1 | C96 | 0.172 | 1.0 | 3 | 0:183, 2:628, 4:172, 6:16, 8:1 |
| No.91-C1.cc1 | C96 | 0.125 | 1.0 | 3 | 0:175, 2:663, 4:157, 6:5 |
| No.92-C1.cc1 | C96 | 0.109 | 1.0 | 3 | 0:171, 2:621, 4:200, 6:7, 8:1 |
| No.93-C1.cc1 | C96 | 0.110 | 1.0 | 3 | 0:188, 2:629, 4:168, 6:15 |
| No.94-C1.cc1 | C96 | 0.093 | 1.0 | 3 | 0:169, 2:650, 4:174, 6:7 |
| No.95-C1.cc1 | C96 | 0.094 | 1.0 | 2 | 0:170, 2:628, 4:190, 6:12 |
| No.96-C2.cc1 | C96 | 0.094 | 1.0 | 2 | 0:164, 2:625, 4:197, 6:14 |
| No.97-C1.cc1 | C96 | 0.094 | 1.0 | 3 | 0:185, 2:636, 4:170, 6:8, 8:1 |
| No.98-C1.cc1 | C96 | 0.094 | 1.0 | 3 | 0:173, 2:640, 4:179, 6:8 |
| No.99-C2.cc1 | C96 | 0.094 | 1.0 | 3 | 0:168, 2:660, 4:160, 6:12 |
| No.1-C2.cc1 | C98 | 0.094 | 1.0 | 2 | 0:126, 2:595, 4:260, 6:18, 8:1 |
| No.10-C1.cc1 | C98 | 0.094 | 1.0 | 3 | 0:156, 2:634, 4:198, 6:12 |
| No.100-C1.cc1 | C98 | 0.094 | 1.0 | 2 | 0:153, 2:623, 4:212, 6:12 |
| No.101-C1.cc1 | C98 | 0.125 | 1.0 | 3 | 0:149, 2:645, 4:185, 6:21 |
| No.102-C1.cc1 | C98 | 0.110 | 1.1 | 3 | 0:174, 2:615, 4:202, 6:9 |
| No.103-C1.cc1 | C98 | 0.172 | 1.0 | 3 | 0:159, 2:633, 4:197, 6:11 |
| No.104-C1.cc1 | C98 | 0.281 | 1.0 | 3 | 0:152, 2:659, 4:182, 6:7 |
| No.105-C1.cc1 | C98 | 0.281 | 1.1 | 3 | 0:153, 2:639, 4:197, 6:11 |
| No.106-C1.cc1 | C98 | 0.266 | 1.0 | 3 | 0:152, 2:634, 4:199, 6:14, 8:1 |
| No.107-Cs.cc1 | C98 | 0.313 | 1.0 | 3 | 0:148, 2:629, 4:210, 6:13 |
| No.108-Cs.cc1 | C98 | 0.250 | 1.0 | 3 | 0:181, 2:623, 4:187, 6:9 |
| No.109-C1.cc1 | C98 | 0.250 | 1.0 | 2 | 0:140, 2:659, 4:187, 6:13, 8:1 |
| No.11-C2.cc1 | C98 | 0.329 | 1.0 | 2 | 0:135, 2:619, 4:227, 6:19 |
| No.110-Cs.cc1 | C98 | 0.250 | 1.0 | 2 | 0:152, 2:636, 4:198, 6:13, 8:1 |
| No.111-Cs.cc1 | C98 | 0.203 | 1.0 | 2 | 0:161, 2:665, 4:164, 6:10 |
| No.112-C1.cc1 | C98 | 0.265 | 1.0 | 3 | 0:153, 2:633, 4:203, 6:11 |
| No.113-C2.cc1 | C98 | 0.203 | 1.0 | 3 | 0:151, 2:638, 4:198, 6:13 |
| No.114-C1.cc1 | C98 | 0.265 | 1.0 | 3 | 0:149, 2:640, 4:204, 6:7 |
| No.115-C1.cc1 | C98 | 0.172 | 1.0 | 3 | 0:147, 2:652, 4:192, 6:9 |
| No.116-C1.cc1 | C98 | 0.141 | 1.0 | 2 | 0:163, 2:631, 4:197, 6:9 |
| No.117-Cs.cc1 | C98 | 0.187 | 1.0 | 3 | 0:161, 2:621, 4:193, 6:25 |
| No.118-C1.cc1 | C98 | 0.219 | 1.0 | 3 | 0:160, 2:616, 4:209, 6:15 |
| No.119-Cs.cc1 | C98 | 0.250 | 1.0 | 3 | 0:168, 2:618, 4:194, 6:19, 8:1 |
| No.12-C1.cc1 | C98 | 0.219 | 1.0 | 2 | 0:145, 2:640, 4:204, 6:11 |
| No.120-Cs.cc1 | C98 | 0.187 | 1.0 | 2 | 0:156, 2:632, 4:202, 6:10 |
| No.121-C1.cc1 | C98 | 0.266 | 1.0 | 2 | 0:156, 2:668, 4:167, 6:9 |
| No.122-C1.cc1 | C98 | 0.250 | 1.0 | 3 | 0:153, 2:658, 4:177, 6:12 |
| No.123-C1.cc1 | C98 | 0.156 | 1.0 | 2 | 0:169, 2:604, 4:218, 6:8, 8:1 |
| No.124-C1.cc1 | C98 | 0.172 | 1.0 | 3 | 0:178, 2:630, 4:182, 6:10 |
| No.125-C1.cc1 | C98 | 0.125 | 1.0 | 2 | 0:165, 2:642, 4:177, 6:15, 8:1 |
| No.126-C1.cc1 | C98 | 0.156 | 1.0 | 3 | 0:158, 2:610, 4:214, 6:18 |
| No.127-C2.cc1 | C98 | 0.250 | 1.0 | 3 | 0:153, 2:648, 4:189, 6:10 |
| No.128-C1.cc1 | C98 | 0.265 | 1.0 | 2 | 0:147, 2:658, 4:184, 6:11 |
| No.129-C1.cc1 | C98 | 0.156 | 1.0 | 2 | 0:163, 2:636, 4:190, 6:10, 8:1 |
| No.13-C2v.cc1 | C98 | 0.125 | 1.0 | 3 | 0:147, 2:631, 4:205, 6:17 |
| No.130-C2.cc1 | C98 | 0.109 | 1.0 | 3 | 0:158, 2:656, 4:180, 6:6 |
| No.131-C1.cc1 | C98 | 0.109 | 1.0 | 3 | 0:178, 2:643, 4:169, 6:9, 8:1 |
| No.132-C1.cc1 | C98 | 0.094 | 1.0 | 2 | 0:173, 2:607, 4:211, 6:9 |
| No.133-C1.cc1 | C98 | 0.109 | 1.0 | 3 | 0:166, 2:639, 4:178, 6:16, 8:1 |
| No.134-C1.cc1 | C98 | 0.109 | 1.0 | 3 | 0:152, 2:639, 4:192, 6:17 |
| No.135-C1.cc1 | C98 | 0.094 | 1.0 | 2 | 0:156, 2:633, 4:197, 6:14 |
| No.136-C1.cc1 | C98 | 0.094 | 1.0 | 3 | 0:180, 2:647, 4:160, 6:13 |
| No.137-C1.cc1 | C98 | 0.093 | 1.0 | 2 | 0:186, 2:617, 4:186, 6:11 |
| No.138-C1.cc1 | C98 | 0.110 | 1.0 | 2 | 0:165, 2:635, 4:186, 6:14 |
| No.139-C1.cc1 | C98 | 0.109 | 1.0 | 2 | 0:179, 2:619, 4:190, 6:12 |
| No.14-C1.cc1 | C98 | 0.094 | 1.0 | 2 | 0:153, 2:634, 4:195, 6:18 |
| No.140-C1.cc1 | C98 | 0.094 | 1.0 | 3 | 0:177, 2:640, 4:177, 6:6 |
| No.141-C1.cc1 | C98 | 0.094 | 1.0 | 4 | 0:176, 2:611, 4:198, 6:15 |
| No.142-C1.cc1 | C98 | 0.109 | 1.0 | 3 | 0:169, 2:615, 4:203, 6:13 |
| No.143-C1.cc1 | C98 | 0.094 | 1.0 | 3 | 0:197, 2:623, 4:175, 6:5 |
| No.144-C1.cc1 | C98 | 0.109 | 1.0 | 3 | 0:173, 2:634, 4:182, 6:11 |
| No.145-C1.cc1 | C98 | 0.094 | 1.0 | 3 | 0:166, 2:652, 4:171, 6:11 |
| No.146-C1.cc1 | C98 | 0.094 | 1.0 | 3 | 0:169, 2:647, 4:172, 6:12 |
| No.147-C2.cc1 | C98 | 0.109 | 1.0 | 2 | 0:163, 2:653, 4:173, 6:11 |
| No.148-C3.cc1 | C98 | 0.109 | 1.0 | 3 | 0:160, 2:623, 4:200, 6:16, 8:1 |
| No.149-Cs.cc1 | C98 | 0.109 | 1.0 | 3 | 0:144, 2:647, 4:201, 6:7, 8:1 |
| No.15-C1.cc1 | C98 | 0.094 | 1.0 | 3 | 0:158, 2:638, 4:192, 6:12 |
| No.150-C1.cc1 | C98 | 0.093 | 1.0 | 2 | 0:168, 2:626, 4:197, 6:8, 8:1 |
| No.151-Cs.cc1 | C98 | 0.110 | 1.1 | 3 | 0:146, 2:628, 4:214, 6:12 |
| No.152-C1.cc1 | C98 | 0.125 | 1.0 | 3 | 0:183, 2:625, 4:183, 6:9 |
| No.153-C1.cc1 | C98 | 0.109 | 1.0 | 3 | 0:144, 2:643, 4:201, 6:11, 8:1 |
| No.154-C1.cc1 | C98 | 0.094 | 1.0 | 3 | 0:156, 2:629, 4:206, 6:9 |
| No.155-C1.cc1 | C98 | 0.094 | 1.0 | 3 | 0:166, 2:643, 4:175, 6:16 |
| No.156-C2.cc1 | C98 | 0.094 | 1.0 | 3 | 0:155, 2:631, 4:198, 6:16 |
| No.157-C1.cc1 | C98 | 0.093 | 1.0 | 3 | 0:172, 2:640, 4:181, 6:7 |
| No.158-Cs.cc1 | C98 | 0.094 | 1.1 | 4 | 0:175, 2:610, 4:206, 6:8, 8:1 |
| No.159-C1.cc1 | C98 | 0.109 | 1.1 | 3 | 0:156, 2:638, 4:193, 6:13 |
| No.16-C1.cc1 | C98 | 0.094 | 1.0 | 3 | 0:136, 2:636, 4:216, 6:11, 8:1 |
| No.160-C2.cc1 | C98 | 0.094 | 1.0 | 3 | 0:167, 2:598, 4:222, 6:13 |
| No.161-C1.cc1 | C98 | 0.093 | 1.0 | 3 | 0:171, 2:630, 4:186, 6:13 |
| No.162-C2.cc1 | C98 | 0.188 | 1.1 | 2 | 0:156, 2:642, 4:195, 6:7 |
| No.163-C1.cc1 | C98 | 0.204 | 1.1 | 3 | 0:157, 2:633, 4:201, 6:9 |
| No.164-C1.cc1 | C98 | 0.141 | 1.1 | 3 | 0:161, 2:633, 4:194, 6:12 |
| No.165-C1.cc1 | C98 | 0.125 | 1.1 | 4 | 0:156, 2:657, 4:174, 6:13 |
| No.166-C2.cc1 | C98 | 0.094 | 1.0 | 3 | 0:163, 2:634, 4:193, 6:9, 8:1 |
| No.167-C2v.cc1 | C98 | 0.094 | 1.0 | 3 | 0:173, 2:627, 4:188, 6:12 |
| No.168-C1.cc1 | C98 | 0.141 | 1.1 | 4 | 0:171, 2:653, 4:166, 6:10 |
| No.169-C1.cc1 | C98 | 0.219 | 1.0 | 3 | 0:159, 2:635, 4:198, 6:8 |
| No.17-C1.cc1 | C98 | 0.234 | 1.0 | 2 | 0:147, 2:628, 4:210, 6:15 |
| No.170-C1.cc1 | C98 | 0.235 | 1.0 | 3 | 0:145, 2:666, 4:174, 6:15 |
| No.171-C1.cc1 | C98 | 0.172 | 1.1 | 3 | 0:165, 2:625, 4:202, 6:8 |
| No.172-C1.cc1 | C98 | 0.203 | 1.0 | 2 | 0:148, 2:660, 4:182, 6:10 |
| No.173-C1.cc1 | C98 | 0.219 | 1.0 | 3 | 0:175, 2:624, 4:191, 6:10 |
| No.174-C2.cc1 | C98 | 0.390 | 1.1 | 3 | 0:164, 2:639, 4:189, 6:8 |
| No.175-C1.cc1 | C98 | 0.297 | 1.0 | 3 | 0:157, 2:659, 4:173, 6:11 |
| No.176-C2.cc1 | C98 | 0.297 | 1.0 | 3 | 0:156, 2:644, 4:192, 6:8 |
| No.177-C1.cc1 | C98 | 0.266 | 1.0 | 2 | 0:151, 2:658, 4:171, 6:19, 8:1 |
| No.178-C1.cc1 | C98 | 0.187 | 1.0 | 3 | 0:166, 2:646, 4:175, 6:13 |
| No.179-C1.cc1 | C98 | 0.203 | 1.0 | 2 | 0:166, 2:643, 4:188, 6:3 |
| No.18-Cs.cc1 | C98 | 0.234 | 1.0 | 3 | 0:143, 2:644, 4:200, 6:13 |
| No.180-C1.cc1 | C98 | 0.234 | 1.0 | 3 | 0:164, 2:652, 4:174, 6:10 |
| No.181-C1.cc1 | C98 | 0.313 | 1.0 | 3 | 0:157, 2:638, 4:194, 6:11 |
| No.182-C2.cc1 | C98 | 0.250 | 1.1 | 2 | 0:173, 2:658, 4:160, 6:8, 8:1 |
| No.183-C2.cc1 | C98 | 0.281 | 1.1 | 4 | 0:161, 2:648, 4:178, 6:13 |
| No.184-C1.cc1 | C98 | 0.235 | 1.0 | 3 | 0:177, 2:634, 4:182, 6:7 |
| No.185-Cs.cc1 | C98 | 0.297 | 1.0 | 2 | 0:173, 2:637, 4:177, 6:13 |
| No.186-C1.cc1 | C98 | 0.281 | 1.0 | 3 | 0:181, 2:633, 4:178, 6:8 |
| No.187-C1.cc1 | C98 | 0.172 | 1.0 | 3 | 0:164, 2:621, 4:204, 6:11 |
| No.188-C2.cc1 | C98 | 0.172 | 1.0 | 3 | 0:171, 2:651, 4:168, 6:10 |
| No.189-C2.cc1 | C98 | 0.125 | 1.0 | 3 | 0:162, 2:641, 4:185, 6:11, 8:1 |
| No.19-C1.cc1 | C98 | 0.125 | 1.0 | 2 | 0:153, 2:624, 4:213, 6:10 |
| No.190-C1.cc1 | C98 | 0.093 | 1.0 | 2 | 0:165, 2:654, 4:174, 6:7 |
| No.191-C2.cc1 | C98 | 0.125 | 1.1 | 3 | 0:144, 2:634, 4:204, 6:17, 8:1 |
| No.192-Cs.cc1 | C98 | 0.125 | 1.1 | 3 | 0:157, 2:638, 4:190, 6:15 |
| No.193-Cs.cc1 | C98 | 0.250 | 1.0 | 3 | 0:154, 2:644, 4:186, 6:16 |
| No.194-C2.cc1 | C98 | 0.281 | 1.0 | 2 | 0:158, 2:647, 4:178, 6:17 |
| No.195-C1.cc1 | C98 | 0.265 | 1.0 | 3 | 0:160, 2:634, 4:195, 6:11 |
| No.196-C2.cc1 | C98 | 0.266 | 1.0 | 3 | 0:163, 2:649, 4:175, 6:13 |
| No.197-C1.cc1 | C98 | 0.266 | 1.0 | 3 | 0:181, 2:634, 4:176, 6:9 |
| No.198-Cs.cc1 | C98 | 0.266 | 1.0 | 3 | 0:160, 2:641, 4:188, 6:11 |
| No.199-Cs.cc1 | C98 | 0.203 | 1.0 | 3 | 0:171, 2:650, 4:167, 6:12 |
| No.2-C1.cc1 | C98 | 0.156 | 1.0 | 3 | 0:126, 2:625, 4:223, 6:26 |
| No.20-C1.cc1 | C98 | 0.156 | 1.0 | 3 | 0:169, 2:621, 4:205, 6:5 |
| No.200-C2.cc1 | C98 | 0.172 | 1.0 | 4 | 0:164, 2:640, 4:178, 6:17, 8:1 |
| No.201-C1.cc1 | C98 | 0.171 | 1.1 | 4 | 0:149, 2:613, 4:227, 6:11 |
| No.202-C1.cc1 | C98 | 0.141 | 1.0 | 3 | 0:167, 2:623, 4:198, 6:12 |
| No.203-Cs.cc1 | C98 | 0.156 | 1.0 | 2 | 0:163, 2:644, 4:182, 6:11 |
| No.204-C1.cc1 | C98 | 0.296 | 1.0 | 2 | 0:180, 2:629, 4:179, 6:12 |
| No.205-Cs.cc1 | C98 | 0.234 | 1.0 | 2 | 0:188, 2:632, 4:173, 6:7 |
| No.206-C2v.cc1 | C98 | 0.219 | 1.0 | 3 | 0:153, 2:647, 4:191, 6:9 |
| No.207-C1.cc1 | C98 | 0.219 | 1.0 | 2 | 0:167, 2:658, 4:164, 6:11 |
| No.208-C1.cc1 | C98 | 0.250 | 1.1 | 3 | 0:142, 2:658, 4:192, 6:8 |
| No.209-Cs.cc1 | C98 | 0.265 | 1.0 | 3 | 0:156, 2:653, 4:179, 6:12 |
| No.21-C1.cc1 | C98 | 0.250 | 1.0 | 2 | 0:158, 2:610, 4:222, 6:10 |
| No.210-C1.cc1 | C98 | 0.156 | 1.0 | 3 | 0:188, 2:623, 4:172, 6:17 |
| No.211-C2.cc1 | C98 | 0.125 | 1.0 | 2 | 0:183, 2:642, 4:170, 6:4, 8:1 |
| No.212-C1.cc1 | C98 | 0.110 | 1.0 | 3 | 0:178, 2:655, 4:160, 6:6, 8:1 |
| No.213-C1.cc1 | C98 | 0.093 | 1.0 | 2 | 0:179, 2:623, 4:191, 6:6, 8:1 |
| No.214-C1.cc1 | C98 | 0.094 | 1.0 | 3 | 0:186, 2:633, 4:170, 6:11 |
| No.215-D3.cc1 | C98 | 0.094 | 1.0 | 2 | 0:182, 2:618, 4:190, 6:10 |
| No.216-C1.cc1 | C98 | 0.203 | 1.0 | 3 | 0:189, 2:629, 4:169, 6:13 |
| No.217-C1.cc1 | C98 | 0.203 | 1.1 | 3 | 0:175, 2:636, 4:182, 6:7 |
| No.218-Cs.cc1 | C98 | 0.250 | 1.0 | 3 | 0:171, 2:630, 4:187, 6:12 |
| No.219-C1.cc1 | C98 | 0.234 | 1.1 | 3 | 0:187, 2:629, 4:177, 6:7 |
| No.22-C1.cc1 | C98 | 0.265 | 1.0 | 3 | 0:134, 2:638, 4:208, 6:20 |
| No.220-C3.cc1 | C98 | 0.312 | 1.0 | 3 | 0:167, 2:633, 4:188, 6:12 |
| No.221-C2.cc1 | C98 | 0.219 | 1.1 | 2 | 0:165, 2:654, 4:173, 6:8 |
| No.222-C2.cc1 | C98 | 0.250 | 1.1 | 3 | 0:161, 2:637, 4:188, 6:14 |
| No.223-C1.cc1 | C98 | 0.282 | 1.1 | 3 | 0:148, 2:665, 4:175, 6:11, 8:1 |
| No.224-C1.cc1 | C98 | 0.282 | 1.0 | 3 | 0:172, 2:660, 4:159, 6:9 |
| No.225-C2.cc1 | C98 | 0.297 | 1.0 | 3 | 0:179, 2:633, 4:179, 6:9 |
| No.226-C1.cc1 | C98 | 0.234 | 1.1 | 4 | 0:173, 2:637, 4:177, 6:13 |
| No.227-C1.cc1 | C98 | 0.265 | 1.0 | 3 | 0:187, 2:644, 4:160, 6:9 |
| No.228-C1.cc1 | C98 | 0.172 | 1.0 | 4 | 0:158, 2:660, 4:175, 6:7 |
| No.229-C1.cc1 | C98 | 0.140 | 1.0 | 3 | 0:167, 2:641, 4:187, 6:5 |
| No.23-Cs.cc1 | C98 | 0.250 | 1.0 | 2 | 0:146, 2:636, 4:205, 6:13 |
| No.230-C2.cc1 | C98 | 0.312 | 1.1 | 2 | 0:174, 2:632, 4:185, 6:9 |
| No.231-C2.cc1 | C98 | 0.313 | 1.0 | 3 | 0:146, 2:664, 4:176, 6:14 |
| No.232-C2.cc1 | C98 | 0.235 | 1.1 | 3 | 0:153, 2:648, 4:182, 6:17 |
| No.233-C2.cc1 | C98 | 0.172 | 1.1 | 3 | 0:161, 2:645, 4:182, 6:12 |
| No.234-C1.cc1 | C98 | 0.218 | 1.1 | 3 | 0:167, 2:640, 4:185, 6:8 |
| No.235-C1.cc1 | C98 | 0.312 | 1.0 | 2 | 0:176, 2:632, 4:188, 6:4 |
| No.236-C2.cc1 | C98 | 0.188 | 1.0 | 2 | 0:176, 2:631, 4:180, 6:13 |
| No.237-C1.cc1 | C98 | 0.281 | 1.0 | 3 | 0:191, 2:639, 4:156, 6:14 |
| No.238-C1.cc1 | C98 | 0.281 | 1.0 | 2 | 0:196, 2:629, 4:167, 6:6, 8:2 |
| No.239-Cs.cc1 | C98 | 0.187 | 1.0 | 2 | 0:190, 2:630, 4:168, 6:11, 8:1 |
| No.24-Cs.cc1 | C98 | 0.141 | 1.0 | 3 | 0:153, 2:623, 4:214, 6:10 |
| No.240-C1.cc1 | C98 | 0.110 | 1.0 | 2 | 0:179, 2:625, 4:182, 6:14 |
| No.241-Cs.cc1 | C98 | 0.094 | 1.0 | 3 | 0:180, 2:676, 4:138, 6:6 |
| No.242-C2.cc1 | C98 | 0.094 | 1.0 | 3 | 0:209, 2:616, 4:157, 6:17, 8:1 |
| No.243-C2.cc1 | C98 | 0.109 | 1.0 | 2 | 0:153, 2:645, 4:191, 6:11 |
| No.244-C1.cc1 | C98 | 0.250 | 1.0 | 3 | 0:180, 2:651, 4:157, 6:12 |
| No.245-C1.cc1 | C98 | 0.235 | 1.0 | 2 | 0:173, 2:621, 4:188, 6:18 |
| No.246-C2.cc1 | C98 | 0.297 | 1.0 | 2 | 0:176, 2:635, 4:176, 6:13 |
| No.247-C1.cc1 | C98 | 0.343 | 1.0 | 3 | 0:180, 2:640, 4:175, 6:5 |
| No.248-C2.cc1 | C98 | 0.281 | 1.0 | 3 | 0:184, 2:641, 4:167, 6:8 |
| No.249-C1.cc1 | C98 | 0.187 | 1.0 | 3 | 0:159, 2:636, 4:194, 6:11 |
| No.25-C1.cc1 | C98 | 0.141 | 1.0 | 3 | 0:152, 2:618, 4:217, 6:13 |
| No.250-C1.cc1 | C98 | 0.109 | 1.0 | 2 | 0:173, 2:651, 4:163, 6:13 |
| No.251-C2.cc1 | C98 | 0.125 | 1.0 | 3 | 0:167, 2:675, 4:149, 6:9 |
| No.252-C2.cc1 | C98 | 0.109 | 1.1 | 3 | 0:189, 2:618, 4:183, 6:10 |
| No.253-C3.cc1 | C98 | 0.093 | 1.0 | 4 | 0:162, 2:674, 4:151, 6:13 |
| No.254-C2.cc1 | C98 | 0.094 | 1.0 | 3 | 0:178, 2:648, 4:166, 6:7, 8:1 |
| No.255-C1.cc1 | C98 | 0.094 | 1.0 | 3 | 0:172, 2:661, 4:163, 6:4 |
| No.256-C2.cc1 | C98 | 0.235 | 1.0 | 3 | 0:164, 2:660, 4:165, 6:11 |
| No.257-C2.cc1 | C98 | 0.250 | 1.0 | 3 | 0:174, 2:653, 4:159, 6:13, 8:1 |
| No.258-D3.cc1 | C98 | 0.281 | 1.0 | 2 | 0:164, 2:656, 4:176, 6:4 |
| No.259-Cs.cc1 | C98 | 0.281 | 1.1 | 3 | 0:146, 2:662, 4:178, 6:14 |
| No.26-C1.cc1 | C98 | 0.250 | 1.0 | 3 | 0:154, 2:642, 4:196, 6:8 |
| No.27-C1.cc1 | C98 | 0.156 | 1.0 | 3 | 0:157, 2:634, 4:197, 6:12 |
| No.28-C2.cc1 | C98 | 0.141 | 1.0 | 3 | 0:142, 2:617, 4:220, 6:21 |
| No.29-C1.cc1 | C98 | 0.109 | 1.0 | 3 | 0:149, 2:617, 4:219, 6:15 |
| No.3-C2.cc1 | C98 | 0.110 | 1.0 | 2 | 0:146, 2:611, 4:233, 6:10 |
| No.30-C1.cc1 | C98 | 0.094 | 1.0 | 2 | 0:173, 2:637, 4:183, 6:7 |
| No.31-C1.cc1 | C98 | 0.125 | 1.1 | 3 | 0:153, 2:637, 4:204, 6:6 |
| No.32-C1.cc1 | C98 | 0.250 | 1.0 | 3 | 0:160, 2:618, 4:201, 6:20, 8:1 |
| No.33-C1.cc1 | C98 | 0.250 | 1.0 | 3 | 0:167, 2:599, 4:218, 6:15, 8:1 |
| No.34-C1.cc1 | C98 | 0.187 | 1.0 | 2 | 0:150, 2:629, 4:209, 6:11, 8:1 |
| No.35-C1.cc1 | C98 | 0.203 | 1.0 | 3 | 0:131, 2:629, 4:223, 6:17 |
| No.36-C1.cc1 | C98 | 0.250 | 1.0 | 3 | 0:149, 2:612, 4:222, 6:17 |
| No.37-C1.cc1 | C98 | 0.156 | 1.0 | 3 | 0:167, 2:624, 4:197, 6:12 |
| No.38-C1.cc1 | C98 | 0.140 | 1.0 | 3 | 0:166, 2:618, 4:207, 6:9 |
| No.39-C1.cc1 | C98 | 0.187 | 1.0 | 3 | 0:140, 2:643, 4:210, 6:7 |
| No.4-C1.cc1 | C98 | 0.141 | 1.0 | 3 | 0:119, 2:615, 4:245, 6:20, 8:1 |
| No.40-C1.cc1 | C98 | 0.125 | 1.0 | 3 | 0:175, 2:636, 4:176, 6:13 |
| No.41-C1.cc1 | C98 | 0.125 | 1.0 | 3 | 0:176, 2:611, 4:203, 6:10 |
| No.42-C1.cc1 | C98 | 0.218 | 1.0 | 3 | 0:155, 2:639, 4:189, 6:16, 8:1 |
| No.43-C1.cc1 | C98 | 0.250 | 1.0 | 3 | 0:157, 2:646, 4:182, 6:14, 8:1 |
| No.44-C1.cc1 | C98 | 0.156 | 1.0 | 2 | 0:150, 2:651, 4:184, 6:15 |
| No.45-C1.cc1 | C98 | 0.204 | 1.0 | 3 | 0:142, 2:641, 4:205, 6:12 |
| No.46-C1.cc1 | C98 | 0.219 | 1.0 | 3 | 0:146, 2:634, 4:213, 6:6, 8:1 |
| No.47-C2.cc1 | C98 | 0.156 | 1.0 | 3 | 0:158, 2:635, 4:192, 6:14, 8:1 |
| No.48-C1.cc1 | C98 | 0.234 | 1.0 | 3 | 0:132, 2:653, 4:198, 6:16, 8:1 |
| No.49-C1.cc1 | C98 | 0.172 | 1.0 | 4 | 0:182, 2:621, 4:185, 6:12 |
| No.5-C1.cc1 | C98 | 0.140 | 1.0 | 2 | 0:151, 2:627, 4:198, 6:24 |
| No.50-C1.cc1 | C98 | 0.125 | 1.0 | 3 | 0:157, 2:654, 4:175, 6:14 |
| No.51-C1.cc1 | C98 | 0.125 | 1.0 | 3 | 0:162, 2:615, 4:212, 6:11 |
| No.52-C1.cc1 | C98 | 0.250 | 1.0 | 2 | 0:130, 2:652, 4:205, 6:13 |
| No.53-C1.cc1 | C98 | 0.265 | 1.0 | 3 | 0:175, 2:622, 4:186, 6:17 |
| No.54-C1.cc1 | C98 | 0.266 | 1.0 | 3 | 0:166, 2:599, 4:220, 6:14, 8:1 |
| No.55-C1.cc1 | C98 | 0.250 | 1.0 | 3 | 0:178, 2:637, 4:169, 6:16 |
| No.56-C1.cc1 | C98 | 0.156 | 1.0 | 3 | 0:172, 2:624, 4:187, 6:17 |
| No.57-C1.cc1 | C98 | 0.172 | 1.0 | 3 | 0:178, 2:600, 4:209, 6:13 |
| No.58-C1.cc1 | C98 | 0.250 | 1.0 | 3 | 0:162, 2:633, 4:193, 6:11, 8:1 |
| No.59-C1.cc1 | C98 | 0.297 | 1.0 | 2 | 0:160, 2:655, 4:167, 6:18 |
| No.6-C1.cc1 | C98 | 0.296 | 1.0 | 3 | 0:135, 2:634, 4:213, 6:18 |
| No.60-C1.cc1 | C98 | 0.329 | 1.0 | 3 | 0:161, 2:637, 4:189, 6:13 |
| No.61-C2.cc1 | C98 | 0.234 | 1.0 | 3 | 0:179, 2:630, 4:177, 6:13, 8:1 |
| No.62-C2v.cc1 | C98 | 0.265 | 1.0 | 2 | 0:159, 2:622, 4:209, 6:10 |
| No.63-C1.cc1 | C98 | 0.219 | 1.1 | 3 | 0:176, 2:631, 4:180, 6:13 |
| No.64-C1.cc1 | C98 | 0.297 | 1.0 | 3 | 0:143, 2:632, 4:211, 6:14 |
| No.65-Cs.cc1 | C98 | 0.281 | 1.0 | 2 | 0:167, 2:619, 4:199, 6:15 |
| No.66-D3.cc1 | C98 | 0.234 | 1.0 | 2 | 0:124, 2:607, 4:250, 6:18, 10:1 |
| No.67-C1.cc1 | C98 | 0.187 | 1.0 | 2 | 0:178, 2:620, 4:184, 6:17, 8:1 |
| No.68-C1.cc1 | C98 | 0.172 | 1.0 | 3 | 0:139, 2:629, 4:212, 6:20 |
| No.69-C1.cc1 | C98 | 0.312 | 1.1 | 3 | 0:159, 2:639, 4:193, 6:9 |
| No.7-Cs.cc1 | C98 | 0.265 | 1.0 | 3 | 0:165, 2:637, 4:189, 6:9 |
| No.70-C1.cc1 | C98 | 0.313 | 1.0 | 3 | 0:143, 2:647, 4:202, 6:8 |
| No.71-C2.cc1 | C98 | 0.266 | 1.0 | 2 | 0:163, 2:628, 4:194, 6:15 |
| No.72-C2.cc1 | C98 | 0.265 | 1.0 | 3 | 0:173, 2:624, 4:193, 6:10 |
| No.73-C1.cc1 | C98 | 0.266 | 1.0 | 3 | 0:159, 2:638, 4:191, 6:12 |
| No.74-C2.cc1 | C98 | 0.296 | 1.0 | 3 | 0:160, 2:653, 4:170, 6:16, 8:1 |
| No.75-C1.cc1 | C98 | 0.172 | 1.1 | 3 | 0:159, 2:628, 4:206, 6:7 |
| No.76-C1.cc1 | C98 | 0.312 | 1.0 | 3 | 0:162, 2:632, 4:199, 6:7 |
| No.77-C1.cc1 | C98 | 0.297 | 1.0 | 2 | 0:149, 2:656, 4:187, 6:8 |
| No.78-C1.cc1 | C98 | 0.218 | 1.0 | 3 | 0:145, 2:649, 4:195, 6:11 |
| No.79-C1.cc1 | C98 | 0.235 | 1.0 | 4 | 0:158, 2:668, 4:165, 6:9 |
| No.8-C2.cc1 | C98 | 0.328 | 1.0 | 3 | 0:134, 2:613, 4:228, 6:24, 8:1 |
| No.80-C1.cc1 | C98 | 0.313 | 1.0 | 2 | 0:162, 2:657, 4:170, 6:11 |
| No.81-C1.cc1 | C98 | 0.328 | 1.0 | 3 | 0:165, 2:637, 4:188, 6:10 |
| No.82-C1.cc1 | C98 | 0.297 | 1.0 | 3 | 0:172, 2:615, 4:199, 6:14 |
| No.83-C1.cc1 | C98 | 0.266 | 1.0 | 3 | 0:173, 2:614, 4:199, 6:14 |
| No.84-C1.cc1 | C98 | 0.265 | 1.0 | 2 | 0:161, 2:638, 4:194, 6:7 |
| No.85-C1.cc1 | C98 | 0.328 | 1.0 | 3 | 0:138, 2:646, 4:208, 6:8 |
| No.86-C1.cc1 | C98 | 0.297 | 1.0 | 3 | 0:161, 2:644, 4:185, 6:10 |
| No.87-C1.cc1 | C98 | 0.266 | 1.0 | 3 | 0:185, 2:633, 4:173, 6:9 |
| No.88-C2v.cc1 | C98 | 0.282 | 1.0 | 3 | 0:128, 2:660, 4:199, 6:13 |
| No.89-C2.cc1 | C98 | 0.328 | 1.0 | 3 | 0:173, 2:636, 4:187, 6:4 |
| No.9-C2.cc1 | C98 | 0.297 | 1.0 | 3 | 0:138, 2:623, 4:221, 6:18 |
| No.90-C1.cc1 | C98 | 0.266 | 1.0 | 2 | 0:170, 2:627, 4:182, 6:21 |
| No.91-C2.cc1 | C98 | 0.282 | 1.0 | 3 | 0:148, 2:651, 4:194, 6:7 |
| No.92-C1.cc1 | C98 | 0.219 | 1.0 | 3 | 0:164, 2:633, 4:196, 6:7 |
| No.93-C1.cc1 | C98 | 0.328 | 1.0 | 2 | 0:165, 2:624, 4:199, 6:12 |
| No.94-C1.cc1 | C98 | 0.250 | 1.0 | 2 | 0:139, 2:624, 4:216, 6:21 |
| No.95-C1.cc1 | C98 | 0.313 | 1.0 | 2 | 0:164, 2:662, 4:162, 6:12 |
| No.96-C1.cc1 | C98 | 0.297 | 1.0 | 3 | 0:163, 2:656, 4:168, 6:13 |
| No.97-Cs.cc1 | C98 | 0.265 | 1.0 | 2 | 0:149, 2:610, 4:224, 6:17 |
| No.98-Cs.cc1 | C98 | 0.282 | 1.0 | 3 | 0:153, 2:650, 4:180, 6:17 |
| No.99-Cs.cc1 | C98 | 0.266 | 1.0 | 3 | 0:149, 2:628, 4:205, 6:17, 8:1 |
